# Identification of Callose Synthases in Stinging Nettle and Analysis of Their Expression in Different Tissues

**DOI:** 10.3390/ijms21113853

**Published:** 2020-05-28

**Authors:** Gea Guerriero, Emilie Piasecki, Roberto Berni, Xuan Xu, Sylvain Legay, Jean-Francois Hausman

**Affiliations:** 1 Environmental Research and Innovation (ERIN) Department, Luxembourg Institute of Science and Technology, 5, rue Bommel, Z.A.E. Robert Steichen, L-4940 Hautcharage, Luxembourg; e.piasecki@hubebi.com (E.P.); xuan.xu@list.lu (X.X.); sylvain.legay@list.lu (S.L.); jean-francois.hausman@list.lu (J.-F.H.); 2 Department of Life Sciences, University of Siena, via P.A. Mattioli 4, I-53100 Siena, Italy; roberto.berni@yahoo.com

**Keywords:** Stinging nettle, callose synthases, bioinformatics, gene expression, fibre crops

## Abstract

Callose is an important biopolymer of β-1,3-linked glucose units involved in different phases of plant development, reproduction and response to external stimuli. It is synthesized by glycosyltransferases (GTs) known as callose synthases (CalS) belonging to family 48 in the Carbohydrate-Active enZymes (CAZymes) database. These GTs are anchored to the plasma membrane via transmembrane domains. Several genes encoding CalS have been characterized in higher plants with 12 reported in the model organism *Arabidopsis thaliana*. Recently, the *de novo* transcriptome of a fibre-producing clone of stinging nettle (*Urtica dioica* L.) was published and here it is mined for *CalS* genes with the aim of identifying members differentially expressed in the core and cortical tissues of the stem. The goal is to understand whether specific *CalS* genes are associated with distinct developmental stages of the stem internodes (elongation, thickening). Nine genes, eight of which encoding full-length *CalS*, are identified in stinging nettle. The phylogenetic analysis with CalS proteins from other fibre crops, namely textile hemp and flax, reveals grouping into 6 clades. The expression profiles in nettle tissues (roots, leaves, stem internodes sampled at different heights) reveal differences that are most noteworthy in roots vs. leaves. Two *CalS* are differentially expressed in the internodes sampled at the top and middle of the stem. Implications of their role in nettle stem tissue development are discussed.

## 1. Introduction

The plant cell wall is a natural composite material composed of cellulose, hemicelluloses, pectins, as well as structural proteins and it can be impregnated by the aromatic macromolecule lignin, which confers hydrophobicity [1]. The cells of tissues in rapid expansion/elongation are enveloped by the primary cell wall (composed mainly of cellulose, pectins and hemicelluloses), which is flexible, but at the same time strong to cope with the tensile forces occurring during stretching and expansion [2,3]. Tissues that have stopped elongation start to thicken the cell walls and synthesize the secondary cell wall, which can be impregnated with lignin and contains chiefly cellulose and hemicelluloses (e.g., xylan) [4]. The different composition and mechanical properties of plant cell walls are well exemplified in the stem of higher plants, where a basipetal lignification gradient accompanies the transition from elongation in young tissues at the top, to thickening in older internodes at the bottom [5,6].

Besides these classes of biopolymers, plant cells also synthesize another type of polysaccharide, callose, amorphous and characterized by β-1,3-linked glucose units with some β-1,6 branches [7]. Callose has a transient nature, being deposited and removed during specific stages of plant cell development. Callose is deposited in response to pathogen attack [8] or wounding [9], as well as at the cell and sieve plates; it regulates symplastic connectivity through plasmodesmata, by determining the size exclusion limit (SEL) [10]. Additionally, it is present in the inner cell wall of pollen tubes where it resists tension and compression stress [11,12] and it was also shown to participate in cotton fibre growth [13]. This last aspect is in relation to symplasmic isolation via plasmodesmata gating ensuring the turgor-driven elongation of the fibre cell [14]. 

The symplasmic isolation mediated by callose plugging is also important for the control of morphogens’ diffusion involved in the development of the shoot apical meristem (SAM) [15]. Additionally, the release from dormancy is coordinated by re-establishing symplasmic connectivity in the meristem via the catalytic action of β-1,3-gucanases degrading callose [16]. More recently, callose was shown to have a role in stomata [17] and trichome patterning [18] and its amorphous nature was suggested to be associated with plant biosilicification. The micropores in its amorphous structure offer indeed an ideal environment for silicic acid autocondensation into opaline silica [19]. Fluorescence microscopy using the silica-specific fluorescent tracer [2-(4-pyridyl)-5-((4-(2-dimethylaminoethylaminocarbamoyl)methoxy)phenyl)oxazole]-PDMPO and aniline blue proved the co-localization of callose and silica in horsetail [20,21] and rice [22], as well as thale cress [23]. From the above-mentioned processes, it is thus evident that callose is polyfunctional and that its deposition plays a fundamental role in plant development, biosilicification and in the response to exogenous stresses of biotic nature. 

In this study, the recently published *de novo* transcriptome of stinging nettle [24] was mined for *CalS* genes to study the expression in different tissues and internodes of the stem. Nettle is a bast fibre-producing plant and its cellulosic fibres are characterized by noteworthy lengths that are reached by a mechanism known as intrusive or invasive growth [25,26]. During intrusive growth, the middle lamella of neighbour cells is invaded (hence the adjective “intrusive” or “invasive”) without generating wound responses [27,28]. Callose was not observed in intrusively growing flax fibres [25], a result suggesting that there is a mechanism allowing elongating tissues to recognize endogenous mechanisms of intrusive growth. Callose was nevertheless found in bast fibres on the pulling side of flax stems subjected to gravitropic stimulus: the polymer was deposited in bottlenecks of sausage-like bast fibres in the lower part of the stem where it occluded the lumen [29]. Hence, although callose is not found in intrusively growing fibres, it can be deposited in bast fibres in response to mechanical stimulation. Additionally, it remains to be elucidated whether callose mediates symplasmic isolation and turgor-driven elongation in intrusively growing bast fibres in young stem regions. Identifying *CalS* genes in bast fibre-producing plants is therefore important for researches addressing aspects related to turgor-driven growth and response to gravistimulation.

Here, evidence is provided for the occurrence of at least nine *CalS* genes in stinging nettle whose expression varies in roots and leaves. The corresponding proteins are grouped, together with other members from thale cress, *Cannabis sativa* and flax, into 6 clades. A search for conserved motifs carried out on the promoter regions of the genes from clades 1, 2, 3 and 5 identifies the occurrence of conserved motifs recognized by transcription factors (TFs), in particular members of the Dof (DNA binding with one finger) family. In the stem internodes sampled at different developmental stages (elongation/thickening), two *CalS* genes are differentially expressed. Microscopy reveals accumulation of callose in sieve plates, but not in the bast fibres of the cortical tissues, as previously shown for flax [29].

## 2. Results and Discussion

### 2.1. Bioinformatics and Phylogenetic Analysis of UdCalS

By merging an approach combining searches into the recently reported *de novo* transcriptome and BLAST analyses in the available nettle leaf database at Blast4OneKP, nine putative CalS genes are identified in stinging nettle, eight of which encode full-length proteins (accession numbers MT468366, MT468367, MT468368, MT468369, MT468370, MT468371, MT468372, MT468373, MT468374). The details of the genes (nucleotide and corresponding amino acid length), number of transmembrane domains and presence of conserved domains with relative E-values are provided in Table 1. As can be noticed, with the exception of UdCalS6, all the identified proteins are longer than 1750 amino acids and they have >10-11 transmembrane domains (depending on the software used for prediction). All of the identified full-length proteins contain the glucan synthase superfamily and FKS1 domains, the latter being highly conserved among land plants [30] and homologous to fungal β-1,3-glucan synthases [31]; UdCalS1-3-7-8 are characterized by the occurrence, at the N-terminus, of a Vta1 superfamily domain that is involved in sorting of endosomal protein [30]. As previously reported, Vta1 is absent from nettle CalS9 and 10 [32], as well as from CalS11 and 12.

**Figure 1:**
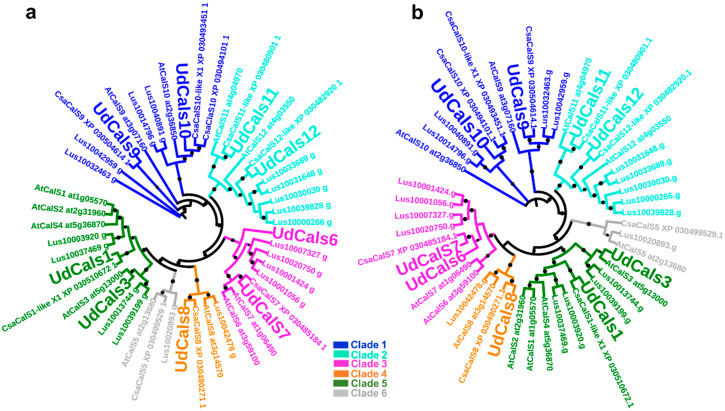
Phylogenetic analysis of CalS (full-length protein sequences) from *U. dioica* (in bigger font size), *A. thaliana*, *L. usitatissimum* and *C. sativa*. (**a**) Balanced minimum evolution tree (bootstraps: 1000); (**b**) Maximum likelihood tree (bootstraps: 100). The circles indicate bootstrap values >800 in (**a**) and >80 in (**b**). The bigger the circle, the higher the bootstrap value. The accession numbers of the proteins from thale cress and *C. sativa* (taken from NCBI) and flax (from Phytozome) are indicated on the trees and in Supplementary File 1.

The phylogenetic analysis of UdCalS proteins with orthologs from thale cress and the fibre crops *C. sativa* and *Linum usitatissimum* (see Supplementary file 1) shows clustering into the previously-reported 6 clades [32] in both maximum likelihood and balanced minimum evolution trees (Figure 1). The 6 groups are composed of UdCalS9-10, UdCalS11-12, UdCalS5, UdCalS1-2-3-4, UdCalS8 and UdCalS6-7 (Figure 1); however, nettle orthologs of cluster 5 were not identified. Interestingly, within clade 6, the hemp ortholog of CalS5 shows the occurrence of a MATE (Multi-antimicrobial extrusion protein)-like superfamily domain (aa 1732-1908) and it is the only glycosyltransferase from family 48 (GT48) with such a domain among the species selected for the phylogenetic analysis.

To understand the potential role of the identified CalS from nettle, a search for conserved motifs was carried out in the promoters of the *CalS* genes in the different clades (comprised between 200–500 bp, sequences in Supplementary File 2). Statistically significant motifs were identified in the promoters of the genes from clade 1-2-3 and 5 (Table 2). The identified conserved motif from clade 1 was submitted to a search against the JASPAR non-redundant sequence database for plants and a match with the binding site of Dof5.8 TF was identified. Dof TFs are zinc-finger regulators distributed among non-vascular and vascular plants that bind to the sequence 5′-AAAG-3′/5′-CTTT-3′ [33]. More specifically, Dof5.8 was shown to play a role in the correct development of veins in the leaves of *Arabidopsis thaliana* [34] and its promoter was demonstrated to be regulated, at least in part, by auxin [35]. Indeed, the overexpression of this TF led to a modification in the expression of several genes involved in auxin biosynthesis, vascular tissue formation, as well as secondary cell wall deposition [34]. Although the approach here used is based entirely on a bioinformatic prediction, the presence of a conserved motif related with the auxin-dependent vascular tissue patterning is noteworthy, especially if one considers the existence of an auxin-plasmodesmata-callose feedback switch regulating plasmodesmata permeability and, ultimately, the symplasmic flux of signalling molecules during physiological processes [36]. It should be noted that GSL8 from thale cress (a.k.a. AtCalS10) is the major player in callose deposition at the plasmodesmata; this protein belongs to clade 1 according to the phylogenetic analyses here shown (Figure 1) and previously reported [32]. 

Genes from clade 2 have a conserved motif (Table 2) showing a match with the TF BASIC PENTACYSTEINE 6 (BPC6). BPC TFs bind GA-rich regions, they are involved in different developmental processes and show widespread expression levels in different organs [37].

The promoters of *CalS* from clade 3 contain three conserved motifs (Table 2). The first one shows similarity to the binding site of the TF Dof1.5 (from thale cress and a.k.a. COG1), a negative regulator of phytochrome A and B signalling [38]. Phytochrome B is the main receptor of red light and mediates defence responses by activating, among others, callose deposition [39]. The potential link of CalS from clade 3 with stress response is corroborated by the other 2 motifs found. The first one shows similarity to the binding sites of SCHLAFMÜTZE (SMZ) from thale cress, an AP2-like ethylene-responsive TF regulating flowering time [40] and downregulated under elevated levels of CO_2_ [41]. The second motif shows similarity to the binding site of the ethylene-responsive transcription factor ERF4 from thale cress and which belongs to a class of repressors implicated in the response to abiotic stress [42].

The promoters of genes from clade 5 have three conserved motifs (Table 2) and all show similarity to Dof TF binding sites. The first shows similarity to the binding site of Dof5.8, the second to COG1/Dof1.5 and the third to *A. thaliana* Dof3.2. Dof3.2 (a.k.a. as Dof6) is a member of the PEAR (PHLOEM EARLY DOF 1) proteins which regulate radial growth of protophloem sieve elements [43]. 

The occurrence of putative motifs recognized by Dof-type TFs in the *CalS* promoters deserves future functional investigation, especially if one considers their reported involvements in cell cycle regulation [44], biotic [45] and abiotic stress response [46], all processes in which callose is known to play an important role [7].

### 2.2. Callose Localization in Stem Tissues and Gene Expression Analysis in Different Organs

**Figure 2:**
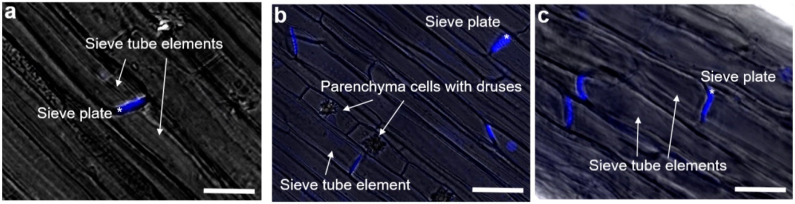
Callose localization at the sieve plates revealed using aniline blue. (**a**) Top, (**b**) middle and (**c**) bottom internode of the stem. The sieve tube elements and phloem parenchyma cells containing crystal druses are indicated with white arrows; sieve plates are indicated with an asterisk. Bars: 10 µm.

To understand whether callose is deposited at different levels in internodes undergoing elongation and thickening, staining with aniline blue was carried out on longitudinal sections of the top, middle and bottom internodes. As can be seen in Figure 2, callose is solely found at sieve plates in the phloem tissue. In young internodes at the top, no callose is deposited at the level of bast fibres, a finding confirming the previous data in the literature that did not report wound responses during elongation of the fibres [27,28]. 

**Figure 3:**
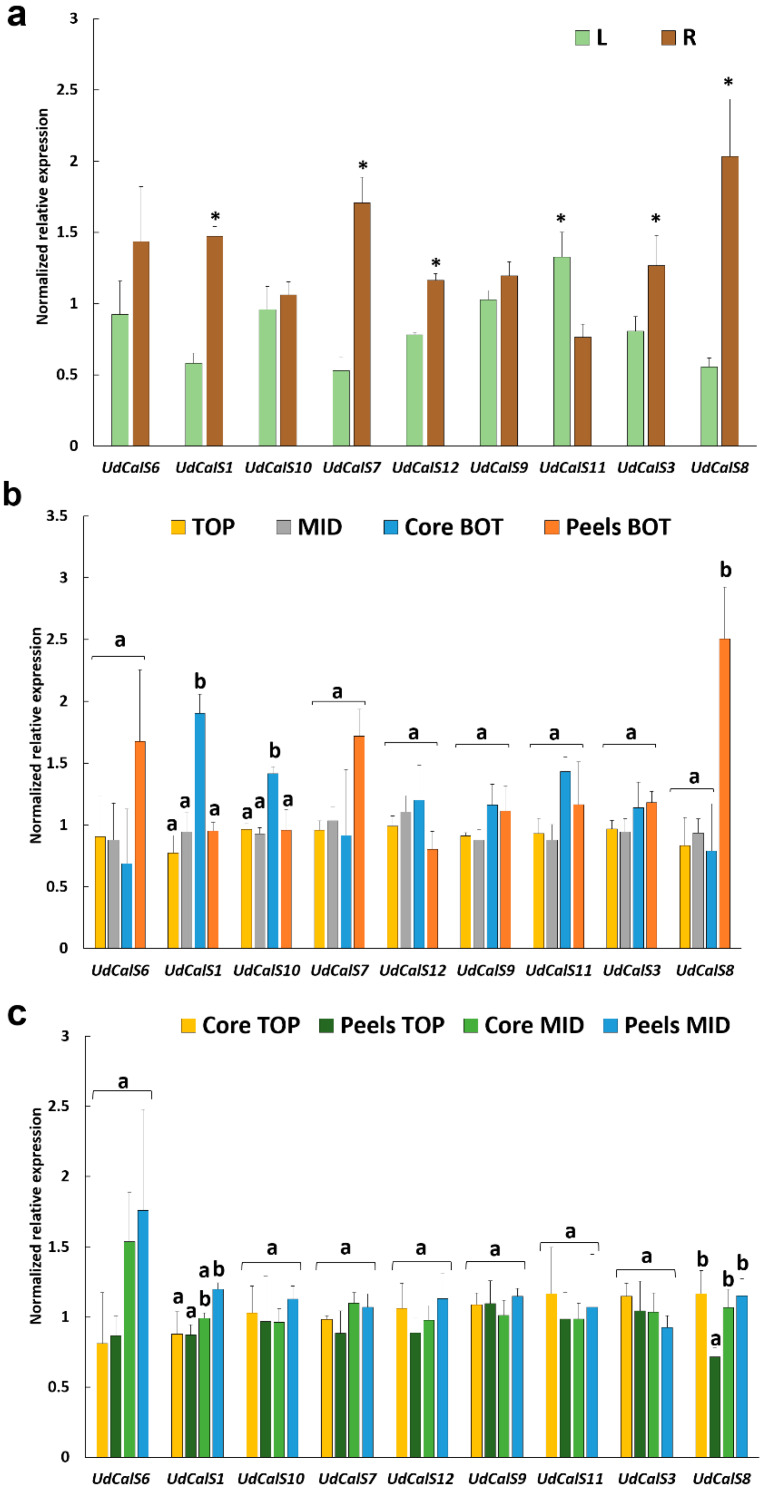
Gene expression analysis (expressed as Normalized relative expression) in (**a**) the leaves and roots, indicated with L/R, (**b**) whole internodes of the top and middle and in the inner and outer tissue of the bottom internode, referred to as Core BOT, Peels BOT and (**c**) in the inner and outer tissue of the top and middle internodes, indicated as Core TOP/MID, Peels TOP/MID. Error bars correspond to the standard deviation calculated from four biological replicates. Asterisks in (**a**) indicate statistically significant differences (*p*-value < 0.05) at the Student’s t-test. Different letters on the vertical bars in (**b**) and (**c**) indicate statistically significant differences (*p*-value < 0.05) among groups. In (**b**) a Kruskal–Wallis test followed by Dunn’s post-hoc test was used for *UdCalS7*-*12*-*11*-*8*, while an ANOVA one-way analysis followed by Tukey’s post-hoc test was applied to all the remaining genes [*UdCalS6* F(3,11) = 2.44, *p*-value = 0.119; *UdCalS1* F(3,11) = 16.54, *p*-value = 0.000; *UdCalS9* F(3,12) = 3.24, *p*-value = 0.60; *UdCalS3* F(3,11) = 1.92, *p*-value = 0.184; *UdCalS10* F(3,11) = 9.44, *p*-value = 0.002]; in (**c**) a Kruskal-Wallis test followed by Dunn’s post-hoc test was used for *UdCalS1*-*11*, while all the other genes were analysed with an ANOVA one-way followed by Tukey’s post-hoc test [*UdCalS6* F(3,10) = 3.01, *p*-value = 0.081; *UdCalS10* F(3,12) = 0.62, *p*-value = 0.661; *UdCalS7* F(3,12) = 2.95, *p*-value = 0.75; *UdCalS12* F(3,12) = 1.53, *p*-value = 0.256; *UdCalS9* F(3,10) = 0.72, *p*-value = 0.562; *UdCalS3* F(3,11) = 1.28, *p*-value = 0.342; *UdCalS8* F(3,12) = 11.81, *p*-value = 0.001].

Gene expression profiling was performed on internodes sampled at different heights, as well as on outer/inner stem tissues, roots and leaves. All the genes studied are expressed in the tissues and organs investigated, although at different levels (Figure 3). In nettle, the majority of *CalS* genes is expressed preferentially in the roots (Figure 3a): nettle has a system of rhizomes allowing it to spread massively by forming an underground network. It should be noted in this respect that, in bamboo, the formation of rhizome buds was accompanied by the induction of a *CalS* gene [47]; it is plausible to assume that *CalS* in nettle are involved in the development of the rhizomes via formation of new buds. 

With the exception of *UdCalS6-9* and *10* that do not show statistically significant changes (for *UdCalS6* a higher variability is present among the root replicates which increases the standard deviation), a higher expression of the *CalS* genes is observed in the roots of stinging nettle (Figure 3a). Only *UdCalS11* is expressed at higher levels in leaves. Out of all the nettle CalS proteins, UdCalS11 shows the highest percentage of identity (60.5%) and query coverage (98%) to wheat (*Triticum aestivum* L.) TaGSL22, whose corresponding gene was reported to be expressed specifically in wheat leaf blade, although at very low levels [48]. The expression level of this gene in nettle leaves is within the same range as the others; it may be involved in basic processes related with leaf growth and expansion.

When whole internodes (i.e., no separation of cortical and core tissues) at the top and middle are analysed, no statistically significant changes are detected (Figure 3b); however, *UdCalS1* and *10* show higher expression in the core tissues of the bottom internode, while *UdCalS8* is expressed at higher levels in the peels at the bottom (Figure 3b). 

When the top and middle internodes are separated into cortical and core tissues, differences can be observed for *UdCalS1* and *8* (Figure 3c), whose expression is not statistically significant when whole internodes are analysed (Figure 3b). This is likely due to the presence of a mixture of cortical and core tissues in the whole internodes. Indeed, for *UdCalS8*, the expression in the cortical peels is significantly lower than the core in the top internode, while in the middle internode there is no difference between core and peels. Hence, when analysing whole internodes at the top and middle of the stem, the differences in expression between cortical and core tissues are not evident. *UdCalS1* is expressed at statistically significant higher levels in the cortical tissues of the middle internode, while *UdCalS8* is downregulated in cortical tissues at the top (Figure 3c). Therefore, in the stem tissues, the *CalS* genes showing differential expression are *UdCalS1*, *10* and *8*. 

Interestingly, among all the nettle CalS, UdCalS8 is the protein showing the highest percentage of identity (58.26%) and query coverage (95%) with TaGSL10, which is a callose synthase displaying stem-specific expression in wheat (*T. aestivum* L.) [48]. 

## 3. Materials and Methods

### 3.1. Plant Growth

Nettle plants (clone 13) were grown as previously described [24,49,50]. To summarize, stem apical cuttings were taken from a donor plant, they were immediately dipped in tap water and subsequently in a commercial root-inducing hormone mix in powder form. The cuttings were put in a mixture of 1/3 sand 2/3 potting soil and then grown in incubators set with a cycle of 16 h light 25 °C/8 h dark 20 °C. When the plants were 35 days old (approximately 70 cm in height), internodes were sampled at the top (immediately below the apex), middle (below the top internode) and bottom (two internodes below the middle). 

The leaves (harvested from younger and older stem regions) were cut and frozen in liquid nitrogen. To sample the roots, the pots were inverted over a bucket, the pots removed and the soil gently shaken to remove the bigger particles; subsequently, the roots were cleaned with tap water, blotted dry, then excised and immediately plunged in liquid nitrogen. The top and middle internodes were either taken as a whole or separated into cortical and core tissues as described previously in hemp [6]. Four independent biological replicates, each consisting of a pool of five homogeneous plants, were used in the study. The tissues were ground to a fine powder using liquid nitrogen and a pre-chilled mortar with a pestle. The ground tissues were stored at –80 °C until RNA extraction.

### 3.2. RNA Extraction and Gene Expression Analysis

RNA was extracted and its purity/concentration checked as previously described [24,49,50]. Primers were designed with Primer3Plus ([51] and available at: http://www.bioinformatics.nl/cgi-bin/primer3plus/primer3plus.cgi) and checked with the OligoAnalyzer Tool from Integrated DNA Technologies (IDT) (available at: https://eu.idtdna.com/pages/tools/oligoanalyzer). Their amplification efficiencies were calculated using serial dilutions, as described previously [24,49,50]. 

The primer list for the expression analysis of nettle *CalS* is provided in Table 3. The primers for the reference genes were previously reported [50]. For qPCR analysis, the method described previously for 384-wells plates was used [24,49,50]. Data were analysed using qBASE^PLUS^ (Biogazelle, Zwijnaarde, Belgium). The reference genes *Cyclophilin* and the translation elongation factor *EF2* were used for normalization in roots and leaves; *Cyclophilin* and the eukaryotic translation initiation factor *eTIF4E* were used to normalize the data obtained on the top, middle and bottom inner/outer tissues; *eTIF4E* and the gene *RAN* coding for a small GTP-binding protein were used to normalize the data of the outer and inner tissues of the top and middle internode. For statistical analyses, the Normalized Relative quantities (NRQ) were log10 transformed. Normal distribution of the data was checked with a Shapiro-Wilk test in IBM SPSS statistics v20 (IBM SPSS, Chicago, IL, USA) and graphically visualized with a Q-Q plot. The homogeneity of the data was checked with the Homogeneity of Variance Test in IBM SPSS statistics v20. For data following normal distribution and homogeneous, a Student’s t-test or one-way ANOVA with Tukey’s post-hoc text were performed. For data that did not follow a normal distribution and/or were not homogeneous, a Kruskal-Wallis test was performed with Dunn’s post-hoc test.

### 3.3. Bioinformatic Analyses

Full-length *CalS* sequences from nettle were reconstructed by merging the contigs of the *de novo* transcriptome [24] and the sequences retrieved in the nettle leaf database at Blast4OneKP (http://db.cngb.org/blast4onekp/home). For the genomic sequences, the *U. dioica* samples in NCBI BioProject number PRJNA602985 were used. For *Cannabis*, thale cress and flax, the sequences upstream of the start codon and subjected to motif search were retrieved from the *Cannabis* genome browser (http://genome.ccbr.utoronto.ca/cgi-bin/hgBlat?command=start) and the Phytozome portal (https://phytozome.jgi.doe.gov/pz/portal.html). For the phylogenetic analyses, CalS protein sequences from thale cress and *Cannabis sativa* were obtained from NCBI, while for *Linum usitatissimum* they were retrieved from the Phytozome portal. The maximum likelihood (ML) tree was generated using iqtree [52] (available at: http://iqtree.cibiv.univie.ac.at/, by setting default parameters and 100 bootstraps), while the balanced minimum evolution tree (FastME) was performed using NGPhylogeny.fr [53] (available at: https://ngphylogeny.fr/) by setting 1000 bootstraps. The trees were visualized with iTOL [54] (available at: https://itol.embl.de/login.cgi).

Transmembrane domain prediction was performed using TMHMM [55], Phobius [56,57] and TOPCONS [58]. Conserved domains were identified with CDART [59] and Batch CD-search [60] (available at: https://www.ncbi.nlm.nih.gov/Structure/bwrpsb/bwrpsb.cgi). 

Motif search in the promoters (length ≤ 500 bp upstream of the start codon) of clade 1-2-3-5 *CalS* from nettle, hemp, flax and thale cress (Supplementary File 2) was performed with the MEME Suite 5.1.1 [61] (accessed on April 18th–19th 2020 and available at: http://meme-suite.org/doc/cite.html?man_type=web). Three motifs between 5–50 bp long were searched. The identified motifs were then analysed with Tomtom [62] (available at http://meme-suite.org/tools/tomtom) for a comparison against the available motifs in the JASPAR CORE plant database 2018 [63].

### 3.4. Tissue Sectioning and Confocal Microscopy 

For confocal microscopy on the internodes, sections of 5 mm were vacuum-impregnated in a solution containing sodium phosphate buffer (0.2 M, pH 7.2), glutaraldehyde (1% *w/v*), paraformaldehyde (2% *w/v*) and caffeine (1% *w/v*). Samples were fixed overnight at 4 °C and subsequently dehydrated in ethanol series (70%−30 min, 70%−60 min, 95%−30 min, 95%−60 min, 100%−30 min). The samples were embedded in resin containing PEG 400 (2% *v/v*) and dimethacrylate ethylene glycol (0.4% *w/v*) and included (Technovit^®^ 7100, Heraeus Kulzer GmbH, Hanau, Germany), as described previously [50]. Longitudinal sections of 10 µm thickness were obtained with a microtome and placed on glass slides. Sections were stained with 0.005% (*w/v*) aniline blue in 0.07 M potassium phosphate buffer (pH 8.0) for 30 min at room temperature in the dark, followed by three rinses with the same potassium phosphate buffer. A drop of 50% glycerol was added on the slides before covering with coverslips. Sections were visualized with a Zeiss LSM 880 confocal microscope (Carl Zeiss AG, Oberkochen, Germany) using a 405 nm laser.

## 4. Conclusions

This study identifies at least nine *CalS* genes in stinging nettle and bioinformatics searches have found putative conserved motifs in their promoters recognized by TFs, in particular of the Dof family.

From the results obtained with qPCR, the *CalS* genes identified are all detected in the different organs and stem tissues of stinging nettle and three are differentially expressed along the stem. The approach used, therefore, allowed the distinction of *CalS* involved in different developmental stages of the stem tissues in stinging nettle.

No *CalS* gene is upregulated in the young internodes at the top, nor is callose detected in regions other than the sieve plates. However, future analyses on sections comprising younger developmental stages of the bast fibre cells in their transition from symplastic to intrusive growth will reveal whether callose is deposited at plasmodesmata to isolate the compartment and favour the onset of turgor-driven expansion.

## Figures and Tables

**Table 1:** Characteristics of the identified *CalS* genes and proteins from stinging nettle. The accession numbers, transcript and protein length, as well as the E-value, amino acid start/end and names of the respective conserved domains are provided together with the number of transmembrane domains predicted with TMHMM, Phobius and TOPCONS.

Gene Name with Accession Numbers	Transcript Length (nt)	Protein Length (aa)	Conserved Domains					Number of Transmembrane Domains		
			From aa	To aa	E-Value	Conserved Protein Domain Family	Short Name	TMHMM	Phobius	TOPCONS
***UdCalS1*** **MT468368**	**5886**	1961	1045	1805	0	pfam02364	Glucan_synthase superfamily	16	17	19
			312	424	9.97 × 10^−53^	pfam14288	FKS1_dom1			
			45	170	5.69 × 10^−14^	pfam04652	Vta1 superfamily			
*UdCalS3* MT468366	5886	1961	1059	1817	0	pfam02364	Glucan_synthase superfamily	17	18	17
			323	436	2.83 × 10^−55^	pfam14288	FKS1_dom1			
			52	179	1.78 × 10^−17^	pfam04652	Vta1			
*UdCalS6* (partial) MT468373	3249	1082	216	961	0	pfam02364	Glucan_synthase superfamily	10	10	11
*UdCalS7* MT468367	5778	1925	1042	1782	0	pfam02364	Glucan_synthase superfamily	15	17	20
			335	445	3.44 × 10^−49^	pfam14288	FKS1_dom1			
			56	191	4.57 × 10^−11^	pfam04652	Vta1 superfamily			
*UdCalS8* MT468374	5913	1970	1102	1834	0	pfam02364	Glucan_synthase superfamily	13	17	17
			346	447	5.48 × 10^−48^	pfam14288	FKS1_dom1			
			65	115	0.00046	pfam04652	Vta1 superfamily			
*UdCalS9* MT468369	5706	1901	1023	1766	0	pfam02364	Glucan_synthase superfamily	16	17	17
			337	446	2.45 × 10^−54^	pfam14288	FKS1_dom1			
*UdCalS10* MT468370	5712	1903	1026	1768	0	pfam02364	Glucan_synthase superfamily	15	15	18
			347	457	4.65 × 10^−52^	pfam14288	FKS1_dom1			
*UdCalS11* MT468372	5406	1801	897	1669	0	pfam02364	Glucan_synthase	13	17	19
			174	287	8.84 × 10^−52^	pfam14288	FKS1_dom1			
*UdCalS12* MT468371	5322	1773	875	1639	0	pfam02364	Glucan_synthase	12	18	19
			169	270	5.78 × 10^−47^	pfam14288	FKS1_dom1			

**Table 2:** Conserved motifs in the promoters of *CalS* from clade 1-2-3 and 5 with similarity to known hits from the JASPAR plants non-redundant database updated to 2018 and corresponding *p*-values. R: A/G, Y: C/T, S: G/C, W: A/T, K: G/T, M: A/C, B: C/G/T, D: A/G/T, H: A/C/T, V: A/C/G, N: any base.

*CalS* Clade	Conserved Motif (Significance of the Motif)	Similarity (JASPAR 2018 Plants Non-Redundant)	*p*-Value
1	TTYKTKTTTKTTYATTTTBWTTCTTWTTTTWAHKMTTWTTSTYTGHATHT (3.7 × 10^−5^)	MA1267.1 (AT5G66940)	6.31 × 10^−8^
2	AMACHWTCTCWHYSTCTCTCTCTCYVHC (1.6 × 10^−5^)	MA1402.1 (BPC6)	3.76 × 10^−11^
3	YCACAYDKCKKCHTCMTCYTSTTCTCTYTTBTVTWYCCTYT (1.5 × 10^−8^) TCCTCYTTMTCGCCYCGCMATGCSTTSTACTGCTWYTKCWKCYAST (9.2 × 10^−8^) GGCGTTAGTTTGTTCTCGTCGATCACTGCCGCTCTGGTATCAGGCGGCGG (3.6 × 10^−2^)	MA1279.1 (COG1) MA0553.1 (SMZ) MA0992.1 (ERF4)	5.57 × 10^−5^ 2.99 × 10^−3^ 1.29 × 10^−6^
5	YATBHWWBTCYCTCTCTTTYTRTTTTTNTTSWYNTNYT (6.2 × 10^−10^) AARDRSDTAAAGSTGAMATCTTTSBYT (7.4 × 10^−4^) TSVCCTTHWGKKTTTRSWTYRTTTTRAT (5.5 × 10^−3^)	MA1267.1 (AT5G66940) MA1404.1 (BPC1) MA1279.1 (COG1)	3.44 × 10^−7^ 3.72 × 10^−7^ 1.38 × 10^−3^

**Table 3:** Details of the primers used for the qPCR assay.

Name	Primer Sequence (5′→3′)	Tm (° C)	R^2^	Efficiency (%)	Amplicon Size (bp)
UdCalS6 Fwd	TTCTCGCTCTCCGTTTCTTC	80.21	0.96	89.32	82
UdCalS6 Rev	ACCATCAAACCCTTGCTACG				
UdCalS1 Fwd	TCACACTCACCGAGAAAACG	82.88	0.99	110.56	138
UdCalS1 Rev	GCCGAAACAGAAGCTGAAAC				
UdCalS10 Fwd	TGGAAGAGGCTTTGTTGTCC	81.81	0.99	111.60	146
UdCalS10 Rev	AGGAAACAGGTCCGTTGTTG				
UdCalS7 Fwd	TGAGGAGGGTGAAACTTTGG	83.01	0.99	109.73	149
UdCalS7 Red	GGCTTGGTTGAAGAGCAAAC				
UdCalS12 Fwd	TTTGGACTCGGAGGAATCAG	85.56	0.99	110.71	139
UdCalS12 Rev	ATCCACGGGATGATGAAGAG				
UdCalS9 Fwd	TGTGGACAAGTTGCGAGAAG	81.14	1	111.43	127
UdCalS9 Rev	CCCCAAAACTTTCAGAGTCG				
UdCalS11 Fwd	TCTTGAACCAGCAGTTCGTG	84.49	0.99	105.96	111
UdCalS11 Rev	TCGTGAGGAAATCCCAGATG				
UdCalS3 Fwd	GCCTGATGCTCCAAAAGTTC	79.66	0.99	109.35	84
UdCalS3 Rev	TGCAAGGAGAAAAGGACCTC				
UdCalS8 Fwd	GTCCACGCCTTTGAAATAGC	83.69	0.99	110.52	145
UdCalS8 Rev	CTCGAACATCGCTCTTTTCC				

## Supplementary Materials

Supplementary Materials can be found at https://www.mdpi.com/1422-0067/21/11/3853/s1.
