# Supplementary material for: Identification of Callose Synthases in Stinging Nettle and Analysis of Their Expression in Different Tissues
Source: Int J Mol Sci. 2020 May 28;21(11):3853. doi: 10.3390/ijms21113853 (PMC7313033; doi:10.3390/ijms21113853)
Supplement: Supplementary file 1 [file ijms-21-03853-s001.zip › Supplementary File 1.docx]

**Sequences used to construct the phylogenetic trees**

**>AtCalS1_at1g05570**

MAQRREPDPPPPQRRILRTQTVGSLGEAMLDSEVVPSSLVEIAPILRVANEVEASNPRVAYLCRFYAFEK

AHRLDPTSSGRGVRQFKTALLQRLERENETTLAGRQKSDAREMQSFYQHYYKKYIQALLNAADKADRAQL

TKAYQTAAVLFEVLKAVNQTEDVEVADEILETHNKVEEKTQIYVPYNILPLDPDSQNQAIMRLPEIQAAV

AALRNTRGLPWTAGHKKKLDEDILDWLQSMFGFQKDNVLNQREHLILLLANVHIRQFPKPDQQPKLDDRA

LTIVMKKLFRNYKKWCKYLGRKSSLWLPTIQQEVQQRKLLYMGLYLLIWGEAANLRFMPECLCYIYHHMA

FELYGMLAGSVSPMTGEHVKPAYGGEDEAFLQKVVTPIYQTISKEAKRSRGGKSKHSVWRNYDDLNEYFW

SIRCFRLGWPMRADADFFCQTAEELRLERSEIKSNSGDRWMGKVNFVEIRSFWHIFRSFDRLWSFYILCL

QAMIVIAWNGSGELSAIFQGDVFLKVLSVFITAAILKLAQAVLDIALSWKARHSMSLYVKLRYVMKVGAA

AVWVVVMAVTYAYSWKNASGFSQTIKNWFGGHSHNSPSLFIVAILIYLSPNMLSALLFLFPFIRRYLERS

DYKIMMLMMWWSQPRLYIGRGMHESALSLFKYTMFWIVLLISKLAFSYYAEIKPLVGPTKDIMRIHISVY

SWHEFFPHAKNNLGVVIALWSPVILVYFMDTQIWYAIVSTLVGGLNGAFRRLGEIRTLGMLRSRFQSIPG

AFNDCLVPQDNSDDTKKKRFRATFSRKFDQLPSSKDKEAARFAQMWNKIISSFREEDLISDREMELLLVP

YWSDPDLDLIRWPPFLLASKIPIALDMAKDSNGKDRELKKRLAVDSYMTCAVRECYASFKNLINYLVVGE

REGQVINDIFSKIDEHIEKETLITELNLSALPDLYGQFVRLIEYLLENREEDKDQIVIVLLNMLELVTRD

IMEEEVPSLLETAHNGSYVKYDVMTPLHQQRKYFSQLRFPVYSQTEAWKEKIKRLHLLLTVKESAMDVPS

NLEARRRLTFFSNSLFMDMPPAPKIRNMLSFSVLTPYFSEDVLFSIFGLEQQNEDGVSILFYLQKIFPDE

WTNFLERVKCGNEEELRAREDLEEELRLWASYRGQTLTKTVRGMMYYRKALELQAFLDMAKDEELLKGYK

ALELTSEEASKSGGSLWAQCQALADMKFTFVVSCQQYSIHKRSGDQRAKDILRLMTTYPSIRVAYIDEVE

QTHKESYKGTEEKIYYSALVKAAPQTKPMDSSESVQTLDQLIYRIKLPGPAILGEGKPENQNHAIIFTRG

EGLQTIDMNQDNYMEEAFKMRNLLQEFLEKHGGVRCPTILGLREHIFTGSVSSLAWFMSNQENSFVTIGQ

RVLASPLKVRFHYGHPDIFDRLFHLTRGGICKASKVINLSEDIFAGFNSTLREGNVTHHEYIQVGKGRDV

GLNQISMFEAKIANGNGEQTLSRDLYRLGHRFDFFRMLSCYFTTIGFYFSTMLTVLTVYVFLYGRLYLVL

SGLEEGLSSQRAFRNNKPLEAALASQSFVQIGFLMALPMMMEIGLERGFHNALIEFVLMQLQLASVFFTF

QLGTKTHYYGRTLFHGGAEYRGTGRGFVVFHAKFAENYRFYSRSHFVKGIELMILLLVYQIFGQSYRGVV

TYILITVSIWFMVVTWLFAPFLFNPSGFEWQKIVDDWTDWNKWIYNRGGIGVPPEKSWESWWEKELEHLR

HSGVRGITLEIFLALRFFIFQYGLVYHLSTFKGKNQSFWVYGASWFVILFILLIVKGLGVGRRRFSTNFQ

LLFRIIKGLVFLTFVAILITFLALPLITIKDLFICMLAFMPTGWGMLLIAQACKPLIQQLGIWSSVRTLA

RGYEIVMGLLLFTPVAFLAWFPFVSEFQTRMLFNQAFSRGLQISRILGGQRKDRSSKNKE

**>AtCalS2_at2g31960**

MAQRKGPDPPPPQRRILRTQTAGNLGEAMLDSEVVPSSLVEIAPILRVANEVEASNPRVAYLCRFYAFEK

AHRLDPTSSGRGVRQFKTALLQRLERENETTLAGRQKSDAREMQSFYQHYYKKYIQALQNAADKADRAQL

TKAYQTAAVLFEVLKAVNQTEDVEVADEILEAHTKVEEKSQIYVPYNILPLDPDSQNQAIMRFPEIQATV

SALRNTRGLPWPAGHKKKLDEDMLDWLQTMFGFQKDNVSNQREHLILLLANVHIRQFPRPEQQPRLDDRA

LTIVMKKLFKNYKKWCKYLGRKSSLWLPTIQQEVQQRKLLYMGLYLLIWGEAANLRFLPECLCYIYHHMA

FELYGMLAGSVSPMTGEHVKPAYGGEDEAFLQKVVTPIYKTIAKEAKRSRGGKSKHSEWRNYDDLNEYFW

SIRCFRLGWPMRADADFFCQTAEELRLDRSENKPKTGDRWMGKVNFVEIRSFWHIFRSFDRMWSFYILSL

QAMIIIAWNGSGKLSGIFQGDVFLKVLSIFITAAILKLAQAVLDIALSWKSRHSMSFHVKLRFIFKAVAA

AIWVVLMPLTYAYSWKTPSGFAETIKNWFGGHQNSSPSFFIIVILIYLSPNMLSTLLFAFPFIRRYLERS

DYKIVMLMMWWSQPRLYIGRGMHESALSLFKYTMFWVVLLISKLAFSFYAEIKPLVKPTKDIMRVHISVY

RWHEFFPHAKSNMGVVIALWSPVILVYFMDTQIWYAIVSTLVGGLNGAFRRLGEIRTLGMLRSRFQSLPE

AFNACLVPNEKSETPKKKGIMATFTRKFDQVPSSKDKEAARFAQMWNKIISSFREEDLISDREMELLLVP

YWADRDLDLIRWPPFLLASKIPIALDMAKDSNGKDRELTKRLSVDSYMTCAVRECYASFKNLINFLVVGE

REGQVINEIFSRIDEHIEKETLIKDLNLSALPDLYGQFVRLIEYLMENREEDKDQIVIVLLNMLEVVTRD

IMDEEVPSMLESTHNGTYVKYDVMTPLHQQRKYFSQLRFPVYSQTEAWKEKIKRLHLLLTVKESAMDVPS

NLEARRRLTFFSNSLFMEMPDAPKIRNMLSFSVLTPYYSEDVLFSIFGLEKQNEDGVSILFYLQKIFPDE

WTNFLERVKCGSEEELRAREELEEELRLWASYRGQTLTKTVRGMMYYRKALELQAFLDMAKDEELMKGYK

ALELTSEDASKSGTSLWAQCQALADMKFTFVVSCQQYSVQKRSGDQRAKDILRLMTTYPSLRVAYIDEVE

QTHKESYKGADEKIYYSALVKAAPQTKSMDSSESVQTLDQVIYRIKLPGPAILGEGKPENQNHSIIFTRG

EGLQTIDMNQDNYMEEAFKMRNLLQEFLVKHGGVRTPTILGLREHIFTGSVSSLAWFMSNQENSFVTIGQ

RVLASPLKVRFHYGHPDVFDRLFHLTRGGVCKASKVINLSEDIFAGFNSTLREGNVTHHEYIQVGKGRDV

GLNQISMFEAKIANGNGEQTLSRDLYRLGHRFDFFRMLSCYFTTIGFYFSTMLTVLTVYVFLYGRLYLVL

SGLEEGLSNQKAFRSNMPLQAALASQSFVQIGFLMALPMMMEIGLERGFHNALIDFVLMQLQLASVFFTF

QLGTKTHYYGRTLFHGGAEYRGTGRGFVVFHAKFAENYRFYSRSHFVKGIELMILLLVYQIFGHAYRGVV

TYILITVSIWFMVVTWLFAPFLFNPSGFEWQKIVDDWTDWNKWIYNRGGIGVPPEKSWESWWEKEIGHLR

HSGKRGIILEIVLALRFFIFQYGLVYQLSTFKQENQSLWIYGASWFVILFILLIVKGLGVGRQRFSTNFQ

LLFRIIKGFVFLTFLGLLITFLALRFLTPKDIFLCMLAFMPTGWGMLLIAQACKPLIQRLGFWSSVRTLA

RGYEILMGLLLFTPVAFLAWFPFVSEFQTRMLFNQAFSRGLQISRILGGQRKDRSSKNKE

**>AtCalS3_at5g13000**

MSATRGGPDQGPSQPQQRRIIRTQTAGNLGESFDSEVVPSSLVEIAPILRVANEVESSNPRVAYLCRFYA

FEKAHRLDPTSSGRGVRQFKTALLQRLEREHDPTLMGRVKKSDAREMQSFYQHYYKKYIQALHNAADKAD

RAQLTKAYQTANVLFEVLKAVNLTQSIEVDREILEAQDKVAEKTQLYVPYNILPLDPDSANQAIMRYPEI

QAAVLALRNTRGLPWPEGHKKKKDEDMLDWLQEMFGFQKDNVANQREHLILLLANVHIRQFPKPDQQPKL

DDQALTEVMKKLFKNYKKWCKYLGRKSSLWLPTIQQEMQQRKLLYMALYLLIWGEAANLRFMPECLCYIY

HHMAFELYGMLAGNVSPMTGENVKPAYGGEEDAFLRKVVTPIYEVIQMEAQRSKKGKSKHSQWRNYDDLN

EYFWSVDCFRLGWPMRADADFFCLPVAVPNTEKDGDNSKPIVARDRWVGKVNFVEIRSFWHVFRSFDRMW

SFYILCLQAMIIMAWDGGQPSSVFGADVFKKVLSVFITAAIMKLGQAVLDVILNFKAHQSMTLHVKLRYI

LKVFSAAAWVIILPVTYAYSWKDPPAFARTIKSWFGSAMHSPSLFIIAVVSYLSPNMLAGVMFLFPLLRR

FLERSNYRIVMLMMWWSQPRLYVGRGMHESAFSLFKYTMFWVLLIATKLAFSYYIEIRPLVAPTQAIMKA

RVTNFQWHEFFPRAKNNIGVVIALWAPIILVYFMDSQIWYAIFSTLFGGIYGAFRRLGEIRTLGMLRSRF

ESLPGAFNDRLIPDGKNQQKKKGIRATLSHNFTEDKVPVNKEKEAARFAQLWNTIISSFREEDLISDREM

DLLLVPYWADRDLDLIQWPPFLLASKIPIALDMAKDSNGKDRELKKRIESDTYMKCAVRECYASFKNIIK

FVVQGNREKEVIEIIFAEVDKHIDTGDLIQEYKMSALPSLYDHFVKLIKYLLDNKEEDRDHVVILFQDML

EVVTRDIMMEDYNISSLVDSSHGGTWHGGMIPLEQQYQLFASSGAIRFPIEPVTEAWKEKIKRIYLLLTT

KESAMDVPSNLEARRRISFFSNSLFMDMPMAPKVRNMLSFSVLTPYYTEEVLFSLRDLETPNEDGVSILF

YLQKIFPDEWNNFLERVKCLSEEELKESDELEEELRLWASYRGQTLTRTVRGMMYYRKALELQAFLDMAM

HEDLMEGYKAVELNSENNSRGERSLWAQCQAVADMKFTYVVSCQQYGIHKRSGDPRAQDILRLMTRYPSL

RVAYIDEVEEPVKDKSKKGNQKVYYSVLVKVPKSTDHSTLAQNLDQVIYRIRLPGPAILGEGKPENQNHA

IIFSRGEGLQTIDMNQDNYMEEALKMRNLLQEFLTKHDGVRHPSILGLREHIFTGSVSSLAWFMSNQETS

FVTIGQRLLANPLRVRFHYGHPDVFDRLFHLTRGGVSKASKVINLSEDIFAGFNSTLREGNVTHHEYIQV

GKGRDVGLNQISMFEAKIANGNGEQTLSRDIYRLGHRFDFFRMMSCYFTTVGFYFSTLITVLTVYIFLYG

RLYLVLSGLEQGLSTQKGIRDNTPLQIALASQSFVQIGFLMALPMLMEIGLERGFRTALSEFVLMQLQLA

PVFFTFSLGTKTHYYGRTLLHGGAKYRSTGRGFVVFHAKFADNYRLYSRSHFVKGLEMMLLLVVYQIFGS

AYRGVLAYLLITISMWFMVGTWLFAPFLFNPSGFEWQKIVDDWTDWNKWINNIGGIGVPAEKSWESWWEE

EQEHLRYSGKRGIVVEILLALRFFIYQYGLVYHLTITEKTKNFLVYGVSWLVIFLILFVMKTVSVGRRRF

SASFQLMFRLIKGLIFMTFIAIIVILITLAHMTIQDIIVCILAFMPTGWGMLLIAQACKPVVHRAGFWGS

VRTLARGYEIVMGLLLFTPVAFLAWFPFVSEFQTRMLFNQAFSRGLQISRILGGHRKDRSSRNKE

**>AtCalS4_at5g36870**

MNQPNRGQILQTVFSHFFPVASPDSELVPSSLHEDITPILRVAKDVEDTNPRSLFLQDLDIKSVDDSINI

LSGHSHALDKANELDPTSSGRDVRQFKNTILQWLEKNNESTLKARQKSSDAHEMQSFYQQYGDEGINDLL

NAGAGSSSSQRTKIYQTAVVLYDVLDAVHRKANIKVAAKILESHAEVEAKNKIYVPYNILPLDPDSKNHA

MMRDPKIVAVLKAIRYTSDLTWQIGHKINDDEDVLDWLKTMFRFQKDNVSNQREHLILLLANVQMRQTQR

QPNLLDDRALDTVMEKLLGNYNKWCNHVGLESSLRFPKDKQQKVVQQRKLLYTGLYLLIWGEAANLRFMP

ECLCYIYHHMAFELFEMLESKGSKKKYKPKNPTYSGKDEDFLTKVVTPVYKTIAEEAKKSGEGKHSEWRN

YDDLNEYFWSKQYLDKLGWPMKANADFFCKTSQQLGLNKSEKKPDLGDGCVGKVNFVEIRTFWHLFRSFD

RMWSFYILSLQAMIIIAWNETSESGGAVFHKVLSVFITAAKLNLFQAFLDIALSWKARHSMSTHVRQRYI

FKAVAAAVWVLLMPLTYAYSHTSIFIVAILIYLSPNMLPEMLLLIPSIRRTLEKSDFRPVKLIMWWSQPE

LYIGRGMHESAWSIYKYMMFWIVLLTSKLAFSYYVEQIKPLMGPTKEIMSVPMPGYWLPEFFPHVKNNRG

VVITLWSPVILVYFMDTQIWYAIVSTLVGGLYGAFRHIGEIQTLGMLRSRFQSLPGAFNACLIPNENTKE

KGIKLAFSRKCHKIPNTNGKEAKQFSQMWNTIINSFREEDLISNRELELLLMSCWAYPDLDFIRWPIFLL

ASKIPIAVDIAKKRNGKHRELKNILAEDNCMSCAVRECYASIKKLLNTLVTGNSDLMLITTVFTIIDTHI

EKDTLLTELNLSVLPDLHGHFVKLTEYVLQNKDKDKIQIVNVLLKILEMVTKDILKEEIKRLHLLLTVKE

SAMDVPSNLEARRRLTFFSNSLFMEMPGAPKIQNMLSFSALTPYYSEDVLFSTFDLEKENDGVSILFYLQ

KIFPDEWKNFLERVKCGTEEELDAIDYLKEEIRLWASYRGQTLTKTVRGMMYYQKALELQAFFDLANERE

LMKGYKSAEASSSGSSLWAECQALADIKFTYVVACQQYSIHKRSGDQRAKDILTLMTTYPSLRVAYIDEV

EQTHIYSKGTSENFYYSALVKAAPQTYSTDSSDSGHMLDQVIYQIKLPGPPIIGEGKPENQNNAIIFTRG

EALQTIDMNQDYYIEEAFKMRNLLQEFLEKNGGVRYPTILGLREHIFTRSVSCLAWFMSNQEHSFVTIGQ

RVLANPLKVRFHYGHPDVFDRVFHLTRGGVSKASKVINLSEDIFAGFNSTLREGTVSHHEYIQVGKGRDV

GLNQISMFEAKIANGSGEQTLSRDLYRLGHQFDFFRMLSCYFTTVGFYFCSMLTVLTVYVFLYGRLYLVL

SGVEKELGNKPMMMEIILASQSFVQIVFLMAMPMIMEIGLERGFYDALFDFVLMQLQLASVFFTFQLGTK

FHYYCKTLLHGGAEYRGTGRGFVVFHAKFAENYRFYSRSHFVKATELGILLLVYHIFGPTYIGLFTISIW

FMVGTWLFAPFLFNPSGFEWHEIVEDWADWKKWIEYDNGGIGVPPEKSWESWWEKDIEHLQHSGKWGIVV

EIFFALRFFIFQYGLVYQLSAFKNKYSSLWVFGASWLLILILLLTVTVLDYARRRLGTEFQLLFRIIKVS

LFLAFMAIFITLMTCRLILPQDVFLCMLALIPTGWGLLLIAQSCKPLIQQPGIWSWVMTLAWVYDLVMGS

LLFIPIAFMAWFPFISEFQTRMLFNQAFSRGLHISRILSGQRKHRSSKNKD

**>AtCalS5_at2g13680**

MAQSSTSHDSGPQGLMRRPSRSAATTVSIEVFDHEVVPASLGTIAPILRVAAEIEHERPRVAYLCRFYAF

EKAHRLDPSSGGRGVRQFKTLLFQRLERDNASSLASRVKKTDGREVESFYQQYYEHYVRALDQGDQADRA

QLGKAYQTAGVLFEVLMAVNKSEKVEAVAPEIIAAARDVQEKNEIYAPYNILPLDSAGASQSVMQLEEVK

AAVAALGNTRGLNWPSGFEQHRKKTGNLDLLDWLRAMFGFQRDNVRNQREHLVCLFADNHIRLTPKPEPL

NKLDDRAVDTVMSKLFKNYKNWCKFLGRKHSLRLPQAAQDIQQRKILYMGLYLLIWGEAANIRFMPECLC

YIFHNMAYELHGLLAGNVSIVTGENIKPSYGGDDEAFLRKVITPIYRVVQTEANKNANGKAAHSDWSNYD

DLNEYFWTPDCFSLGWPMRDDGDLFKSTRDTTQGKKGSFRKAGRTGKSNFTETRTFWHIYHSFDRLWTFY

LLALQAMIILAFERVELREILRKDVLYALSSIFITAAFLRFLQSVLDVILNFPGFHRWKFTDVLRNILKI

VVSLAWCVVLPLCYAQSVSFAPGKLKQWLSFLPQVKGVPPLYIMAVALYLLPNVLAAIMFIFPMLRRWIE

NSDWHIFRLLLWWSQPRIYVGRGMHESQIALIKYTIFWLLLFCCKFAFSYFLQVKLLVKPTNAIMSIRHV

KYKWHEFFPNAEHNYGAVVSLWLPVILVYFMDTQIWYAIFSTICGGVIGAFDRLGEIRTLGMLRSRFQSL

PGAFNTYLVPSDKTRRRGFSLSKRFAEVTAARRTEAAKFSQLWNEIISSFREEDLISDREMDLLLVPYTS

DPSLKLIQWPPFLLASKIPIALDMAAQFRTRDSDLWKRICADEYMKCAVIECYESFKHVLHTLVIGENEK

RIIGIIIKEVESNISKNSFLSNFRMAPLPALCSKFVELVGILKNADPAKRDTVVLLLQDMLEVVTRDMMQ

NENRELVELGHTNKESGRQLFAGTDAKPAILFPPVATAQWHEQISRLHLLLTVKESAMDVPTNLEAQRRI

AFFTNSLFMDMPRAPRVRNMLSFSVLTPYYSEETVYSKNDLEMENEDGVSVVYYLQKIFPDEWTNFLERL

DCKDETSVLESEENILQLRHWVSLRGQTLFRTVRGMMYYRRALKLQAFLDMANETEILAGYKAISEPTEE

DKKSQRSLYTQLEAVADLKFTYVATCQNYGNQKRSGDRRATDILNLMVNNPSLRVAYIDEVEEREGGKVQ

KVFYSVLIKAVDNLDQEIYRIKLPGPAKIGEGKPENQNHALIFTRGEALQAIDMNQDHYLEEALKMRNLL

EEFNEDHGVRAPTILGFREHIFTGSVSSLAWFMSNQETSFVTIGQRVLASPLKVRFHYGHPDVFDRIFHI

TRGGISKASRGINLSEDIFAGFNSTLRRGNVTHHEYIQVGKGRDVGLNQISLFEAKVACGNGEQTLSRDL

YRLGHRFDFFRMMSCYFTTVGFYISSMIVVLTVYAFLYGRLYLSLSGVEEAIVKFAAAKGDSSLKAAMAS

QSVVQLGLLMTLPMVMEIGLERGFRTALSDLIIMQLQLAPVFFTFSLGTKVHYYGRTILHGGSKYRATGR

GFVVKHEKFAENYRMYSRSHFVKGMELMVLLICYRIYGKAAEDSVGYALVMGSTWFLVGSWLFAPFFFNP

SGFEWQKIVDDWDDWNKWISSRGGIGVPANKSWESWWEEEQEHLLHSGFFGKFWEIFLSLRYFIYQYGIV

YQLNLTKESRMGKQHSIIVYGLSWLVIVAVMIVLKIVSMGRKKFSADFQLMFRLLKLFLFIGSVVIVGML

FHFLKLTVGDIMQSLLAFLPTGWALLQISQVARPLMKTVGMWGSVKALARGYEYIMGVVIFMPVTVLAWF

PFVSEFQTRLLFNQAFSRGLQIQRILAGGKKQK

**>AtCalS6_at3g59100**

MEASSSGTAELPRSLSRRAPSRATTMMIDRPNEDASAMDSELVPSSLASIAPILRVANEIEKDNPRVAYL

CRFHAFEKAHRMDATSSGRGVRQFKTYLLHRLEKEEEETKPQLAKNDPREIQAYYQNFYEKYIKEGETSR

KPEEMARLYQIASVLYDVLKTVVPSPKVDYETRRYAEEVERKRDRYEHYNILPLYAVGTKPAIVELPEVK

AAFSAVRNVRNLPRRRIHLPSNTPNEMRKARTKLNDILEWLASEFGFQRGNVANQREHIILLLANADIRK

RNDEEYDELKPSTVTELMDKTFKSYYSWCKYLHSTSNLKFPDDCDKQQLQLIYISLYLLIWGEASNVRFM

PECICYIFHNMANDVYGILFSNVEAVSGETYETEEVIDEESFLRTVITPIYQVIRNEAKRNKGGTASHSQ

WRNYDDLNEYFWSKKCFKIGWPLDLKADFFLNSDEITPQDERLNQVTYGKSKPKTNFVEVRTFWNLFRDF

DRMWIFLVMAFQAMVIVGWHGSGSLGDIFDKDVFKTVLTIFITSAYLTLLQAALDIILNFNAWKNFKFSQ

ILRYLLKFAVAFMWAVLLPIAYSKSVQRPTGVVKFFSTWTGDWKDQSFYTYAVSFYVLPNILAALLFLVP

PFRRAMECSDMRPIKVIMWWAQPKLYVGRGMHEDMFSLFKYTTFWIMLLISKLAFNYYVEILPLITPTKM

IMNLHIGHYQWHEFFPHATNNIGVVIAIWAPIVLVYLMDTQIWYAIFSTLFGGIHGAFSHLGEIRTLGML

RSRFESIPIAFSRTLMPSEDAKRKHADDYVDQKNITNFSQVWNEFIYSMRSEDKISDRDRDLLLVPSSSG

DVSVIQWPPFLLASKIPIAVDMAKDFKGKEDAELFRKIKSDSYMYYAVIESYETLKKIIYALLEDEADRR

VMNQVFLEVDMSMQQQRFIYEFRMSGLPLLSDKLEKFLSILLSDYEDQGTYKSQLINVFQDVIEIITQDL

LVNGHEILERARVHSPDIKNEKKEQRFEKINIHLVRDRCWREKVIRLHLLLSVKESAINVPQNLEARRRI

TFFANSLFMNMPSAPRIRDMLSFSVLTPYYKEDVLYSEEDLNKENEDGISILFYLQKIYPDEWTNYLDRL

KDPKLPEKDKSEFLREWVSYRGQTLARTVRGMMYYRQALELQCYQEVAGEQAEFSVFRAMASNDENQKAF

LERARALADLKFTYVVSCQVYGNQKKSGDIHNRSCYTNILQLMLKYPSLRVAYVDEREETADAKSPKVFY

SVLLKGGDKFDEEIYRIKLPGPPAEIGEGKPENQNHAIIFTRGEALQTIDMNQDNYFEEAFKLRNVLEEF

NKERVGRRKPTILGLREHIFTGSVSSLAWFMSNQESSFVTIGQRILANPLRVRFHYGHPDIFDRIFHITR

GGVSKASKVINLSEDIFGGFNSTLRGGYVTHHEYIQVGKGRDVGLNPISIFEAKVANGNGEQTLSRDVYR

LGHRFDFYRMLSFYFTTIGFYFSSMLTVLTVYAFLYGRMYMVMSGLEKEILRLASPNQLEALEQALATQS

IFQLGFLMVLPMVMEIGLEHGFRSAIVDFFIMQLQLASVFFTFQLGTKSHYYGRTILHGGSKYRPTGRGF

VVFHAKFAENYRLYSRSHFVKGLELLLLLVVYQIYGHSYRSSNLYLYITVSMWFMVGSWLFAPFIFNPSG

FEWQKTVDDWTDWKRWLGDRGGIGIPVEKSWESWWNVEQEHLKHTSIRGRILEITLALRFFIYQYGIVYQ

LNISQRSKSFLVYGLSWVVLLTSLLVLKMVSMGRRRFGTDFQLMFRILKALLFLGFLSVMTILFVVFKLT

LTDLSASVLAFLPTGWAILLIGQVLRSPIKALGVWDSVKELGRAYENIMGLVIFAPIAVLSWFPIVSEFQ

ARLLFNQAFSRGLQISMILAGRKDKATSSHK

**>AtCalS7_at1g06490**

MASTSSGGRGEDGRPPQMQPVRSMSRKMTRAGTMMIEHPNEDERPIDSELVPSSLASIAPILRVANDIDQ

DNARVAYLCRFHAFEKAHRMDPTSSGRGVRQFKTYLLHKLEEEEEITEHMLAKSDPREIQLYYQTFYENN

IQDGEGKKTPEEMAKLYQIATVLYDVLKTVVPQARIDDKTLRYAKEVERKKEQYEHYNILPLYALGAKTA

VMELPEIKAAILAVCNVDNLPRPRFHSASANLDEVDRERGRSFNDILEWLALVFGFQRGNVANQREHLIL

LLANIDVRKRDLENYVEIKPSTVRKLMEKYFKNYNSWCKYLRCDSYLRFPAGCDKQQLSLLYIGLYLLIW

GEASNVRFMPECLCYIFHNMANEVHGILFGNVYPVTGDTYEAGAPDEEAFLRNVITPIYQVLRKEVRRNK

NGKASHSKWRNYDDLNEYFWDKRCFRLKWPMNFKADFFIHTDEISQVPNQRHDQVSHGKRKPKTNFVEAR

TFWNLYRSFDRMWMFLVLSLQTMIIVAWHPSGSILAIFTEDVFRNVLTIFITSAFLNLLQATLDLVLSFG

AWKSLKFSQIMRYITKFLMAAMWAIMLPITYSKSVQNPTGLIKFFSSWVGSWLHRSLYDYAIALYVLPNI

LAAVFFLLPPLRRIMERSNMRIVTLIMWWAQPKLYIGRGMHEEMFALFKYTFFWVMLLLSKLAFSYYVEI

LPLVNPTKLIWDMHVVNYEWHEFFPNATHNIGVIIAIWGPIVLVYFMDTQIWYAIFSTLFGGIYGAFSHL

GEIRTLGMLRSRFKVVPSAFCSKLTPLPLGHAKRKHLDETVDEKDIARFSQMWNKFIHTMRDEDLISDRE

RDLLLVPSSSGDVTVVQWPPFLLASKIPIALDMAKDFKGKEDVDLFKKIKSEYYMHYAVVEAYETVRDII

YGLLQDESDKRIVREICYEVDISIQQHRFLSEFRMTGMPLLSDKLEKFLKILLSDYEEDDYKSQIINVLQ

DIIEIITQDVMVNGHEILERAHLQSGDIESDKKEQRFEKIDLSLTQNISWREKVVRLLLLLTVKESAINI

PQSLEARRRMTFFANSLFMNMPDAPRVRDMLSFSVLTPYYKEDVLYSEEELNKENEDGITILFYLQRIYP

EEWSNYCERVNDLKRNLSEKDKAEQLRQWVSYRGQTLSRTVRGMMYYRVALELQCFQEYTEENATNGGYL

PSESNEDDRKAFSDRARALADLKFTYVVSCQVYGNQKKSSESRDRSCYNNILQLMLKYPSLRVAYIDERE

ETVNGKSQKVFYSVLLKGCDKLDEEIYRIKLPGPPTEIGEGKPENQNHAIIFTRGEALQTIDMNQDNYFE

ECFKMRNVLQEFDEGRRGKRNPTILGLREHIFTGSVSSLAWFMSNQETSFVTIGQRVLANPLRVRFHYGH

PDIFDRIFHITRGGISKASKIINLSEDIFAGYNSTLRGGYVTHHEYIQAGKGRDVGMNQISFFEAKVANG

NGEQTLSRDVYRLGRRFDFYRMLSFYFTTVGFYFSSMITVLTVYVFLYGRLYLVLSGLEKNILQSASVHE

SNALEQALAAQSVFQLGFLMVLPMVMEIGLEKGFRTALGDFIIMQLQLASVFFTFQLGTKAHYFGRTILH

GGSKYRATGRGFVVFHAKFAENYRLYSRSHFVKGLELVILLVVYQVYGTSYRSSSTYMYITFSMWFLVTS

WLFAPFIFNPSGFEWQKTVDDWTDWKRWMGNRGGIGIVLDKSWESWWDIEQEHLKHTNLRGRVLEILLAL

RFLLYQYGIVYHLNIARRHTTFLVYGLSWAILLSVLLVLKMVSMGRRKFGTDFQVMFRILKALLFLGFLS

VMTVLFVVCGLTISDLFASILAFLPTGWAILLIGQALRSVFKGLGFWDSVKELGRAYEYIMGLVIFTPIA

VLSWFPFVSEFQTRLLFNQAFSRGLQISMILAGKKDKETPSTKYLGHTEESFGLEHDTNTFNHYYLWT

**>AtCalS8_at3g14570**

MSHEIVPVDPIDVPSTSYSRPILGPREDSPERATEFTRSLTFREHVSSEPFDSERLPATLASEIQRFLRI

ANLVESEEPRIAYLCRFHAFEIAHHMDRNSTGRGVRQFKTSLLQRLELDEEFTVRRRKEKSDVRELKRVY

HAYKEYIIRHGAAFNLDNSQREKLINARRIASVLYEVLKTVTSGAGPQAIADRESIRAKSEFYVPYNILP

LDKGGVHQAIMHLPEIKAAVAIVRNTRGLPPPEEFQRHQPFLDLFEFLQYAFGFQNGNVANQREHLILLL

SNTIIRQPQKQSSAPKSGDEAVDALMKKFFKNYTNWCKFLGRKNNIRLPYVKQEALQYKTLYIGLYLLIW

GEASNLRFMPECLCYIFHHMAYELHGVLTGAVSMITGEKVAPAYGGGHESFLADVVTPIYMVVQKEAEKN

KNGTADHSMWRNYDDLNEFFWSLECFEIGWPMRPEHDFFCVESSETSKPGRWRGMLRFRKQTKKTDEEIE

DDEELGVLSEEQPKPTSRWLGKTNFVETRSFWQIFRSFDRMWSFFVLSLQALIIMACHDVGSPLQVFNAN

IFEDVMSIFITSAILKLIKGILDIIFKWKARNTMPINEKKKRLVKLGFAAMWTIILPVLYSHSRRKYICY

FTNYKTWLGEWCFSPYMVAVTIYLTGSAIELVLFFVPAISKYIETSNHGIFKTLSWWGQPRLYVGRGMQE

TQVSQFKYTFFWILVLLTKFAFSYAFEIKPLIEPTRLIMKVGVRNYEWHEIFPEVKSNAAAIVAVWAPIM

VVYFMDTQIWYSVYCTIFGGLYGVLHHLGEIRTLGMLRGRFHTLPSAFNASLIPHSTKDEKRRKQRGFFP

FNLGRGSDGQKNSMAKFVLVWNQVINSFRTEDLISNKELDLMTMPLSSEVLSGIIRWPIFLLANKFSTAL

SIAKDFVGKDEVLYRRIRKDEYMYYAVKECYESLKYILQILVVGDLEKKIISGIINEIEESIRQSSLLEE

FKMAELPALHDKCIELVQLLVEGSAEQLQVEKSEELHGKLVKALQDIFELVTNDMMVHGDRILDLLQSRE

GSGEDTGIFMRVIEPQLFESYGEWRCIHFPLPDSASLSEQIQRFLLLLTVKDSAMDIPENLDARRRLSFF

ATSLFMDMPDAPKVRNMMSFSVLTPHYQEDINYSTNELHSTKSSVSIIFYMQKIFPDEWKNFLERMGCDN

LDALKKEGKEEELRNWASFRGQTLSRTVRGMMYCREALKLQAFLDMADDEDILEGYKDVERSNRPLAAQL

DALADMKFTYVVSCQMFGAQKSSGDPHAQDILDLMIKYPSLRVAYVEEREEIVLDVPKKVYYSILVKAVN

GFDQEIYRVKLPGPPNIGEGKPENQNHAIVFTRGEALQTIDMNQDHYLEEAFKMRNLLQEFLRNRGRRPP

TILGLREHIFTGSVSSLAWFMSYQETSFVTIGQRLLANPLRVRFHYGHPDVFDRIFHITRGGISKSSRTI

NLSEDVFAGYNTTLRRGCITYNEYLQVGKGRDVGLNQISKFEAKVANGNSEQTISRDIYRLGQRFDFFRM

LSCYFTTIGFYFSSLISVIGIYIYLYGQLYLVLSGLQKTLILEAKVKNIKSLETALASQSFIQLGLLTGL

PMVMEIGLEKGFLIAFQDFILMQLQLAAFFFTFSLGTKTHYFGRTILHGGAKYRPTGRKVVVFHANFSEN

YRLYSRSHFIKGFELMILLVVYELFKHTSQSNMAYSFITFSVWFMSFTWLCAPFLFNPSGFTWEIIVGDW

RDWNRWIKEQGGIGIQQDKSWQSWWNDEQAHLRGSGVGARCLEIILSLRFFVYQYGLVYHLDITQSNTNI

IVYALSWVVILATFFTVKAVDLGRQLFSTRKHLVFRFFKVFVFVSILTIIITLANICHLSVKDLLVSCLA

FLPTGWGLILIAQAVRPKIEGTSLWEFTQVLARAYDYGMGVVLFAPMAILAWLPIISAFQTRFLFNEAFN

RRLQIQPILAGKKKNR

**>AtCalS9_at3g07160**

MSRAESSWERLVNAALRRDRTGGVAGGNQSSIVGYVPSSLSNNRDIDAILRAADEIQDEDPNIARILCEH

GYSLAQNLDPNSEGRGVLQFKTGLMSVIKQKLAKREVGTIDRSQDILRLQEFYRLYREKNNVDTLKEEEK

QLRESGAFTDELERKTVKRKRVFATLKVLGSVLEQLAKEIPEELKHVIDSDAAMSEDTIAYNIIPLDAPV

TTNATTTFPEVQAAVAALKYFPGLPKLPPDFPIPATRTADMLDFLHYIFGFQKDSVSNQREHIVLLLANE

QSRLNIPEETEPKLDDAAVRKVFLKSLENYIKWCDYLCIQPAWSNLEAINGDKKLLFLSLYFLIWGEAAN

IRFLPECLCYIFHHMVREMDEILRQQVARPAESCMPVDSRGSDDGVSFLDHVIAPLYGVVSAEAFNNDNG

RAPHSAWRNYDDFNEYFWSLHSFELGWPWRTSSSFFQKPIPRKKLKTGRAKHRGKTSFVEHRTFLHLYHS

FHRLWIFLAMMFQALAIIAFNKDDLTSRKTLLQILSLGPTFVVMKFSESVLEVIMMYGAYSTTRRLAVSR

IFLRFIWFGLASVFISFLYVKSLKAPNSDSPIVQLYLIVIAIYGGVQFFFSILMRIPTCHNIANKCDRWP

VIRFFKWMRQERHYVGRGMYERTSDFIKYLLFWLVVLSAKFSFAYFLQIKPLVGPTRMIVKQNNIPYSWH

DFVSRKNYNALTVASLWAPVVAIYLLDIHIFYTIFSAFLGFLLGARDRLGEIRSLEAIHKLFEEFPGAFM

RALHVPLTNRTSDTSHQTVDKKNKVDAAHFAPFWNQIIKSLREEDYITDFEMELLLMPKNSGRLELVQWP

LFLLSSKILLAKEIAAESNSQEEILERIERDDYMKYAVEEVYHTLKLVLTETLEAEGRLWVERIYEDIQT

SLKERNIHHDFQLNKLSLVITRVTALLGILKENETPEHAKGAIKALQDLYDVMRLDILTFNMRGHYETWN

LLTQAWNEGRLFTKLKWPKDPELKALVKRLYSLFTIKDSAAHVPRNLEARRRLQFFTNSLFMDVPPPKSV

RKMLSFSVFTPYYSEVVLYSMAELTKRNEDGISILFYLQKIYPDEWKNFLARIGRDENALEGDLDNERDI

LELRFWASYRGQTLARTVRGMMYYRKALMLQSYLERKAGNDATDAEGFELSPEARAQADLKFTYVVTCQI

YGRQKEDQKPEAVDIALLMQRNEALRIAYIDVVDSPKEGKSHTEYYSKLVKADISGKDKEIYSIKLPGDP

KLGEGKPENQNHAIVFTRGNAIQTIDMNQDNYFEEALKMRNLLEEFDRDHGIRPPTILGVREHVFTGSVS

SLASFMSNQETSFVTLGQRVLAKPLKIRMHYGHPDVFDRVFHITRGGISKASRVINISEDIFAGFNTTLR

QGNVTHHEYIQVGKGRDVGLNQIALFEGKVAGGNGEQVLSRDVYRLGQLLDFFRMMSFFFTTVGFYLCTM

LTVLTVYIFLYGRAYLALSGVGATIRERAILLDDTALSAALNAQFLFQIGVFTAVPMVLGFILEQGFLQA

IVSFITMQFQLCTVFFTFSLGTRTHYFGRTILHGGARYQATGRGFVVKHIKFSENYRLYSRSHFVKAMEV

ILLLVVYLAYGNDEAGAVSYILLTVSSWFLAVSWLFAPYLFNPAGFEWQKVVEDFKEWTNWLFYRGGIGV

KGAESWEAWWEEELSHIRTLSGRIMETILSLRFFIFQYGIVYKLKLQGSDTSFAVYGWSWVAFAMIIVLF

KVFTFSQKISVNFQLLLRFIQGLSLLMALAGIIVAVVLTPLSVTDIFACVLAFIPTGWGILSIACAWKPV

LKRMGMWKSIRSLARLYDALMGMLIFLPVALCSWFPFVSTFQTRMMFNQAFSRGLEISLILAGDNPNSGL

**>AtCalS10_at2g36850**

MARVYSNWDRLVRATLRREQLRNTGQGHERVSSGLAGAVPPSLGRATNIDAILQAADEIQSEDPSVARIL

CEQAYSMAQNLDPNSDGRGVLQFKTGLMSVIKQKLAKRDGASIDRDRDIERLWEFYKLYKRRHRVDDIQK

EEQKWRESGTTFSSNVGEILKMRKVFATLRALIEVLEVLSRDADPNGVGRSIRDELGRIKKADATLSAEL

TPYNIVPLEAQSMTNAIGVFPEVRGAVQAIRYTEHFPRLPVDFEISGQRDADMFDLLEYIFGFQRDNVRN

QREHLVLTLSNAQSQLSIPGQNDPKIDENAVNEVFLKVLDNYIKWCKYLRIRVVYNKLEAIDRDRKLFLV

SLYFLIWGEAANVRFLPECICYIFHNMAKELDAKLDHGEAVRADSCLTGTDTGSVSFLERIICPIYETIS

AETVRNNGGKAAHSEWRNYDDFNEYFWTPACFELSWPMKTESRFLSKPKGRKRTAKSSFVEHRTYLHLFR

SFIRLWIFMFIMFQSLTIIAFRNEHLNIETFKILLSAGPTYAIMNFIECLLDVVLMYGAYSMARGMAISR

LVIRFLWWGLGSAFVVYYYVKVLDERNKPNQNEFFFHLYILVLGCYAAVRLIFGLLVKLPACHALSEMSD

QSFFQFFKWIYQERYFVGRGLFENLSDYCRYVAFWLVVLASKFTFAYFLQIKPLVKPTNTIIHLPPFQYS

WHDIVSKSNDHALTIVSLWAPVLAIYLMDIHIWYTLLSAIIGGVMGAKARLGEIRTIEMVHKRFESFPEA

FAQNLVSPVVKRVPLGQHASQDGQDMNKAYAAMFSPFWNEIIKSLREEDYLSNREMDLLSIPSNTGSLRL

VQWPLFLLCSKILVAIDLAMECKETQEVLWRQICDDEYMAYAVQECYYSVEKILNSMVNDEGRRWVERIF

LEISNSIEQGSLAITLNLKKLQLVVSRFTALTGLLIRNETPDLAKGAAKAMFDFYEVVTHDLLSHDLREQ

LDTWNILARARNEGRLFSRIAWPRDPEIIEQVKRLHLLLTVKDAAANVPKNLEARRRLEFFTNSLFMDMP

QARPVAEMVPFSVFTPYYSETVLYSSSELRSENEDGISILFYLQKIFPDEWENFLERIGRSESTGDADLQ

ASSTDALELRFWVSYRGQTLARTVRGMMYYRRALMLQSFLERRGLGVDDASLTNMPRGFESSIEARAQAD

LKFTYVVSCQIYGQQKQQKKPEATDIGLLLQRYEALRVAFIHSEDVGNGDGGSGGKKEFYSKLVKADIHG

KDEEIYSIKLPGDPKLGEGKPENQNHAIVFTRGEAIQTIDMNQDNYLEEAIKMRNLLEEFHGKHGIRRPT

ILGVREHVFTGSVSSLAWFMSNQETSFVTLGQRVLAYPLKVRMHYGHPDVFDRIFHITRGGISKASRVIN

ISEDIYAGFNSTLRQGNITHHEYIQVGKGRDVGLNQIALFEGKVAGGNGEQVLSRDVYRIGQLFDFFRMM

SFYFTTVGFYVCTMMTVLTVYVFLYGRVYLAFSGADRAISRVAKLSGNTALDAALNAQFLVQIGIFTAVP

MVMGFILELGLLKAIFSFITMQFQLCSVFFTFSLGTRTHYFGRTILHGGAKYRATGRGFVVQHIKFADNY

RLYSRSHFVKAFEVALLLIIYIAYGYTDGGASSFVLLTISSWFLVISWLFAPYIFNPSGFEWQKTVEDFE

DWVSWLMYKGGVGVKGELSWESWWEEEQAHIQTLRGRILETILSLRFFMFQYGIVYKLDLTRKNTSLALY

GYSWVVLVVIVFLFKLFWYSPRKSSNILLALRFLQGVASITFIALIVVAIAMTDLSIPDMFACVLGFIPT

GWALLSLAITWKQVLRVLGLWETVREFGRIYDAAMGMLIFSPIALLSWFPFISTFQSRLLFNQAFSRGLE

ISIILAGNRANVET

**>AtCalS11_at4g04970**

MRRQRPSVATARDAPSLEVYNIIPIHDFLTEHPSLRYPEVRAAAAALRIVGDLPKPPFADFTPRMDLMDW

LGLLFGFQIDNVRNQRENLVLHLANSQMRLQPPPRHPDGLDPTVLRRFRKKLLRNYTNWCSFLGVRCHVT

SPIQSRHQTNAVLNLRRELLYVALYLLIWGESANLRFMPECLCYIFHHMAMELNKVLAGEFDDMTGMPYW

PSFSGDCAFLKSVVMPIYKTVKTEVESSNNGTKPHSAWRNYDDINEYFWSKRALKSLKWPLDYTSNFFDT

TPKSSRVGKTGFVEQRSFWNVYRSFDRLWILLLLYLQAAIIVATSDVKFPWQDRDVEVALLTVFISWAGL

RLLQSVLDASTQYSLVSRETYWLFIRLTLKFVVAVAWTVLFSVFYARIWSQKNKDGVWSRAANERVVTFL

KVVFVYVIPELLALVLFIVPCIRNWVEELNLGVVYFLTWWFYSKTFVGRGMREGLVDNVKYTLFWIIVLA

TKFIFSYFLQIRPLIAPTRALLNLKDATYNWHEFFGSTHRIAVGMLWLPVILVYLMDLQIWYSIYSSLVG

ATIGLFSHLGEIRNIDQLRLRFQFFSSAMQFNLKPEEHLLSPKATMLKKARDAIHRLKLRYGIGQPFNKI

ESSQVEATWFALIWNEIILTFREEDLISDREVELLELPPNCWNIRVIRWPCFLLCNELLLALSQANELCD

APDHWLWSKICSSEYRRCAVMEAFDSIKFVILKIVKNGTEEESILNRLFMEIDENVENEKITEVYKLTVL

LRIHEKLISLLERLMDPEKKVFRIVNILQALYELCAWEFPKTRRSTPQLRQLGLAPISLEADTELLFVNA

INLPPLDDVVFYRQIRRVHTILTSRDPMHNVPKNIEARERLAFFSNSLFMTMPQAPSVEKMMAFSVLTPY

YDEEVMYRQEMLRAENEDGISTLFYLQRIYEDEWVNFLERMRREGAENENDIWSKKVRDLRLWASYRGQT

LSRTVRGMMYYYSALKKLAFLDSASEMDIRMGTQIAPEARRSYYTNDGGDNTLQPTPSQEISRMASGITH

LLKGSEYGSAMMKFTYVVACQVYGQHKARGDHRAEEILFLMKNHDALRIAYVDEVDLGRGEVEYYSVLVK

FDQQLQREVEIYRIRLPGPLKLGEGKPENQNHALIFTRGDAIQTIDMNQDNHFEEALKMRNLLESFKTYY

GIRKPTILGVREKVFTGSVSSLAWFMSAQETSFVTLGQRVLANPLKVRMHYGHPDVFDRFWFVPRGGISK

ASRVINISEDIFAGFNCTLRGGNVTHHEYIQVGKGRDVGLNQISMFEAKVASGNGEQALSRDVYRLGHRL

DFFRMLSFFYTTVGYYFNTMLIVFTVYAFLWGRLYLALSGVEKIAKDRSSSNEALGAILNQQFIIQLGLF

TALPMILENSLERGFLPAVWDFITMQLQLASFFYTFSMGTRTHYFGRTILHGGAKYRATGRGFVVEHKKF

AENYRLYARTHFIKAIELAIILLVYAAYSPLAKSSFVYILMTISSWFLITSWIISPFLFNPSGFDWLKTV

NDFDDFIAWLWSRGGLFTKADQSWFTWWNEEQEHLKTTGVWGKLLEIILDLRFFFFQYSIVYHLRIAENR

TSIGVYLISWGCIIGIVAIYITTIYAQKRYSVKEHIKYRFIQFLVILLTVLVVVMMLQFTKLTVVDLLIS

LLAFVPTGWGLISIAQVLKPFLLSTVVWDTVISVARFYDLFFGLIVMAPVALLSWLPGFQNMQTRILFNE

AFSRGLQISIILAGKKST

**>AtCalS12_at4g03550**

MSLRHRTVPPQTGRPLAAEAVGIEEEPYNIIPVNNLLADHPSLRFPEVRAAAAALKTVGDLRRPPYVQWR

SHYDLLDWLALFFGFQKDNVRNQREHMVLHLANAQMRLSPPPDNIDSLDSAVVRRFRRKLLANYSSWCSY

LGKKSNIWISDRNPDSRRELLYVGLYLLIWGEAANLRFMPECICYIFHNMASELNKILEDCLDENTGQPY

LPSLSGENAFLTGVVKPIYDTIQAEIDESKNGTVAHCKWRNYDDINEYFWTDRCFSKLKWPLDLGSNFFK

SRGKSVGKTGFVERRTFFYLYRSFDRLWVMLALFLQAAIIVAWEEKPDTSSVTRQLWNALKARDVQVRLL

TVFLTWSGMRLLQAVLDAASQYPLVSRETKRHFFRMLMKVIAAAVWIVAFTVLYTNIWKQKRQDRQWSNA

ATTKIYQFLYAVGAFLVPEILALALFIIPWMRNFLEETNWKIFFALTWWFQGKSFVGRGLREGLVDNIKY

STFWIFVLATKFTFSYFLQVKPMIKPSKLLWNLKDVDYEWHQFYGDSNRFSVALLWLPVVLIYLMDIQIW

YAIYSSIVGAVVGLFDHLGEIRDMGQLRLRFQFFASAIQFNLMPEEQLLNARGFGNKFKDGIHRLKLRYG

FGRPFKKLESNQVEANKFALIWNEIILAFREEDIVSDREVELLELPKNSWDVTVIRWPCFLLCNELLLAL

SQARELIDAPDKWLWHKICKNEYRRCAVVEAYDSIKHLLLSIIKVDTEEHSIITVFFQIINQSIQSEQFT

KTFRVDLLPKIYETLQKLVGLVNDEETDSGRVVNVLQSLYEIATRQFFIEKKTTEQLSNEGLTPRDPASK

LLFQNAIRLPDASNEDFYRQVRRLHTILTSRDSMHSVPVNLEARRRIAFFSNSLFMNMPHAPQVEKMMAF

SVLTPYYSEEVVYSKEQLRNETEDGISTLYYLQTIYADEWKNFKERMHREGIKTDSELWTTKLRDLRLWA

SYRGQTLARTVRGMMYYYRALKMLAFLDSASEMDIREGAQELGSVRNLQGELGGQSDGFVSENDRSSLSR

ASSSVSTLYKGHEYGTALMKFTYVVACQIYGSQKAKKEPQAEEILYLMKQNEALRIAYVDEVPAGRGETD

YYSVLVKYDHQLEKEVEIFRVKLPGPVKLGEGKPENQNHAMIFTRGDAVQTIDMNQDSYFEEALKMRNLL

QEYNHYHGIRKPTILGVREHIFTGSVSSLAWFMSAQETSFVTLGQRVLANPLKVRMHYGHPDVFDRFWFL

SRGGISKASRVINISEDIFAGFNCTLRGGNVTHHEYIQVGKGRDVGLNQISMFEAKVASGNGEQVLSRDV

YRLGHRLDFFRMLSFFYTTVGFFFNTMMVILTVYAFLWGRVYLALSGVEKSALADSTDTNAALGVILNQQ

FIIQLGLFTALPMIVEWSLEEGFLLAIWNFIRMQIQLSAVFYTFSMGTRAHYFGRTILHGGAKYRATGRG

FVVEHKGFTENYRLYARSHFVKAIELGLILIVYASHSPIAKDSLIYIAMTITSWFLVISWIMAPFVFNPS

GFDWLKTVYDFEDFMNWIWYQGRISTKSEQSWEKWWYEEQDHLRNTGKAGLFVEIILVLRFFFFQYGIVY

QLKIANGSTSLFVYLFSWIYIFAIFVLFLVIQYARDKYSAKAHIRYRLVQFLLIVLAILVIVALLEFTHF

SFIDIFTSLLAFIPTGWGILLIAQTQRKWLKNYTIFWNAVVSVARMYDILFGILIMVPVAFLSWMPGFQS

MQTRILFNEAFSRGLRIMQIVTGKKSKGDV

**>UdCals1**

MAYNRRSDEPAQRLTRAQTARHIAEPMLDSEVVPSSLVDIAPILRVANEVETENQRVAYLCRFYAFEKAHRLDPKSSGRGVRQFKTALLQRLEREDKTTLEGKVKSDAREMQSFYRKYYKDYIQALHGDKADRTRLTRAYQTAKVLFEVLKAVDRSNVDLPKEILEAQAKVEEKSQLYMPYNILPLDPDSQNHAIMRYPEIQEVVLALRNTRSLPWPKGHKKKVDEDILDWLMHMFGFQEHNVANQREHLILLLANVQMRQYPKNDQKSKLDDRALTDVMKRLFKNYKKWCKYLNRKSSLWLPSIQQEVQQRKLLYMGLYLLIWGEAANLRFMPECLCYIYHHMAFELYGMLAGNVSSYTGEHVKPAYGGEREAFLKKVVTPIYTVIAKEAQRSKVKSKHSQWRNYDDLNEFFWSVECFRIGWPMRSEAAFFCWPPQESQSDKDREREANNGDRWIGKTNFVEIRSFSHIFRSFDRMWIFYILCLQAMIIIAWNGDGNLNGVFEPDVFNKVLSIFITAAVLKLGQAILDIILSWRARKSMSGYVQLRYVLKAITAAAWVIILPVTYSLGLENPSGFAQTMKSWFSNGQGSSSVFIVAVVVYLSPNMLSILFFLFPLIRRSIERSDKKVLTFIMWWSQPRLYVGRGMHESLFSLFLYTMFWILLLVSKLAFSFYVEIRPLANPTKAIMSIHIQGYEWHEFFPNAKHNIGVVVTLWAPIILVYFMDTQIWYAIFSTIFGGIYGAFRRLGEIRTLVMLRSRFQSIPGAFNACLIPKPTNDQAKKRGLKEKFFRKYDEVLSKKDEAAKFSQLWNEIVTSFREEDLISDREKSLLLVPYTADPDLELIRWPPFLLASKIPIALDMAKDSVGKDNELKKRMNYDTYMCCAVRECYLSFKSIINALVLGERERIVINDIFTSIDGHIENGNLIKELDMRALPNLYEQSVKLIKCLLANKKEDKDQVAIILLNMLEIVTRDIMEDEAPSLLDSGSGHGAGGSHAKDESMAPLDQQYRFFGALNFPMTAETDAWKEKIKRLHLLLTEKESAMDVPSNLEARRRISFFSNSLFMDMPEAPKIRNMLSFSVLTPYYKEEVTFSVDLLEKPNEDGVSILFYLQKIFPDEWTNFLERVKCTSEDELRASQELEDELCLWASYRGQTLTKTVRGMMYYRKALELQAFLDMAKDDELMKGYKAAESNYAEQGERSLMVQCQAVADLKFTYVVSCQQYGADKRSGEARAKDILKLMRTYPSLRVAYIDEVEEPISEDGSAEAANESSAGKSSKFKPKVQKVYYSKLVKVPPKRSDSTDSAEAVENLDQLIYQIKLPGPALLGEGKPENQNHAIIFTRGEGLQTIDMNQDNYLEEAFKMRNLLQELLKKHDGVRYPTILGLREHIFTGSVSSLAWFMSNQENSFVTIGQRLLANPLRVRFHYGHPDVFDRLFHLTRGGISKASKIINLSEDIFAGFNSTLREGNVTHHEYIQVGKGRDVGLNQISMFEAKIANGNGEQTLSRDIYRLGHRFDFFRMLSCYFTTVGFYFSTLLSVLTVYVFLYGRLYLVLSGLEVRLQNERAFRDNKPLQVALAAQSFVQIGLLMALPMIMEIGLERGFRNALTDFILMQLQLAPLFFTFSLGTKTHYYGRTLLHGGAEYRGTGRGFVVFHAKFAENYRLYSRTHFVKGIELMVLLLVYHIFGRSYKDVVAYVLLTVSMWFMVATWLFAPFLFNPSGFEWQKIVDDWNDWNKWISNRGGIGVSPEKSWESWWEKEQQHLRHSGKRGSAVEILLALRFFIYQYGLVYHLTLTEKTQSFLVYGISLLLIVVVLMLTKIASSGRRRFSAEFQLLFRLFKGFVFLSFVGMMITLIAVTHLTLRDVVAIILAFLPTGWGLLLIAQACKPVIVKAGFWGSVQTVACWYEIIMGLLMFTPVAFLAWFPFVSEFQTRMLFNQAFSRGLQISRILGGHRKDRSSSSKE

**>UdCals3**

MSASTSRGKTSDQPPQPQRRLQRTQTAGNLGESIFDSEVVPSSLVEIAPILRVANEVESSNPRVAYLCRFYAFEKAHRLDPSSNGRGVRQFKTALLQRLERENDPTLMGRVKKSDAREMQSFYQHYYKKYIQALQNAADKADRAQLTKAYQTANVLFEVLKAVNMTQSMEVDREILEAQDKVAEKTQILVPYNILPLDPDSANQAIMRYPEIQAAVVALRNTRGLPWPKEYNKKKDEDILDWLLAMFGFQKDNVANQREHLILLLANVHIRQFPKPDQQPKLDDRALTEVMKKLFKNYKKWCKYLGRKSSLWLPTIQQEVQQRKLLYMGLYLLIWGEAANLRFMPECLCYIYHHMAFELYGMLAGNVSPMTGENVKPAYGGEEEAFLNKVVTPIYEVIAKEAERSQETRSKHSQWRNYDDLNEYFWSVDCFRLGWPMRADADFFCLLSRQEHESSGDSKQSRKDRWVGKVNFVEIRSFWHVFRSFDRMWSFFILCLQAMIIIAWNGSGQPGSVFSGDEFKRVLSVFITAAVLKLGQAVLDVILSWKSQRSMSFHVKLRYILKVVSAAAWVIVLPVTYAYTWDNPPGFAQTIKGWFGNNSNAPSLFILAVVIYLSPNMLAGVLFVVPFLRRFLERSNYRIVMLMMWWSQPRLYVGRGMHESAFSLFKYTMFWVLLIATKLAFSYYIEIKPLVGPTKAIMEVHIKDFQWHEFFPRAKNNIGVVVALWSPIILVYFMDTQIWYAIFSTLFGGIYGAFRRLGEIRTLGMLRSRFDSLPGVFNARLVPEEKDEPKKKGLRATLSRNFVEIPLPSNKEKGAARFAQLWNKIISNFREEDLISNRERDLLLVPYWADRDLDLIQWPPFLLASKIPIALDMAKDSNGKDKELKKRIEADVYMSCAIRESYASFRNIIKYLVQGRREKVVIEFIFSEVDKHIDEGSLLNGSGFKMIFLPTLYEHFVKLTRLLLENKAEDSNAVVLIFQDMLETVTRDIMMEDHISSLVDSIHGGDGHEGMTPLEDQQYQLFASAGAINFPIDPLTEAWKEKIKRLYLLLTTKESAMDVPSNLEARRRISFFSNSLFMDMPDAPKVRNMLSFSVLTPYYTEEVLFSLHDLEVPNEDGVSILFYLQKIFPDEWENFLERVSCTSEEELKKSDDLEELRLWASYRGQTLTRTVRGMMYYRKALELQAFLDMARDEDLMEGYKAVENSEDQQKGDRSLWAQCQAVADMKFTYVVSCQKYGIHKRSGDPRALDTLRLMTSYPSLRVAYIDEVEQPSKERSNSRSNPKLYYSTLVKALPTKSIDSQEPVQNLDQIIYRIRLPGPAILGEGKPENQNHSIIFTRGEGLQTIDMNQDNYMEEAFKMRNLLQEFLKKHDGVRNPTILGLREHIFTGSVSSLAWFMSNQETSFVTIGQRLLANPLKVRFHYGHPDVFDRLFHLTRGGISKASKVINLSEDIFAGFNSTLREGNVTHHEYIQVGKGRDVGLNQISMFEAKIANGNGEQTLSRDIYRLGHRFDFFRMMSCYFTTIGFYFSTLITVLTVYVFLYGRLYLVLSGLEQGLSSQPGIRDNKPLQVALASQSFVQIGFLMALPMLMEIGLERGFRTALSEFILMQLQLAPVFFTFSLGTKTHYYGRTLLHGGAKYRPTGRGFVVFHAKFADNYRLYSRSHFVKGLELMLLLVVYQIFGESYRGAVAYLLITISMWFMVGTWLFAPFLFNPSGFEWQKIVDDWSDWNKWISNRGGIGVPPEKSWESWWEEEQEHLRHSGKRGIIVEILLAVRFFIYQYGLVYHLSISEKTRSFLVYGFSWLVILVILFIMKTVSVGRRRFSANFQLMFRLIKGLIFLTFVSIIVTLIAVARMSVQDIIVCILAFMPTGWGILLIAQAVKPVIRTAGFWGSIRTLARGYEIIMGLLLFTPVAFLAWFPFVSEFQTRMLFNQAFSRGLQISMILTGRKDKINSVDKPSTA

**>UdCals6**

MRMEDLLSNRERDLLLAPRDISGDISVFQWPPYLLASKISMAIDIAGHNRGRDDVGLFRKIKSDKYMFSAVVEFYEGIRSVLYALLGDDSNQMIVRSIFMNIEESIQMNRFLNDFRMYALFSLSDSLTKLLRQLLSDDEKDESFVPRIVKALQDAMETVIHDFMINGHEILERDYRSKTEERFGAINRSILNNESVKKKVERLHMILTVRESAKSVPMNMEARRRITFFADSLCMRMPSAPTVRNMFSFSVLTPYYKEDVLYTDEELIKENKDGISILFYMKKIYPDEWCNFMERISNDSMYSNKEKTELTRQWVSYRGQTLFRTVRGIMYHRQALELQCFLEYIERNGLRHRLNSDSRAENVSIQKAVKAVADLKFTYVVSCQIYDALKKSEDPREKNCYRNILNLMLTYPSLRVAYIHAALEPVEGKYQKVYYSVLVKGGDSFEEEIYRIKLPGPPTDIGEAKAENQNHAIIFTRGEALQKIDTNQDSNFGEAFKLRNVLEEFKIHHTNRKPTILGIRDRIFTGSISSLSWLMSNQENSSATIGQRILANPLRVRFHYGHNDIFDRLFHITRGGVSKASKVINRVKDDVFAGFNSTLRGGLVTHHEYIQMGRWCDVGINQISLSDTTDASGNGEQTMSRDVHRLGRCFDFFRMLSLYYTTVGFYFSTMMAVVTVYVFLYGRLYIIMSGVEKEILKSETIQKNMALVETLATQFVIQMGLFLTLPMVIEIGLEKAFRTAVGDFVFMQLQLAPVFFTFRMGTKAHHYGKALLYGTSSKAIIRSEEETTTTFVVPRATFTENYRLFSRSHFVKGLELLVLLIVCHAYGDKLRRSTNYVFSLASDVSLWLLVVSWLFAPFLFSPSCFDWLATVEKWREWESWIENEGGIGVAPEKSWESWWEGEHEYLNYMSLRGKSLVVFLALRFFVYQYGIVYHLDIAHRSKGLMVYGLSWVFTIFTLLVLKIAFTKRQSYKAYNIYKAIMYFAIIAVVTDLFLVYGLTVTDVFAALLAFLPSAWAFVLIGQVCRGLSTRIGVWESVKELAKACDFVMGSVIFGPIAVLSWFPIVSKLQKRLLFYQEFIRGF

**>UdCalS7**

MASSSGTKSDVGPPRTLSRGMTRMPTRMVDLPNEENAAIDSELVPSSLASIAPILRVANEIQRDNPRVAYLCRFHAFEKAHLMDPTSGGRGVRQFKTYLLHRLEKEEIEAKHKLAMNDPKEILFFYQEFYEINIREGEFTKKPEEMAKICQIATVLYEVLKTVVPANQIDPQTQRIADEIAKKRDQYAHYNILPLYAVGVKPAIMELHEIKAALNALRNVDNLPRPRVQYRHPQEDNMGVHRERVQLVNDILDWLSSIFGFQKGNVANQREHLILLLANIDVRGRNPTNHTQLASSTVHSLGEKIFKNYRSWCNYLHCKLNLRCPVGVEDQQMNLIYIALYLLIWGEASNIRFMPECLCYIFHNMANEVYGILDSNTHSVSGATYQNEPRDEEYFLRTVITPIFEVLHKEAKRSRGGKESHTSWRNYDDLNEYFWSQKCLSLGWPMNPKADFFRHSDGVQPTNTRPNQVPIGRKKPKVNFAEVRTFLHLYRNFDRMWMFFILAFQAMLIIAWNSSGSIADLFNEDVFKSVLSIFITSAVLNFVQATLDIVLSFNAWRSLKITQVVRFLLKFVIAAFWVVFLPVCYFRSVQNPTAIERLFSSWTGAWWDQSFYNYAVAIYLIPNILATLLFILPPMRRNMERSNIKVIVFLMWWAQPKLYVGRGMHEDMFTLLKYTLFWIMLLISKLAFSYYVEILPLVEPTKVIMEMKINDYQWHEFFPHAPHNIFVIIAIWTPIVLVYFMDAQIWYAIFSTLFGGIHGAFSHLGEIRTLGMLRSRFAAVPYAFSVNLMPVADGTRKKNLDSEVVRKNIANFSQVWNKVIHTMRLEDLISNRDRDLLLVPYYSNDVSVIQWPPFLLASKIPIALDMAKDFKGKDDEELFKKMKNIEYMCTAIVECYEALRDVIHYLLEDEADKMIVKQICTEIDESLEKKTFLTNFRMSGLLSLSERLEKFLTLLLSEDEEEENFQPQIINVLQDIMEIITQDVMVNGHEILESVHPSINIQNVKKEQRFEKINMDLRHDKPWKEKVVRLHLLLTVKESAINVPQNLEARRRITFFANSLFMKIPRAPEVRDMFSFSVLTPYYKEEVLYTDEELTRENEDGISILFYLQKIYPDEWTNFKERISDIKTYPDKDKQELTRQWVSYRGQTLYRTVRGMMYYRQALELQCFLESAGDAAMSGGYQNMVLSQKDQKPFLDRAQALVTYVVSCQVYGAQKKSNDQKDQSCYSNILKLMLLYPSLRVAYIDTREDTVNGRTQKVYYSVLLKGGDKLDEEIYRIRLPGPPTEIGEGKPENQNHAIVFTRGEALQTIDMNQDNYFEEAYKMRNVLQEFQLHRRGARKPTIVGLREHIFTGSVSSLAWFMSNQETSFVTIGQRILANPLRVRFHYGHPDIFDRLFHITRGGISKASKIINLSEDIFAGYNSTLRGGFITHHEYMQVGKGRDVGMNQISLFEAKVANGNGEQTLSRDVYRLGRRFDFYRMLSFYFTTVGFYFSSMVTVLTVYMFLYGRLYMVMSGVEMEILENPTIRQTKALEEALATQSVFQLGLLLVLPMVMEIGLEKGFRTAIGDFIIMQLQLASVFFTFQLGTKAHYFGRTILHGGSKYRATGRGFVVFHAKFADNYRLYSRSHFVKGLELFILLIVYEVYGESYRSSSLYMFITFSMWFLVASWLFAPFVFNPSGFDWQKTVDDWTDWKRWMGNRGGIGISPDKSWESWWDEEQEHLKHTNIRGRGLEIFLAFRFFIYQYGIVYHLDISHNIKTLLVYGLSWLVMIATLLVLKMVSMGRRRFDSDFQLMFRILKALLFLGFMSVMTVLFVVCGLTVSDLFAAILAFLPTGWAIVLIGQACRGLLRRVKLWDSVKELARAYEYIMGLIIFMPTAILSWFPFVSEFQTRLLFNQAFSRGLQISMILTGRKDKINSVDKPSTA

**>UdCalS8**

MSLFPLSFSTQILAMSREIVVADPIFYDCDGSDLFAGPSSDPISEPFESERLPQTLASDIQKFLRVANLVEIEEPRVAFLCRVHAFEIAHNSDKNSTGRGVRQFKTSLLQRLEQDEEITLRRRKEKSDVRELKRVYHEYKEFIVKHGKTFALENSHREKLINACSIASVLFEVLKRITSAANPHAIATRESASAKPDFFVPYNVLPLDHGGIQQAITQIPEIKVAIAAVRDTRGIPSAEDLEKHGPFIDLLDFLKYCFGFQEGNVANQREHLILLLANILIRRNHKQVSISKLEDVVLDDLMRKFFKNYSNWCKFLRKKSNIRLPYAKNEAQQYKLLYIGLYLLIWGEAANLRFMPECLCYIFHHMAYELRGMLIGDVNPTTWEKVIPAYGGSSESFLKNVVTPIYNVIREEAKKSNNGTTDHSCWRNYDDLNEYFWSPNCFEIGWPMCEDHNFFCVDSTTKPKKVKKATRASSKPSSPEEEMMNEEERDEEQGSPEERSHEQEREKDVTEKEWLGKTNFVEVRSFWQIFRSFDRMWIFFIVSLQAMIIMACYQLESPIQLFDKNIFEDVLSIFITCSILNLIKAILDMSFTWKARKTMAYAGKRKLMLKLVIAAIWTIVLPTCYAHSKSQYTCFTSQYSQSWLRKLCLSPYLVAVGIYLIPNAIEMVLFFVPVVRKYIETSNSKIFTLFSWTQPRLYVGRGMQETQVSVLKYMLFWMLVLLSKFCFSYWFEIKPLIQPTKRIMAIGIKNYDWHELFPKFKSNAGAIVAIWSPIIVVYFMDIQIWYSVFCAIFGGLYGILHHLGEIRTLGMLRSRFHSLPSAFNALLIPPSSRRTQRQVPGFFRKLAQGPQNEKNGVAKFVLVWNQVIKSIRSEDLISNREVDLMTIPMSSDLFSGTVRWPVFLIGNKFSTALSIAKDFKGTDETLFRKIRKDESMYYAVKECYESLKYILEVLIIRDLEKRIISTIINEVDESIAKLSLLEDFKLTELPNLRAKFVELLELLVEGNEDHKHKVVRALQDIFEIITKDTMINGSRILEMLSCSQQMENETAYFCRIVEPQIFEADKGESSIHFPFPDSAPLKEQIKRLLLLLTVKDTALDTPKNIDARRRISFFATSLFMTMPTAPKVCNMLSFSVLTPHFVEDVNYSMKELLSSQREVSIIFYMQKIFPDEWDNFLERMGCSNIDALKEEGREEDLRNWASFRGQTLSRTVRGMMYYREALKLQAFLDMAEDEDILEGYDTVERDNHALSAQLDALSDLKFTYVVSCQRFGSQKAAGDPRAQDIIDMMLKYPAFRLAYVEEKEVIVENRPKKVYSSVLLKGVNGFDQEIYRIKLPGPPEIGEGKPENQNHAIIFTRGEALQTIDMNQDSYLEEAFKMRNLLQEFLRCHGRRTPSILGLREHIFTGSVSSLAWFMSYQETSFVTIGQRLLAKPLRVRFHYGHPDVFDRIFHITRGGISKASKTINLSEDVFAGFNSTLRRGCITYHEYIQVGKGRDVGLNQISKFEAKVANGNSEQTISRDIHRLASQFDFFRMLSCYFTTIGFYFSSLISIIGIYIFLYGQLYLVLSGLEKALLVEAKLQNIESLETALASQSFIQLGLLTGLPMVMEIGLEKGFLVAVKDFVLMQLQLAAVFFTFALGTKTHHYGRTILHGGAKYRPTGRKVVVFHTSFAENFRLYSRSHFVKGFELMLLLVVYDLFRRSYESNVAYVLITYSIWFMSITWLSAPFLFNPSGFSWEKIVDDWKGWNKWIRQQGGIGIQQDKSWESWWNDEQCHLRHTGTVSRLFEIFLSLRFFMYQYGLVYHLDISQQSKNFLVYVLSWVVILLVFLIAKTVNIGRQKLSANYQLLFRLFKAILFVTVLSIIVILSKVCQLSSMDMIVCSLAFIPTGWGLIMIAQVVRPKIEETGFWEFTRVLAKAYDYGMGVALFAPIAVLAWLPIISAFQTRFLFNEAFNRHLQIQGILEGKRKQI

**>UdCals9**

MSNAEDLWERLVRAVLTRDRAGKDALGRPIGGIAAFVPSSLANNRDIDEILRVADEIQDVDPNVSRILCEHAYSLAQNLDPNSEGRGVLQFKTGLMSVIKQKLAKREGGVIDRSQDIARLLEFYKLYREKNDVDKLREEEMKLRESGTFSGNLGELERKTVKRKRVFATLKVLGMVLEQLSQEIPEELKRVMESDAAMTDDLIAYNIIPLDAPSITNTIVTFPEVRAAVSALKYFRGLPKLPADFPVPETRKADVLDFLHFIFGFQKDNVSNQREHIVHLVANDQSRLRVLDDDEPELDEAAVHNVFLKSLDNYIKWCNYLCIQPVWSNFDVLSREKKVLFISLYFLIWGEASNLRFLPECLCYIFHHMAREMDEILKQQSAQPAKSCDSENGVSFLDQVVFPLYEVIAAEAGNNENGRAPHSAWRNYDDFNEYFWSLHCFDLGWPWRKNSAFFQKPKPRKNILKAGGGSKHRGKTSFVEHRTFLHLYHSFHRLWIFLAMMFQALIIIAFNDGRFDRKTLREVLSLGPTFLIMKFLESVLDVMMMYGAYSTTRRLAVARIFLRFLWFGAASVVITFLYVKALLEEGQRNGNPAILKLYIIVIGIYAGIQFFLSVLMRIPFCHQLTNKCDRWPVIRFVKWMRQERHYVGRGMYERTTDFIKYMLFWVLVLSGKFAFAYFLQIQPLVSPTQKVIKMNSITYSWHDIVSKGNHNALTIVSLWSPVVCIYLLDIYVFYTLVSSVCGFLLGARDRLGEIRSLESLQKVFEEFPEAFMNALHVPLPNRNSNQVSGESLEKNKVDAARFSPFWNEIIKNLREEDYLTNHEMELLVMPRNSATLPLVQWPLFLLASKIIQAKDIAVENRDSQEELWERVSRDEYMKYAVQESYHSIKLILCEVLEGEGRMWVDRIYEDIEKSIKDRSFHVNFKLSNLPLVISRVTAILGILKEATEQEKGAVKAIQDLYDVVHHDVLSINMKENYETWSLLIRARNEGRLFTKINWPKGSESKSQIKRLYSLLTIKDSAANVPRNLEARRRLQFFTNSLFMEMPVAKPVNEMLSFSVFTPYYSETVLYSMPELLKRNEDGISILFYLQKIYPDEWKNFLARIGRHENTQESELGDSPNDILELRFWASYRGQTLARTVRGMMYYRKALMLQTYLEKISTGDLEAANSSNEAAETQGFELSPQARAQADLKFTYVVTCQIYGKQKEEQKPEAADIALLMQRNEALRVAFIDEIETLTDGKVQREFFSKLVKGDINGKDKEIYSIKLPGNPKLGEGKPENQNHAIVFTRGNAIQTIDMNQDNYFEEALKMRNLLEEFHCDHGIRPATILGVREHVFTGSVSSLASFMSNQETSFVTLGQRVLANPLKVRMHYGHPDVFDRVFHVTRGGISKASRVINISEDIFAGFNSTLRQGNITHHEYIQVGKGRDVGLNQIAIFEGKVASGNGEQVLSRDVYRLGQQFDFFRMMSFYFTTVGFYCCTMLTVLTVYVFLYGKTYLALSGVGETIQIRARIMDNTALSTALNTQFLFQIGIFTAVPMILGFILEQGFLRAVVSFVTMQFQLCSVFFTFSLGTRTHYFGRTILHGGARYQATGRGFVVKHIKFSENYRLYSRSHFVKGLEVVLLLIIFLAYGYNESGALGYILLSISSWFMALSWLFAPYLFNPAGFEWQKVVEDFREWTNWLLYRGGIGVKGAESWEAWWDEELSHIRTLEGRIAETILSLRFFIFQYGVVYKLSIQGSNTSLAVYGFSWIAFAVLILLFKVFTFSQKISVNFQLVLRFIQGVSFMVAIAGITVAIILTDLSISDIFACILAFVPTGWGILSIASAWKPVMKKLGLWKSIRSIARLYDAGMGMLIFIPIAFLSWFPFVSTFQTRLMFNQAFSRGLEISLILAGNNPNTGL

**>UdCals10**

MARVKENWERLVRATLNREQLRATGGHERTPSGIAGAVPQSLGKKTNIDAILQAADEIQSEDPTISRILCEQAYTMAQNLDPNSYGRGVLQFKTGLMSVIKQKLAKRDGGQIDRNRDIELLWQFYERYKRRNRVEDMQREQQRLRESGTFSTNIGDLESKSLHMKKVVATLRALVEVMEALSKDADPDGVGRLIKDELRKVKSSEAQLSGDITPYNIVPLEAPSLTNAIGVFPEVRGATSAIRYTEHFPQLPADFEIYGERDPDMFDLLEYVFGFQRDNIRNQREHVVLTVANAQSRFGIHVQSDPKIDEKAINEVFLKVLDNYTKWCRYLRMRPVWNSFEAINRDRKLFLVSLYLLIWGEAANVRFLPECICYIFHSMAKELDAVLDHGEAYRAASCKTETGSVSYLEKVIKPIYATLEAEAARNNNGKAAHSAWRNYDDFNEYFWSPACFELSWPMKLESPFLRKPPKKGKRTGKSTFVEHRTFLHLYRSFHRMWIFLALMFQTLAIIAFRDERLNLDTFKIVLSVGPTYAIMHFFESLLDVVLMYGAYTSARGMAISRQVIRFFWFGLSSAGVVYLYLKVLDERNSSNSFYFRIYVLVLGVYAAWRIFLGLILKFPACHKLSEMSDQSFFQFFKWIYQERYYVGRGLYESLSDYCRYVLFWLVIFAAKFSFAYFLQIRPLVEPTNIIRNLPQVQYSWHDFISKNNNHLLTIVSLWAPVVAIYLMDIYIWYTLLSAVIGGIMGARARLGEIRSIEMVHKRFVNFPEAFVRNLVSSETKRLPFNRQSSQEAQDMNKTYAANFSPFWNEIIKSLREEDYISNREMDLLSCPSNTGSLRLVQWPLFLLSSKILLAVDLALDCKDTQADLWNRICRDEYMAYAVQECYYSIEKLLYSLIDGEGRLWVERIYREINNSIMENSLVITLHLKKLPLVLSRFTALTGLLLRNEDPGLAKGAAKALFELYEVVTHDLLSSNLREQLDTWQILARARNEGRLFSRIAWPKDTETKELVKRLHLLLSVKDPATNIPKNLEARRRLEFFTNSLFMEMPAAKAVSEMISFCVFTPYYSETVIYSSSELHKENEDGISVIFYLQKIFPDEWKNFLERIGRPESTDETDLQKNPSDALELRFWVSYRGQTLARTVRGMMYYRRALMLQSYMEGRSLGVDGNSPSSIPTGQGFELSRESRAQADLKFTYVVSCQIYGQQKQRKAPEAADISLLLQRNEALRVAFIHVEETQDSDGKISREFFSKLVKADMHGKDQEIFSIKLPGDPKLGEGKPENQNHAIVFTRGEAVQTIDMNQDNYLEEAMKMRNLLEEFHTKHGLRPPTILGVREHVFTGSVSSLAWFMSNQETSFVTLGQRVLAYPLKVRMHYGHPDVFDRIFHITRGGISKASRVINISEDIYAGFNSTLRQGNITHHEYIQVGKGRDVGLNQIALFEGKVAGGNGEQVLSRDIYRIGQLFDFFRMLSFYYTTVGFYVCTMMTVLTIYIFLYGRVYLAFSGLDEGIGKSARRLGSTALDAALNAQFLVQIGVFTAVPMIMGFILELGLLKAVFSFITMQLQLCSVFFTFSLGTRTHYFGRTILHGGAKYRATGRGFVVRHIKFAENYRLYSRSHFVKALEVALLLIVYIAYGYTNNGPVSFILLTLSSWFLVISWLFAPYIFNPSGFEWQKTVEDFDDWTSWLLYKGGVGVKGDDSWESWWDEEQSHIQTLRGRILETILSLRFFMFQYGIVYKLHLTGNERSLAIYGFSWLILAGIVMVFKIFTYSPKKSAGFQLVIRFTQGVTSLMLIAAIILVVVFTNLSIPDLFASILGFIPTGWAIICLGITWKKPLRTLGLWDSVREFGRMYDAGMGMLIFAPIAFLSWFPFISTFQSRLLFNQAFSRGLEISIILAGNKANVDV

**>UdCals11**

MNFDNFGVTNNRPAMMPSRQPTRSSNHAPPARPLPPLNPYNIIPINDVLADHPSLRYTEVRAAAAALRTAGDLPRPPFVEWDPSYDLLDWLGVSFGFQRDSVRNQREHLVLHLANTQMRLPSPPSSPDSLDPAVLRRFRRKLLQNYTQWCSFLGRKSNVVLAARRGGDAVAVRREILYASLFLLVWGEAGNLRFVPECICYIYHHMAMELNNVLDERIDRDTGRPFTPTVSGECGFLKSVVIPIYQTISAEAEGSRGGKAPHSAWRNYDDLNEFFWSRRCFASLRWPLRLESKFFATTPKRDRIGKTGFVEQRSFWNLFRSFDRLWVMLVLFLQAAAITAWDDSSAPPWRSLQSRDSQVALLSVFVTWAGLRLLQAVLDAGTQYSLVTRRTKWVAVRMVLKAVAALGWTVAFSVLYARIWTQRQSDRGRRRGWSDEANARVLMFLKAAAAFLSPEALALALFIVPWVRNSVEELDFRPVSWLTWWFYTPIFVGRGLREGLVDNVKYSAFWVAVLSAKFTFSYFLQIRPLVGPTRALLELRGNYKWHEFFNSTNEIAVVLLWLPVVLIYLMDLQIWYAIFSSVVGAVIGLLSHLGEIRNMGQLRLRFQFFASALQFNLMPEEQLLRSDLTVAKKLRDAIHRLKLRYGLGQAYKKIESSQVEATRFALIWNEIMISFREEDIISDAEQELLELPPNCWNIRVIRWPCFLLGNELLLALSQAAELAELGHKSDWSIWFKICKNEYRRCAITEVYDSVKHLMFVIVRYGTEEYLILERFFSHVDDSVQRGKFAAEHDLRLLGRVHTKLIALLELLNNRKREMGAAVNLLQALYELCVRELPVEKKSMRRLREEGLASLSSDESGLLFVNAIDFPSFEDADFYRQLRRIRTILTSRDSMHDVPKNIEARRRIAFFSNSLFMNMPRAPFVEKMVAFSVLTPYYDEEVVFKTEALKRENEDGISTLFYLQKIYEDEWANFMERMRRDGLEDEAEIWEKKKGDLRLWASYRGQTLSRTVRGMMYYYRALKMLAFLDGASEMDIREGSQKIMAQGSSRRNKTLYGVQPEKSPPPPPGRTLNRAVTGVSLLFKGHEYGCALMKFTYVVACQLYGQHKGKGDPRAEEILQLMKSNEALRIAYVDEVDLGRDEVEYYSVLVKYDQELETEVEVYRVRLPGPLKIGEGKPENQNHAIIFTRGDALQTIDMNQDNYFEEALKMRNLLEEFKSNHGIRKPTILGVRENIFTGSVSSLAWFMSAQETSFVTLGQRVLANPLKVRMHYGHPDVFDRFWFLSRGGISKASKVINISEDIFAGFNCTLRGGNVTHHEYIQVGKGRDVGLNQISMFEAKVASGNGEQVLSRDVYRLGHRLDFFRMLSFFYTTVGFYFNTMMVVLTVYTFLWGRLYLCLSGVEQVAKDSKEDTSNNKALGAVLNQQFVIQLGLFTALPMIVENSLEHGFLPAIWDFLTMQAQLASLFYTFSMGTRTHFFGRTILHGGAKYRATGRGFVVQHKSFTENYRLYARSHFVKAIELGVILTVYATHSPLSANTFVYIVLTISSWFLVVSWIMSPFVFNPSGFDWLKTVDDYESFMGWLWYNGGIFTTSEQSWERWWYEEQDHLRTTGLWGKLLEIVLDLRFFFFQYGIVYQLGIAGESTSIGVYLVSWVYVVVAVGIYMVMSYAGDKYAENEHIYYRLVQLLVIVVLVLLAVLLLEFTPLKILDIVTSLLAFIPTGWGVILIAQVLRPFLQSTVVWDTVVSLARLYDMLFGIVVMAPMGLLSWLPGFQAMQTRILFNEAFSRGLQISRLLSGKKNN

**>UdCals12**

MSNRRARPPAPAASQPPDPDSETYNIIPVHNLLADHPSLRYPEVRAAAAALRSVGDLRRPPFARWEASMDLLDWLALFFGFQSDNVKNQREHLVLHLANAQMRLTPPPDNIDSLDSTVLRRFRKKLLRNYSDWCYYLGKKSNIWISDRREASSDGRRELLYVSLYLLVWGESANLRFVPECICYIFHHMAMELNKILEDYIDENTGQPVTPSVSGENAFLNCVVRPVYDTIRAEVESSRNGTAPHSVWRNYDDVNEYFWSKRCFEKLKWPIDIGSNFFVTSSRSRHVGKTGFVEQRSFWNLFRSFDRLWIMLALFLQAGIIVAWEGKQFPWQALKERDVQVRVLTVFITWSGMRFFQSLLDVGMQYSLVSRETKALGVRMVLKSVVAAAWIVVFGVFYAQIWTRRNQDTTPNWSRGSEGKIVTFLEVALVFVLPEILALALFIIPWIRNFVEGSNWRIFGMLSWWFQSRIFVGRGLREGLVDNIKYTLFWVVVLATKFTFSYFMQIKPMVQPTVLLLRAGKLHYEWHEFFQSSNRFAVGLLWLPVVLIYLMDIQIWYAIYSSFVGAAVGLFAHLGEIRNIQQLRLRFQFFASAIQFNLMPEEQLLNARGTLRNKFNDAISRLKLRYGLGQPYKKLESNQVEANKFALIWNEIIMIFREEDIISDRELELLELPQNSWNVRVIRWPCFLLCNELLLALSQATELVDASDKWVWHKICKNEYRRCAVIEAYDCVKYLVLSIIKRSTEEHSILTVMFQEIDHALQIERFTKTFKTTALPKLHAKLIRLVELLNKPNKDVGQVVNTLQALYEIVIRDFLRDKKGTEQLKEEGLAPQNPSSTTGHLFENAVQLPSPEDEAFYRQIRRLYTILHSRDSLQNIPINLEARRRIAFFSNSLFMNMPHAPQVEKMMAFSVLTPYYTEDVMYNREQLKKKNEDGISTLYYLQTIYDDEWKNFVERLRREGMVDEEEIWTTRLKELRLWASYRGQTLARTVRGMMYYYRALKMLAFLDSASEMDIREGARELGSMRRDDGLERMGSERTGSSKTLSRTNSSVSMLYKGHEYGTALMKYTYVVACQIYGAQKAEKKPQAEDILYLMKTNEALRVAYVDEVSTGRDEKDYYSVLVKYDQKLDREVEIYRVKLPGPLKIGEGKPENQNHAIIFTRGDAVQTIDMNQDNYFEEALKIRNLLEEFRRYYGARKPTILGVREHIFTGSVSSLAWFMSAQETSFVTLGQRVLANPLKVRMHYGHPDVFDRFWFMTRGGLSKASRVINISEDIFAGFNCTLRGGNVTHHEYIQVGKGRDVGLNQVSMFEAKVASGNGEQVLSRDVYRLGHRLDFFRMLSFFYTTVGFFFNTMMVILTVYAFLWGRLYLALSGIEGSIMSSNSNKALGTILNQQFIIQLGLFTALPMIVENSLEHGFMEAIWDFLTMQLQLSSVFYTFSMGTRTHFFGRTILHGGAKYRATGRGFVVQHKSFAENYRLYARSHFVKAVELGLILIVYATHSAVAKSTFVYIALTISSWFLVASWIMTPFVFNPSGFDWLKTVEDFDDFMNWVWFRGSVFAKAEQSWEKWWYEEQEHLRTTGIWGKLLEIILDLRFFFFQYGVVYQLDIAGGSTSIAVYLLSWIYVFVAFAIYVVVAYARNKYEANDHIYYRLVQFLVISLGVLVIIALLEFSDFSMIDIFTSMLAFVPTGWGLLLIAQVFRPFLQRTILWDAVVSVARLYDILFGIIIMVPVALLSWLPGFQSMQTRILFNEAFSRGLRIFQIVTGKKSKTDS

**>CsaCalS12-like_XP_030482920.1**

MSHRHRPTVSDSPHRPGPPAQTELESEPYNIIPVNNLLADHPSLRFPEVRAAAAALRAVGNLRKPPFAQWLPQMDLLDWLALFFGFQKDNVRNQREHLVLHLANAQMRLTPPPDNIDTLDAAVLRRFRRKLLKNYTEWCYYLGKKSNIWISDRREAAADQRRELLYVSLYLLIWGESANLRFVPECLCYIFHNMAMELNKILEDYIDENTGQPVMPSISGENAFLNFVVTPIYETIKAEVESSRNGTAAHSVWRNYDDINEYFWSKRCFEKLKWPIDVGSNFFVTSTRKRHVGKTGFVEQRSFWNLYRSFDRLWIMLILFLQAAIIVAWEGTDYPWQALKHREVQVRILTVFFTWSGLRFFQSLLDAAMQYTLVSRETLGLGVRLVLKSIVAAGWIVVFGVFYGRIWTQRNQDRRWSPEANRRVVTFLEVVLVFILPELLALALFIIPWIRNFVEQTNWRIFRMLSWWFQSRIFVGRGLREGLVDNIKYTLFWVVVLATKFCFSYFMQIKPMVAPSKTLLDLKNLKYEWHQFFGSSNRFAVGLLWIPVVLIYLMDLQIWYSIYSSFVGAGVGLFAHLGEIRNIQQLRLRFQFFASAIQFNLMPEEQLLNARGTLRNKFNDAINRLKLRYGFGQPYRKLESNQVEANKFALIWNEIIMIFREEDIISDRELELLELPQNSWNVRVIRWPCFLLCNELLLALSQAKELVDASDKWLWYKICKNEYRRCAVIEAYDCLKHLVRAILKRNSEEHAIVTVLFQEIDQSVQSERFTKTFKTTALPLLHSKLIKLVELLNKPKKDPNQMVNTLQALYEIVVRDFFKEKRSTDQLREEGLAPQNPDSMAGLLFENAVQLPDHDDETFYRQVRRLHTILISRDSMQNIPVNLEARRRIAFFSNSLFMNMPHAPQVEKMMAFSVLTPYYTEEVVYSKEQLRTENEDGISILYYLQTIYHDDWKNFVERMRREGMVDDKELWTTKLRELRLWASNRGQTLSRTVRGMMYYYRALKMLAFLDSASEMDIREGARELGSLRRESFNSERSPSARSLSRTNSSVSLLFKGHEYGTALMKFTYVVACQIYGTQKARKDPNAEEILYLMKTNEALRVAYVDEVSTGRDGKEYYSVLVKFDQQLNKEVEIFRVKLPGPLKLGEGKPENQNHAMIFTRGDAVQTIDMNQDNYFEEALKIRNLLEEFRRYYGARKPTILGVREHVFTGSVSSLAWFMSAQETSFVTLGQRVLANPLKVRMHYGHPDVFDRFWFLTRGGISKASRVINISEDIFAGFNCTLRGGNVTHHEYIQVGKGRDVGLNQISMFEAKVASGNGEQVLSRDVYRLGHRLDFFRMLSFFYTTVGFFFNTMLVILTVYAFLWSRLYLALSGVEGSALAQDSNKALGTILNQQFIIQLGIFTALPMIVENSLEQGFLQAIWDFLTMQLQLSSVFYTFSMGTRTHFFGRTILHGGAKYRATGRGFVVQHKSFAENYRLYARSHFVKAVELGLILIVYATHSAVAKDTLVYIALTITSWFLVMSWILAPFVFNPSGFDWLKTVDDFDDFMNWIWFRGSVFAKAEQSWERWWYEEQEHLRTTGIWGKIMEIILDLRFFFFQYGIVYQLDITAGSTSIAVYGLSWIYVLVAFGIYVVVAYARDKYAAKEHIYYRLVQFLVIILAILVIIALLKFTNFNFIDIFTSMLAFVPTGWGLLLIAQVFRPLLQKTIFWNVVVSVARMYDILFGVTIMIPMAVLSWLPGFQSMQTRILFNEAFSRGLRIFQIVTGKKSKTDV

**>CsaCalS11-like_XP_030480901.1**

MTHYVPPTRHRVGGGGRDSYAPPPPPVPPSMREVYNIIPIHDLLLMDHPSMRYPEVRAAAAALKSTGGLPLHPYAAYDPNHDLMDWLGLFFGFQNDSVRNQREHLVLHLANSQMRLQPPPAVPDQLEQSVLRKYRKKLLQNYSSWCSYVGRKSNVSIGSRGGSGDCSRELLYVALYLLVWGEAGNLRFAPECICYIYHHMAMELNRVLSGKPDPNTGQLFLPSFSGDCAFLKSVIMPIYKTISDEVESSRNGKAPHSAWRNYDDVNEYFWSRRCFNKLKWPIDLGSNFFSTTPKSRRVGKTGFVEQRSFWNLFRSFDRLWILLILFFQAAVIVAWDGETFPWQGLERRDVQVELLTVFITWGALRLLQSVLDAGTQYSLVSRETKWLGLRMVLKSLVALAWTVVFSVLYARIWSQKNSDRRWSNGADQRIFTFLEAVLVFLAPELLALVLFIVPWVRNFVEELNWRVVSWLTWWFYTRIFVGRGLREGLFDNIKYTVFWVLVLASKFSFSYFLQIKPLISPTKALLNLDGKYRWHEFFSSTNEIAVVLLWLPVILIYMMDLQIWYAIFSSIYGAVIGLFSHLGEIRNIGQLRLRFQFFASAMQFNLMPEEQLLTSDMSLLKKARDSIHRLKLRYGLGQAFKKIESSQVETTRFALIWNEIVIAFREEDLISDYELELLELPPNCWNIRVIRWPCFLICNELLLALSQATELADETDLSLWLKICKSEYRRCAVIEAYDTVKHFLFVIVKYGTEEYLIVERFFNEIDSCIQRGKFTVEYKMSLLQKIHGKLISLIELLLNPKRDIEKAVNLLQALYELSVRELPKAKKSMEQLRREGLASINPANQTELLFEKAVVFPPCEDALFYRQLRRLHTILTSRDYIHNVPKNIEARRRIAFFSNSLFMNMPRAPIVEKMMAFSVLTPYYDEDVIFKLDGLLKDNEDGISTLFYLQKIYEDEWTNFMERMRREGLEHDEQIWEEKGRDLRLWASYRGQTLSRTVRGMMYYYRALQMLAFLDNASEMDIREGSQQIASHGSAKRNRGFEGMQPAKQPTSRNLGRAGSGVGLLFKGHEYGCALMKFTYVVACQLYGQHKAKGDPRAEDILYLMKNNEALRIAYVDEFNFGREDAEYYSVLVKYDQQLGREVEIYRIRLPGPLKLGEGKPENQNHAIIFTRGDALQTIDMNQDNYFEEALKMRNLLEEFKVSYGIRKPTILGVRENIFTGSVSSLAWFMSAQETSFVTLGQRVLANPLKVRMHYGHPDVFDRFWFLPRGGISKASKVINISEDIFAGFNCTLRGGNVTHHEYIQVGKGRDVGLNQISMFEAKVASGNGEQVLSRDVYRLGHRLDFFRMLSFYYSTVGYYFNTMMVILTVYTFLWGRLYLALSGVEDVASKNTSNNEALGAILNQQFVIQLGIFTALPMIVENSLEHGFLPAIWDFLTMQFQLASLFYTFSMGTRTHFFGRTILHGGAKYRATGRGFVVQHKSFVENYRLYARSHFVKAIELGVILTVYASHSPMADNTFVYIIMSLSSWFLVVSWIMSPFVFNPSGFDWLKTVEDFEIFMNWLWFTGGVFTTSDHSWERWWYEEQDHLKTTGLWGKLLEILLDLRFFFFQYGVVYQLGISGGNTSIAVYLLSWIYMVVAVGIYMIIAYARDKYAVRDHMYYRLVQLLVILVLVLVVVLLLEFTPFKFIDIVTSLLAFVPTGWGIILIAQVLRPFLQSTVVWETVVSVARMYDLLFGVIVMMPMALLSWLPGFQAMQTRILFNEAFSRGLQISRILTGKKTN

**>CsaCalS5_XP_030499529.1**

MSNLESGPQGLTRRPSRSAATTFSTEVFDNEVVPSSLASIVPILRVANEIENERPRVAYHCRFYAFEKAHRLDPSSSGRGVRQFKTLLLQRLERDNSSSLASRVKKTDAREIESFYQQYYEHYIRALDQGEQADRAQLAKAYQTAGVLFEVLCAVNKSEKVEEVAPEIIAAARDVQEKTEIYTPYNILPLDSAGASQSIMQLDEVKAAVAALWNTRGLNWPHAFEQHRQKAGDLDLLDWLRAMFGFQRDNVRNQREHLILLLADAHIRLQPKAEPLNKLDDRAVDIVMSKLFKNYKTWCKFLGRKHSLRLPQGQQEIQQRKILYMGLYFLIWGEAANVRFMPECLCYIFHNMAYELHGLLAGNVSIVTGENIKPSYGGDDEAFLRKVITPLYRVIEKESNKSGNGKAPHSGWCNYDDLNEYFWSSHCFSLGWPMRDDGDFFKSTRDLKQGRKGPRRKSGSTGKSYFVETRTFWHIFRSFDRLWTFYVLTLQAMIIIAWKGISPIDIFRKDVLYHVSSVFITAAFLRLLQSILDLFLNFPGYHRWKSTDVLRNLLKIIVSLAWVVILPLFYMHSFKGAPVQIRDMLSFLKQVNGVPPFYILAVAIYLIPNLLAAALFIFPMLRRWIENSDWHIIRFLLWWSQPRIYIGRGMHESQFALIKYTLFWVILLCSKLAFSYLAQIKPLVKPTKDIMSIHRVEYEWHEFFPKAQNNYGAVVSLWAPVIMVYFMDTQIWYSIYSTLYGGVIGAFDRLGEIRTLGMLRSRFQSLPGAFNTYLVPSDRSQKRGFSFAKRFAEITASRRSEAAKFAQLWNEVICSFREEDLISDREMDLLLVPYSSDPGLKIIQWPPFLLASKIPIALDMAAQFKSKDSDLWKRICADEYMKCAVIECYEFFKHVLSVLVVGENEKRIISIIIKEVESSISKNLLLANFRMGPLPTLCKKFVELVEILKDAEPSNKDTVVLLLQDMLEVVTRDMMVNELRELVEVGHSSKDTGRQLFAGTDTRPAIAFPPVVTAQWEEQIRRLYLLLTVKESATDVPTNLEACRRISFFTNSLFMDMPRAPKVRKMLSFSVMTPYYSEETVYSKTDLELENEDGVSILYYLQKIFPDEWNNFMERINCKKDSEIWETEENILQLRHWVSLRGQTLCRTIRGMMYYRRALKLQAFLDMANESEILGGYKAITVPSEEDKKSQRSLYAQLEAVADMKFTYIATCQNYGNQKRSGDRRATDILNLMVNNPSLRVAYIDEVEENEGGKVHKVYYSVLVKAVDNLDQEIYRIKLPGSAKIGEGKPENQNHAIIFTRGEALQTIDMNQDNYLEEALKMRNLLEEFNEDHGVRPPTILGVREHIFTGSVSSLAWFMSNQETSFVTIGQRVLARPLKVRFHYGHPDVFDRIFHITRGGISKASRGINLSEDIFAGFNSTLRRGNVTHHEYLQVGKGRDVGLNQISLFEAKVACGNGEQTLSRDIYRLGHRFDFFRMMSCYFTTVGFYVNAMLVVLTVYVYLYGKLYLSLSGLEQAIVKFARAKGNNALKAAMASQSVVQLGLLMALPMIMEIGLERGFRTALGDMIIMQLQLAAVFFTFSLGTKVHYYGRTILHGGAKYRATGRGFVVKHEKFAENYRMYSRSHFVKGLELMILLVAYHIYGSAAPDTATYTFLTFSMWFLVASFLFAPFLFNPMGFEWQKIVEDWDDWSKWISSRGGIGVPANKSWESWWDEEQEHLQHTGFLGRFWEIVLSLRFFLYQYGIVYHLNVARNETSIIVYGFSWLVIVAVMIILKIVSLGRKKFSADFQLMFRLLKFLLFIGFIVTLTILFTFLSLTVGDIFASLLAFMPTGWAILQISQSLRPLMKGLGFWGSVKALGRGYEYMMGLVIFAPVAILAWFPFVSEFQTRLLFNQAFSRGLQIQRILAGGKKDK

**>CsaCalS1-like_XP_030510672.1**

MAYNRRSDQPTQRLQRAQTARNVLEPMLDSEVVPSSLVDIAPILRVANEVEAKNPRVAYLCRFYAFEKAHRLDPKSSGRGVRQFKTALLQRLEKEDKTTLEGKVKSDAREMQSFYRKYYKEYIQALQQVDNADRTRLTRAYQTAKVLFEVLRAVNQTEAVPEEILEAHTKVEEKSQLYVPYNILPLDAEGQNHAIMNYPEIKAAASALRNTRGLPWPREHKKKDDEDILDWLMLMFGFQGDNVGNQREHLILLLANVHMRQWPKPEQYKIDDRALTEVMKKLFKNYKKWCKYLDRKSSLWLPSIQQDVQQRKLLYMGLYLLIWGEAANLRFMPECLCYIYHHMAFELYGVLNGTMSPMTGEPTKPAYGGEKEAFLKKVVTPIYNIIAQESRKSKGKSKHSQWRNYDDLNEYFWTVDCFKLGWPMRATSSFFCCPNDNKVDTHDRDKERSAPNVDRWIGKSNFVEIRSFYHIFRSFDRMWSFYILSLQAMIIVAWNGSGELSSIFEADVFNKVLSIFITAAILKLGQATLDVILSWKARKSMSSYVQLRYILKVISAAAWVVILPVTYAYGLKNPSGFAQTMKNWFGNGPGSSSVFIMAVVIYLSPNMLSLLLFLFPFIRRPLERSNYTILTVMMWWSQPRLYVGRGMHESLFSLFKYTMFWVLLLVSKLAFSYYLEIKPLVGPTKAIMEIHISTYQWHEFFPHANNNIGVVIALWAPILLVYFMDTQIWYAIFSTIFGGIYGAFRRLGEIRTLVMLRSRFRSIPGAFNDLLIPKDKSEHTKKKGIKAAFFRKDKEVLSKTREATKFAQLWNEIVNSFREEDLISNREMDLLLVPYMADPDLKLIQWPPFLLASKIPIALDMAKDSSGQRSQDNELKKRINKDEYMLCAVEECYSSFQSIIRFLVLGHRETTVIDNIFSSIDEHIKKEDLISELNMSALPDLYEQFVILIKYLLDNKAEDKDEVSIILLNMLEIVTRDILEDESPSLLESSHGGGLYEKDEGMMPLDQQYKFFSELRFPVTSETDAWKEKIRRIHLLLTEKESAMDVPSNLEARRRISFFSNSLFMEMPPAPKVRNMISFSVLTPYYTEEVTFSVSQLEEQNEDGVSILFYLQKIFPDEWTNFLERVNCLSEEELMSTVELQEKLCLWASYRGQTLTKTVRGMMYYRKAMELQAFLDMAKDSDLMKGYKAAESDNAELGERSLMVQCQAVADMKFAYVVSCQQYGIDKRAGERRANDILKLMRTYPSLRVAYIDEVEEPVSEDKSVEASKEKSIGASKNKFKPEVQKVYYSKLVKVPAKRSDSSELVQNLDQLIYQIKLPGPAMLGEGKPENQNHAIIFTRGEALQTIDMNQDNYLEEAFKMRNLLQELLEKHGDVRTPTILGLREHIFTGSVSSLAWFMSNQENSFVTIGQRLLANPLKVRFHYGHPDVFDRLFHLTRGGVSKASKIINLSEDIFAGFNSTLREGNVTHHEYIQVGKGRDVGLNQISVFEAKIANGNGEQTISRDIYRLGHRFDFFRMLSCYFTTVGFYFSTLLSVLTVYVFLYGRLYLVLSGLEEGLRTQPAIRDNKSLQVALASQSFVQIGLLMALPMVMEIGLERGFRNALTDFILMQLQLAPVFFTFSLGTKTHYYGRTLLHGGAEYRGTGRGFVVFHAKFAENYRFYSRSHFVKGIELMILLLVYHIFGYSYTGVVAYVLITVSMWFMVGTWLFAPFLFNPSGFEWQKIVDDWNDWNKWISNQGGIGVSPDKSWESWWQKEQQHLRHSGMRGAIAEILLALRFFVYQYGLVYHLSITKKTKSFLVYGISLLVIIAVLLLTKVASIGRRRFSAQFQLLFRLFKGFVFLSFLTLFITLIALPHMTLQDIVAIILAFMPTGWGLLLIAQACKPLIENTALWASVRTLARYYEVIMGMILFIPVAFLAWFPFVSEFQTRMLFNQAFSRGLQISRILGGQRKDRSSRIKK

**>CsaCalS10_XP_030494101.1** MNRRVQDDWERLVRATLKREQLRAAGQGHERAPSGIAGSVPASLVKTTNIDAILQAADEIQSEDPTVARIMCEQAYSMAHSLDPDSDGRGVLQFKTGLMSVIKQKLAKRDGAPIDRNRDIEHLWNFYQRYKKRHRVDDIRKEEQRMRESGTFSTDFGKMEMKSLEMKKIVATLRALVEVMEALSKEADPRGVGGLIKEELRRIKSSEATLSGEFTPYNIVPLEAPSLTNAIGIFPEVRGAISAIRYTEHFPRLPDDYVIVGDRDADMFDLLEIVFGFQKDNIRNQRENVVLSIANAQSRLGIPTEADPKIDEKAINEVFLKVLDNYIKWCKYLRIRIAWNSLEAINRDRKLFLVSLYFLLWGEAANVRFLPECICYIFHNMAKELDAVLDHGEAYPADSCKTETGSVSFLDKIICPIYKIMSLEAERNNNGKAAHSAWRNYDDFNEYFWSPSCFELSWPMKRESRFFLEPKKKGIRTGKSSFVEHRTFLHLYRSFHRLWIFLALMFQTLAIIAFNDGRLNMDTFKSVLSIGPTFAIMNFVESCLDVILMFGAYTSARGMAISRLVIRFFWFGLCSAFVTYVYLKVLEERHSRNSNNSIYFRIYILVLGVYAGLRVVLGLLLKFPACHSLSAMSDQSFFQFFKWIYQERYYVGRGLYESLSDYCRYVLFWLVVFTCKFCFAYFLQIQPLVSPTNTIAGLRNLDYSWHDLVSKGNDNALTIACLWGPVVAIYLMDIHIWYTLLSAVIGGVMGARARLGEIRSIEMVHKRFESFPEAFVKNLVSPQTKRLPFNRQSSQDAQDTDKTDAAIFSPFWNEIIKSLREEDYISNREMDLLSCPSNTGSLGLVQWPLFLLSSKILLAIDLALDCKDTQRDLWNRICRDEYMAYAVRECYYSIEKLLYSLIDGEGRLWVERIYREINNSILEGSLVITLSLKKLPVVLKKFTALTGLLLRNEDPDLSKGAAKAVYELYEVVTHELLSPDLREQLDTWNILARARNEGRLFSRIEWPKDTETKELVKRLHLLLTVKDSAANIPKNLEARRRLEFFTNSLFMDMPSAKPVSEMVPFCVFTPYYSETVLYSSSELQKENEDGISILFYLQKIFPDEWKNFLERIGRADSTADSELQKSSSDALELRFWVSYRGQTLARTVRGMMYYRRALMLQSYLEKRSLGVDGYSQGSIPTSQGFELSRESRAQADIKFTYVVSCQIYGQQKQRKAPEAADISLLLQRNEALRVAFIHVEDGGADGKGPKEFYSKLVKADIHGKDQEIYSIKLPGDPKLGEGKPENQNHAIVFTRGEAVQTIDMNQDNYLEEAMKMRNLLEEFHRNHGLRPPTILGVREHVFTGSVSSLAWFMSNQETSFVTLGQRVLASPLKVRMHYGHPDVFDRVFHITRGGISKASRVINISEDIFAGFNSTLRQGNITHHEYIQVGKGRDVGLNQIALFEGKVAGGNGEQVLSRDVYRLGQLFDFFRMCSYFFTTVGFYVCTMMTVLTVYIFLYGRVYLAFSGLDEQIAKQAKKLGSTALDAALNAQFLVQIGIFTAVPMIMGFILELGLLKAVFSFITMQLQLCSVFFTFSLGTRTHYFGRTILHGGAKYRATGRGFVVRHIKFADNYRLYSRSHFVKALEVALLLIVYIAYGYTAGGAVSFVLLTISSWFLVISWLFAPYIFNPSGFEWQKTVEDFDDWTSWLLYKGGVGVKGDDSWESWWEEELAHIQTLRGRILETILSLRFFMFQYGIVYKLHLTAKERSLAIYGFSWVVLLVIVMVFKVFTYSPKKSANFQLVLRFTQGVTSLGLIAALALVVIFTDLSIPDLFASILAFIPTGWAIICLAVTWKRVVWSLGLWDSVREFARMYDAAMGMLIFAPIAFLSWFPFISTFQSRLLFNQAFSRGLEISIILAGNKANVDV

**>CsaCalS7_XP_030485184.1**

MASSSGTKNDVAPPRTLSRGMTRMPTRYVELGNEDNAAVDSELVPSSLASIAPILRVANEIQNENPRVAYLCRFHAFEKAHMMDPTSSGRGVRQFKTYLLHRLEKEEEEAVHILARSDPKEILLYYQQFYERNIKEGEYTKKPEEMAKICQIATVLYEVLKTVVPPHQIDAQTKKISEDVDSKRDQYAHYNILPLYAVGVKPAIMELPEIKAALRALSNVGSLPKPRIQRTPTGPDDNVPTERVQPVNDILDWLSAIFGFQKGNVANQREHLILLLANIDVRRRSSERHTELAAATVQNLLDKFFKNYRSWCKYLHCKSNLRFPQVTDKQQLELIYIALYLLIWGEASNIRFMPECLCYIFHNMANEVYGILYSNASDVIGDTYQNEACHEEHFLTHVITPIFDVLQKEAKRNNNGKASHSNWRNYDDLNEYFWSKKCFSLGWPMNLESDFFRHSDGQPSNKRVNQAVSGAKKPKTNFVEVRTFLHLYRNFDRMWVFFILAFQAMLIVAWSPSGSIVAFFDADVFRSVLSIFITSAFLNFLQATLDIVLSFNAWRSLKITQVVRYLLKFVVAAFWVVVLPICYSSSVQSPTGLVKFFRSWAGDWSNHSLYDYAIAIYLLPNILAAVLFVLPPLRRTMERSNMHITTLLMWWAQPKLYVGRGMHEDMFSLLKYTLFWIMLLISKLAFSYYVEIAPLVEPTKLIMEMPIDNYQWHEFFPHVPHNIFIIIAIWTPIILVYFMDAQIWYAIFSTLFGGIHGAFSHLGEIRTLGMLRSRFESVPLAFSRRLMPSTDTDHSKQKNLDPSQVRKNIANFSQVWNEFIYSMREEDLISNRDRDLLLVPYCSNEVSVVQWPPFLLASKIPIALDMAKDFKGKDDNELFKKIKSDDYMYSAVIECYESLRDVIYGLLEDKADKMIVESICKEVDECLDRKVFLSNFRMSGMPSLSERLEKFLGKLLSDEDDETLLPQLINVLQDIMEIITQDVMCNGHQILETAHHHSGHNVKKEQRFEKINLNRRYNSSWKEKVVRLNLLLTVKESAINVPQNLDARRRITFFANSLFMNMPRAPEVRDMLSFSVLTPYYKEDVLYTDDDLNKENEDGISILFYLQKIYPDEWQNFTARIKNIEKDKSDLIRQWVSYRGQTLYRTVRGMMYYRKALELQCFLELAGDNAIFSGYRTLELSEKDQKTFSDRAQALADLKFTYVVSCQLYGVQKKSNDPRDQSCYTSILKLMLTHQSLRVAYIDTREDTVNGRPQKVFYSVLLKGGDKLDEEIYRIKLPGPPTVIGEGKPENQNHAIIFTRGEALQTIDMNQDNYYEEAFKMRNVLAEFIKPRLGDRKPTILGLREHIFTGSVSSLAWFMSNQETSFVTIGQRILANPLRVRFHYGHPDIFDRIFHLTRGGISKASKTINLSEDIFAGYNSTLRGGFITHHEYIQVGKGRDVGMNQISNFEAKVANGNGEQTLSRDVYRLGRRFDFYRMLSFYFTTVGFYFSSMVTVLTVYVFLYGRLYMVMSGVEREILENPTIHQTKALEEALATQSVFQLGLLLVLPMVMEIGLEKGFRTALGDFIIMQLQLASVFFTFQLGTKAHYFGRTILHGGSKYRATGRGFVVFHAKFADNYRLYSRSHFVKGLELFILLIVYEVYGESYRSSNLYLFITFSMWFLVASWLFAPFVFNPSGFDWQKTVDDWTDWKRWMGNRGGIGISPDKSWESWWDEEQEHLKHTDFRGRVLEILLAFRFFIYQYGIVYHLDISHHSKSLLVYGLSWLVMVTCLLVLKMVSMGRRRFGTDFQLMFRILKALLFLGFMSVMTVLFVVCGLTISDLFAAILAFLPTGWAIVLIGQACRGLLKRIGLWDSIKELARAYEYIMGLIIFMPTAILSWFPFVSEFQTRLLFNQAFSRGLQISMILAGRKDKTETKEFSSV

**>CsaCalS9_XP_030504614.1**

MSQVDELWERLVRAVLKRERTGKDAYGQPVDGIAGNVPSSLANNGDIDEILRAADEIQNENPNVSRILCEHAYSLAQNLDPNSEGRGVLQFKTGLMSVIKQKLAKREGGVIDRSQDIALLQEFYRVYREQNDVEKLREEELSLREAGAFSGNLGELERRTVKRKRVFATLKVLGTVLQQLTEEIPDELRRVMETDAAMTDNLIAYNIIPLDAPSVTNRIGFFPEVKAAISVLKYRGLPKLPEDFPIPATRNADVFDFLHYVFGFQKDSVSNQRENIVHLLANEQSQLRVLEEAEPELDEAAVQNVFLKSLDNYIKWCNYLCIQPVWSNFDVVVKEKKILFVSLYFLIWGEAANVRFIPECLCYIFHHMARELDEILRHQIAQPANSCKAGDGVSFLDQVIFPLYDVMQAETKSNKDGKAPHSAWRNYDDFNEHFWSLHCFELGWPWRLGSPFFQKPELSKNMLKSGRSKHRGKTSFVEHRTFLHLYHSFHRLWIFLAMMFQGLAIIAFNDENFNGKTLREILSLAPTFVVMKFFESVLDVIMMFGAYSTSRRLAVARIFLRFIWFSTASVVVSLLYVKALQEESRPNGNGVIFRLYLIVIGIYGGIQFFISFMMRIPACHRLTNQCDRWPLIRFVKWMRQERYYVGRGMYERTTDFIKYLVFWLVILSAKFSFAYFLQIRPLVKPTRTVVKMDTIQYSWHDFVSQHNHNALTVVSLWAPVVAIYLLDIHVFYTIISSVWGFLLGARDRLGEIRSLELVHKLFEEFPEAFMTTLHVPLSNSTSQNASSQVFEKKVNAARFSPFWNEIIANLREEDYLTNQEMELLLMPKNTGTLNLVQWPLFLLASKIILAKDIAVDGRDSQDDELWDRISRDEYMKYAVQECFYTLKHILTTILQDEGKLWVERIYEDIQSSIGQRNLHSDLNLTKLPLLITRVTALMGMLKEVETSDQEKGAVKAVQDLYDVVRLDLLSVNMGENRETYYLVSKAWKEGRLFQKIKWPKDPELRSQVKRLYSLLTIKDSAANVPRNLEARRRLQFFTNSLFMEMPVAKPVNEMLSFSVFTPYYSEVVIYSMKELLERNEDGISVLFYLQKIFPDEWRNFLHRIGRDENAHESELDTPNDILELRLWASYRGQTLARTVRGMMYYRKALMLQTYLERKSSGDLETAISNTDEIETQGFTLSPEARAQADLKFTYVVTCQIYGKQKEDQKPEAADIALLMQRNEALRVAFIDEVETLKDDGKVQREYFSKLVKGDINGKDKEIYSIKLPGNPKLGEGKPENQNHAIVFTRGNAVQTIDMNQDNYFEEALKMRNLLEEFHRDHGIRPATILGVREHVFTGSVSSLASFMSNQETSFVTLGQRVLSTPLKVRMHYGHPDVFDRVFHITRGGISKASRVINISEDIFAGFNSTLRQGNITHHEYIQVGKGRDVGLNQIALFEGKVAGGNGEQVLSRDVYRLGQQFDFFRMMSFYFTTVGFYFCTMLTVLTVYVFLYGKAYLALSGVGETIQVRARIMDNTALTTAMNTQFLFQIGIFTAVPMILGFILEQGFLKAVVSFVTMQFQLCSVFFTFSLGTRTHYFGRTILHGGAKYHATGRGFVVKHIKFAENYRLYSRSHFVKGLEVVLLLVIYLAYGYNEGGIISYILLSVSSWFMALSWLFAPYLFNPSGFEWQKVVEDFRDWANWLLYRGGIGVKGEESWEAWWDEELAHIRTFSGRVAETILSLRFFIFQFGVVYKLDLQGNNTSLSIYGFSWIVLAVLVILFKVFTFSQKISVNFQLVLRFIQGISFLVALAGITVAIILTDLSVPDIFASILAFVPTGWGLLSIAAAWKPLMKKLGLWKSVRSIARLYDAGMGMIIFIPIAFFSWFPFVSTFQTRLMFNQAFSRGLEISLILAGNNPNSGI

**>CsaCalS8_XP_030490271.1**

MAHHEIVVADPIFYDCEQDEAGPSSSSITISSPIDDQNSVPEPFESERLPPTLATEIQRFLRVANLVEIEEPRIAFLCRVHAFEIAHNMDKNSSGRGVRQFKTSLLKRLESDEITTLMKRKETSDVRELRRVYREYKEYIYQNVRNSALEHSHREMLINAYTIASVLLEVLTRITSAANPQALANRERANKKADFFVPYNILPLDHGGIQQAIMQLNEIKAAIAAVRNVRGLPSAQDFQRHGEFIDLFDFLQYCFGFQEGNVANQREHLILLLANIHIRKASKQSVLKLEEVVVDELMRKFFKNYTNWCKFLPRKSNIRLPYVKQEAQQYKLLYIGLYLLIWGEAANLRFMPECLCYIFHHMASELHGMLTGAINPTTWEKVMPAYGGGPESFLNNVVTPIYTVIKKEAEKSKNGTTDHSIWRNYDDLNEYFWSPDCFQIGWPMRPDHNFFFTNSEPKDEKASGSSKAKEKQKREESQEEEEDNDEHEELGEKCTDKKDEQEWLGKSNFVEVRSFWQIFRSFDRMWSFFLLSFQAMIIMACYELESPLQLFDSGIFEDILSIFITSSILKLIQAILDISFTWKARQMMVYREKIRLKVKLVVAVIWTIVLPTCYAYSRRKYTCFSSQYGSWLEEWCLSSYLVAVGIYMIPNAIEMVLFFVPAVRKYIEVSNWRIFTLLAWTQPRLYVGRGMQESQVSVLKYTLFWILVLSSKFSFSYWFEIKPLIGPTKQIMKIGVKNYDWHELFPKVQNNAGAIVAVWAPIVVVYFMDTQIWYSVFCTIFGGLYGILHHLGEIRTLGMLRSRFHTLPSAFDACLIPPSSKNEKKGRKGFFQNRFHKESGVGKDGVAKFVLVWNQVINSIRSEDLISNREVDLMTIPMSSELFSGVVRWPVFLIANKFSTALSIARDFVGKDEILVRKIRRDKCMYYAVTECYETLKNILKFLIVRDLEKRVISEIISDVEESLKRSTLLEDFKMTELPNLRDKFVELLELLVEGNEDHAGKVVKVLQDIFEIITNDMMVDSARIMELLSSFQETDEEAPYFCRHIEPQLFEKYAGEMSIQFPLPDNAPMDEQIRRLILLLTVKDSALDVPTNLDARRRISFFSTSLFMNMPTAPRVQNMLSFSVLTPHYMEDVNFSRNELHSSQREVSIIFYMQKIFPDEWKNFLERMECASLEALKAEDKEEELRNWASFRGQTLSRTVRGMMYYREALKLQAFLDMAQDEDILGGYDTDNHVLSAQLDALADLKFTYVISCQQFGSQKSAGDPRAQDIIDLMKRNPALRVAYVEEKEVIVPDSKPQKIYSSILLKAVNGFDQEIYRIKLPGPPEIGEGKPENQNHAIIFTRGEALQTIDMNQDSYLEEAFKMRNLLQEFLRSQGRRPPTILGLREHIFTGSVSSLAWFMSYQETSFVTIGQRLLAKPLRVRFHYGHPDVFDRIFHITRGGISKASKTINLSEDVFAGFNSILRRGCITYHEYMQVGKGRDVGLNQISKFEAKVANGNSEQTLSRDIYRLGRQFDFFRMLSCYFTTIGFYLSSLMSVIGIYVFLYGQLYLVLSGLQKALVIEARVHNIESLETALASQSFIQLGLLTGLPMVMEIGLEKGFLTALKDFVLMQLQLAAVFFTFALGTKTHHYGRTIMHGGAKYRPTGRKVVVFHTSFTENYRLYSRSHFVKGFELLLLLIVYDLFRRSYESSMAYVLITYSVWFMSITWLFGPFLFNPSGFSWEKIVDDWKDWNKWIRQQGGIGIQQDKSWQSWWNDEQAHLRCSGLFSRLFEILLSLRFFLYQYGLVYHLDISQQSKNVLVYVLSWIVILAVFLLAKTVNIGRKKLSTNYQLRFRLFKAGLFITVLSTIIMLSKICQLSLRDLIVCCLAFLPTGWGLITIAQAVRPKIDDTGIWDFARVIAKAYDYGMGVVLFTPLAILAWLPIISAFQTRFLFNEAFNRHLHIQPILAGKRKKK

**>CsaCalS10-like_XP_030493451.1**

MAHNLDPDSDGRGVLQFKTGLMSVIKRKLAKRDGAPVDRNHDIEHLYKFYQRYKKRHRVDDIRREEQRMWESGTFGTDFAKMEMKYLEMKKIVATLRALVEVMEALSREADPRGAGGLIKEELRRIKSSEATLSREFAPYNIVPLEGPSRTNAIGIFPEVRGAISAIRYSEHFPRLPDDYVIIGDRDADMFDLLEVVFGFQKDNIRNQRENVVLSIANAQTRLEVPSEADPKIDEKAIDEVFLKLLDNYIKWCKYLRTRIAWFSLEAINRDRKLLLVSLYFLIWGEAANVRFLPECICYIFHNMAIELEAILDHGAAIPGAICVTASVSFLDKVICPIYKIMALEAKRNNNGKVAHSAWRNYDDFNEYFWSPACFELSWPMKRESPFFLEPKKKGNRIGKCSFVEHRTFLHLYRSFHRLWIFLALMFQTLAIIAFNDGRLDMDTFKSVLSIGPTFSIMNFIESFLDIILMFGSYTSARGMAISRQVIRFFWFGLCTTFVTYVYLKVLDERNSHNSNNSFYFRLYILVLGVYAGLHLVLGLLLNFPACHSLSAMSDQSFFQRLVLGLLLKFPVSHSLPAMPDQSFFKFFKRIYQECYYVGRDLYESLSDYCRYVLFWLVVFICKFCFAYFLQIQPLVSPTNTIVGLRNLDYSWHDLVPGANNNAMTIACLWGPVVAIYLIDIHIWYTLFSAVIGGVMGARARLGEIRSMEMVRKRFESFPEAFVKNLVYPQTKRLPFNRQLSQDSQDTDKTYAAMFSPFWNEIIKSLREEDYISNREMDLLSCPSNAGSLGLVQWPLFLLSSKISLAIDLAMDCKDTQGDLWNSICRDEYMAYAVRDCYYSIEKLLCSLIDGEGRLWVERIYREINNSILEGSLIITLSLKKLPVVLKKFTALTGLLLRNEDPDLSKGASKAVYELYEVVTHNLMSPDLREQLDTWNILARARNEGRLFSRIEWPKDTEAKELVKRLHLLLTVKDSAANIPKNLEARRRLEFFTNSLFMDMPSARPVSEIVPFCVFTPYYSETVLYSSSEFQKENKDGISTLFYLQKIFPDEWTNFLERIGWADSTGYAELQKSSSDALELRFWVSYRGQTLARTVRGMMYYRRALMLQSYLEKRSFRVDGYSQGSIPTSQGFELSRESRAQADIKFTYVVSCQIYGQQKQRKAPQAADISLLLQRNEALRVAFIQVEDGGADGKGPKEFYSKLVKADIHGKDQEIYSIKLPGDPKLGEGKPENQNHAIVFTRGEAVQTIDMNQDNYLEEAMKMRNLLEEFHRNHGLRPPTILGVREHVFTGSVSSLAWLMSNQETSFVTLGQRVLASPLKVRMHYGHPDVFDRVFHITRGGISKASRVINISEDIFAGFNSTLRQGNITHHEYIQVGKGRDVGLNQISLFEGKVAGGNGEQVLSRDVYRLGQLFDFFRMCSFFFTTVGFYVCSMMTVLTVYIFLYGRVYLAFSGLDEQIAKQAKRLGSTALDATLNAQFLVQIGIFTAVPMIMGFIIELGLLKAVFSFITMQLQLCSVFFTFSLGTRTHYFGRTILHGGAKYRATGRGFVVYHIKFAENYRLYSRSHFVKAFEVALLLIVYIAYGYTAGGAVSFELLTMSSWFLVFSWLSAPFIFNPFAFNWRRAVEDFDDWTSWLLCKGGVGVKGDDSWESWWEEELSHIQTLRGRVLETILSLRFFMFQYGIVYKLHLSAKERSLAIYLFSWVVLLVIVMVFKVFTYSPKKSAYFHLVLRFTQGVTSLGLIAAIALVVIFTDLSMRDLFASILAFIPTGWAIICLAVTWKRVFWSLGLWDSVREFAKMYDAAMGMLIFAPIALISWFPFRFSYRPLFNQAFTRGLEISTIIMDDARNKWVGRP

**>Lus10000266.g**

MRLIPPPENVDTLDHGVLRRFRRKLLKNYTGWCSYLNKKSNIWISDRSNPDLRRELLYVSLYLLIWGEAANLRFMPECICFIFHNMAMELNKVLEDYIDENTGQPVMPSFTGENAFLNSVVKPIYETVKAEVESSKNGTAPHTSWRNYDDLNEYFWSKRCFDKLKWPIDLGSNFFAISSKQQKKKHVGKTGFVEQRSFLNLFRSFDRLFVMLILFLHAAIVVAWEEKDYPWQALENKDVQVRALSIFFTWAGLRLLQSLLDIATQYRLVSRETMWLGVRMVMKSVVASGWILVFGVFYGRIWSHRNHDRRWSGEANVRVLTFLEVAGVFVLPELLALALFIIPWIRNFLENTNWRIFYFLTWWFQSRSFVGRGLREGLMDNIRYTLFWILVLATKFSFSYFLQIKPMIRPSRMMLDLKDVKYEWHEYFDNSNRLAVGLLWLPVVLIYLMDLQIWYSIYSSFVGAAVGLFAHLGEIRNIPQLKLRFQFFASAIQFNLMPVEQLLNDRGTLKSKFKDSIHRLKLRYGFGTPYKKLESNQVEAHKFALVWNEIIMIFREEDIISDQEIELLELPRNSWNVSVIRWPCFLLCNELLLALSQAKELVDAPDKWLWYKISKTEFRRCAVIEAYDSLKHLLLEIVKGNTEEHSIVTVLFQEIDHSLQIEKFTKTFSMTALPSFHTKLIKLLDLLKKPTNDSNQVVNIINTLQALYEISVRDFFREKRTIELLKEDGLVPRDPASISGHLFENAVELPDHTDENFYRNVRRLHTILTSRDSMHTVPKNLEARRRIAFFSNSLFMNMPHAPQVEKMMAFSVLTPYYSEEVVYGREQLRAENEDGVSTLYYLQTIYDDEWRNFLERMKREGMEKDNEIWTSKLRDLRLWASYRGQTLSRTVRGMMYYYRALKMLAFLDSASEIDIREGARELHPMGREAGQDGLSRSSSSMSLLFKGHEHGTAMMKFTYVVACQIYGTQKAKKDPNAEEILYLMKNNDALRVAYVDEVNAGRDMKDYYSVLVKYDAALDREVEIYRVKLPGPIKLGEGKPENQNHALIFTRGDAVQTIDMNQDNYFEEALKMRNLLQEYRQYYGIRKPTILGVREHIFTGSVSSLAWFMSAQETSFVTLGQRVLANPLKVRMHYGHPDVFDRFWFFTRGGLSKASRVINISEDIFAGFNCTLRGGNVTHHEYIQVGKGRDVGLNQISMFEAKVASGNGEQVLSRDVYRLGHRLDFFRMLSFFYTTVGFFFNTMVVILTVYAFLWGRLYLALSGVEASALANSSNNKALGAILNQQFIIQLGIFTALPMIVENSLEQGFLQAIWDFLTMQLQLSSVFYSFSMGTRSHFFGRTILHGGAKYRATGRGFVVQHKSFAENYRLYARSHFIKAIELGLILTVYASHSPVAKDTFVYIALTISSWFLVVSWIMAPFVFNPSGFDWLKTVYDFEDFMNWIWYKGGVFTKPEQSWEKWWYEEQDHLRTTGLLGKCMEIVLDLRFFFFQFGIVYQLGIAVKSTSITVYLISWVYIFVVVAIYVLVAYAHSKYAAKEHIYYRMVQFLIIILAILLMIALLEFTSFRIGDFFTSLMAFIPTGWGLLLIAQVFRPWIQSTVIWELVVSVSRLYDIMFGVIVMAPVAFLSWMPGFQAMQTRTLFNQAFSRGLRIMQIVTSKKSMI

**>Lus10001056.g**

MASTSGTKSDLPRSFSRASTMMVDLRDGDASVIDSEVVPSSLASIAPILRVANEIQDDNPRVAYLCRFHAFEKAHKMDPTSSGRGVRQFKPNKMAPQSVGRGFPQFKTYLLHRLEREESETEPRLARTDPREIQLYYQKFYEENIRDGQYTKRPEEMAKILQIASVLYDVLKTVVPASRVDEETHKYAKDVEKKREQYEHYNILPLYSVGLKPAIMELPEIKAAFNAVRNVDNLPMPRISPIYSAIHDVPTGKIKPVNDILEWLSSLFGFQKGNVANQREHLILLLANMDTRKRSLDDYTELNTSTIQQLMNKTFKNYLSWCHYLRVESNLRFPGGFDKPQLELIYIGLYLLIWGEASNVRFMPECICYIFHNRSTQRGSGRRKPKTNFVEVRTFWHLYRTFDRMWIFFILAFQAMFIIAWNAGSLAGIFDPDVFRSVLSIFITAAFLNLLRAILDIILNLYAWRSLKFTQILRYLLKFAVAAAWAVAMPIAYAKSVQNPTGLIKFFSSWASDWQNQSLYNYAVAIYLIPNILSILLFVLPPFQRTMEKSNWRIFTLLLWWAQASIFSTLNSRCLTVDVLAMHTCMIVHLSFPCPQILPLVEPTKLIMDMSIDNYQWHEFFPHAKHNLGAVIAIWAPIVLVYFMDTQIWYAIFSTIFGGIRGAFSHLGEIRTLGMLRSRFEDVPLAFSNCLVPSSKKRGKRKHLDESAERQSIANFSHVWNEIINSMRLEDLISNDERDLLLVPSSSNDVSVIQWPPFLLASKIPIALDMAKDFKGTNGADLFRRMDEYMRFAVIEFYETIRDIIYSLLQDDSDRIILERAHYATEDDESVKKEQRFGKINIDLIRNKTWREKVVRLHLLLTTKESAINVPSNLDARRRITFFANSLFMHMPSAPKVRDMLSFSVLTPYYKEDVLYSEEELNHENEDGITILFYLQTIYRDEWRNFEERISGSALKEKAELTRHWVSYRAQTLSRTVRGMMYYRRALELQYLLEFAGENAIINGLPSMELSNEDRSLSDRAQALADLKFTYVVSCQIYGAQKKSSDGRDRSCYNNILNLMLKYPSLRVAYIDEREEAVNGKQQKVYYSVLVKGGDKLDEEIYRIKLPGPPTEIGEGKPENQNHAIIFTRGEALQTIDMNQDNYFEEAFKMRNVLEEFLTPRHGPRKPTILGLREHIFTGSVSSLAWFMSNQETSFVTIGQRILANPLRVRFHYGHPDIFDRLFHITRGGISKASKIINLSEDIFSGFNSTLRGGYITHHEYIQVGKGRDVGMNQISLFEAKVANGNGEQTLSRDVYRLGRRFDFYRMLSFYFTTVGFYFSSMITVITVYIFLYGRMYMVMSGVEAEILTNPTIRQSNALEQALATQSIFQLGLLLVLPMVMEIGLEKGFRSALGDFIIMQLQLASVFFTFQLGTKAHYYGKTILHGGSKYRATGRGFVVFHAKFAENYRLYSRSHFVKGLELTMLLVIYEVYGESYRSSSLYFFITFSMWFLVGSWLFAPFVFNPSGFDWQKTVDDWTDWKRWMGNRGGIGIPPEKSWESWWDGEQEHLKHTNLRGRILDIILAFRFFVYQYGIVYHLDIAHRIKTLLAQSIPYVTLRAYIYVCVILLISGPQVYGLSWVVMITALLVFKMVSMGRRKFGTDFQLTFRILKALLFMGFLSVLTVLFVVCNLTITDLFASILAFLPTGWAILLIGQACRGLFKAIGLWDSIKELGRGYEYIMGLLIFSPTAILSWFPFVNEFQTRLLFNQAFSRGLQISMILQGRKDKDDAAAKKDKPPPPK

**>Lus10001424.g**

MASTSGTKSDLPRSFSRASTMMVDLRDGDASVIDSEVVPSSLASIAPILRVANEIQDDNPRVAYLCRFHAFEKAHKMDPTSSGRGVRQFKTYLLHRLEREESETEPRLARTDPREIQLYYQKFYEENIRDGQYTKRPEEMAKILQIASVLYDVLKTVVPASRVDEETHKYAKDVEKKREQYEHYNILPLYSVGLKPAIMELPEIKAAFNAVRNVDNLPMPRISSIYSAIHDVPTGKIKPVNDILEWLSSLFGFQKGNVANQREHLILLLANMDTRKRSLDDYTELNTSTIQQLVNKTFKNYLSWCHYLRVESNLRFPGGFDKPQLELIYIGLYLLIWGEASNVRFMPECICYIFHNMAYEVFGVLFRNLHPVSGETYESAAPDDEAFLRNVVTPIYQVVRKEARRNKSGKASHSRWRNYDDLNEYFWSNRCLRIKWPMDLKTDFFVHSDEVLPANERSTQRGSGRRKPKTNFVEVRTFWHLYRTFDRMWIFFILAFQAMFIIAWNAGSLAGIFYPDVFRSVLSIFITAAFLNLLRAVLDIILNLYAWRSLKFTQIVRYLLKFAVAAAWAVAMPIAYAKSVQNPTGLIKFFSSWASDWQNQSLYNYAVAIYLIPNILSTLLFVFPPFQRTMEKSNWRIFTLLLWWAQARIFSTLNSRYTLFWILLLISKLAFSYYIEILPLVEPTKLIMDMSIDNYQWHEFFPHAKHNLGAVIAIWAPIVLVYFMDTQIWYAIFSTIFGGIRGAFSHLGEIRTLGMLRSRFEDVPLAFSNCLVPSSKKRGKRKHLDESAERQSIANFSHVWNEIINSMRLEDLISNELRPFYRHFIYERDLLLVPSSSNDVSVIQWPPFLLASKIPIALDMAKDFKGTNGADLFRRMDEYMRFAVIEFYETIRDIIYSLLQDDSDRMIIRQICYEVDVSIQQQRFLQEFRTSGLPPLSDKLEKFLHTLVSNYDDPEMFKAQIINIIQDIIEIIVQDVMIRGREILERAHYATEDDESVKKEQRFGKINIDLIRNKTWREKVVRLHLLLTTKESAINVPSNLDARRRITFFANSLFMHMPSAPKVRDMLSFSVLTPYYKEDVLYSEEELNHENEDGITILFYLQTIYRDEWRNFEERISGSALKEKAELTRHWVSYRAQTLSRTVRGMMYYRRALELQYLLEFAGENAIINGLPSMELSNEDRSLSDRAQALADLKFTYVVSCQIYGAQKKSSDGRDRSCYNNILNLMLKYPSLRVAYIDEREETVNGKQQKVYYSVLVKGGDKLDEEIYRIKLPGPPTEIGEGKPENQNHAIIFTRGEALQTIDMNQDNYFEEAFKMRNVLEEFLTPRHGPRKPTILGLREHIFTGSVSSLAWFMSNQETSFVTIGQRILANPLRVRFHYGHPDIFDRLFHITRGGISKASKIINLSEDIFSGFNSTLRGGYITHHEYIQVGKGRDVGMNQISLFEAKVANGNGEQTLSRDVYRLGRRFDFYRMLSFYFTTVGFYFSSMITVITVYIFLYGRMYMVMSGVEAEILTNPTIRQSNALEQALATQSIFQLGLLLVLPMVMEIGLEKGFRSALGDFIIMQLQLASVFFTFQLGTKAHYYGKTILHGGSKYRATGRGFVVFHAKFAENYRLYSRSHFVKGLELTMLLVIYEVYGESYRSSSLYFFITFSMWFLVGSWLFAPFVFNPSGFDWQKTVDDWTDWKRWMGNRGGIGIPPEKSWESWWDGEQEHLKHTNLRGRILDIILAFRFFVYQYGIVYHLDIAHRIKTLLAQSIPYVTLRAYIYVCVILLISGPQVYGLSWVVMITALLVFKMVSMGRRKFGTDFQLTFRILKALLFMGFLSVLTVLFVVCNLTITDLFASILAFLPTGWAILLIGQACRGLFKAIGLWDSIKELGRGYEYIMGLLIFSPTAILSWFPFVNEFQTRLLFNQAFSRGLQISMILQGRKDKDDAAAKKDKPPPPK

**>Lus10003920.g**

MSSRRGFDQQQLPPRRILRTQTAGNLGETMLDSEVVPSSLVEIAPILRVANQVEISNPRVAYLCRFYAFEKAHGLDPTSSGRGVRQFKTALLQRLEKENETTLRGRTMSDAREMQKFYRDYYKKYIQALQNAADKADRAQLTKAYQTAAVLFEVLKAVNQTEAVPEEILEAHTKVEEKREIYVPYNVLPLDPDSENQAIMRYPEIQAAVSALRNVRGLPWSKNHKKKENEDILDWLQSMFGFQKDNVANQREHLILLLANVHMRRFPKPELQPKLDDHTLTEVMKKLFKNYKRWCKFLGRKSSLWLPTIQQEVQQRKLLYMGLYLLIWGEAANLRFLPECLCYIYHHMAFELYGILARSVSPLTGEHIQPAYPGKEEAFLWKVVKPIYDTIAKEARRSKGGRSKHSEWRNYDDLNEYFWSVDCFRLGWPMRADADFFRLPPEELHQNRDVEQKKSATGNRWIGKVNFVETRSFWHIFRSFDRLWSFFILCLQAMIIIAWNGSGNLSSVFGSDVLKKVLSIFITSAILNFLQAVLDVIFSFKARQIMPYYVKLRYILKVITAAAWVIILPVTYAYSWNDPTGFGRTIRGWFGNSPSTPSLFIMAVLIYLSPNMLSLLLFVVPFLRRVLERSNYRIVMLLMWWSQPRLYVGRGMHESSISLFKYTLFWVLLLVSKLAFSYYIEIKPLVGPTKAIMSVHIRRYQWHEFFPQAKNNIGVVIALWAPIVLVYFMDTQIWYAIYSTIFGGLYGAFRRLGEIRTLGMLRSRFQSLPGAFNACLVPVEKSETNKKKGLKATFSRRFPESPSNKEKQEARFAQMWNKIISSFREEDLINDREMNLMLVPYWADRELELIQWPPFLLASKIPIAVDMAKDSNGKDRELKKRLVSDNYMLCAVRECYASIKSIANYLILGDREILVINEIFTKVDEYIDNETIIKELNMSALPILNEQFVKLVEYLLENKREDKDQVVILLLDMLEVVTRDILEDEVPSLLESSHGGFSGKQDGMISLDQHQKHQIFGELRFPVPQSDAWNEKIRRLHLLLTVKESAMDVPSNLEARRRISFFSNSLFMDMPDAPKVRNMLSFCVLTPYYTEDVLYSLNILEKPNEDGVSVLFYLQKIFPDEWTNFLQRVGCSSEEELRSTEELEEELRLWASYRGQTLTKTVRGMMYYRKALELQAFLDLATDEELMKGYKAAEANSEQHSKRQRSLWAQCQAITDMKFTYVVSCQNYGIHKRSGDARANDILRLMTTHPSLRVAYIDEVEETSKDRLKRSVEKVYYSALVKAAPPTTPIDSSERVQNLDQVIYRIKLPGPALLGEGKPENQNHAIIFTRGEGLQTIDMNQDNYMEEAFKMRNLLEEFLVKHGGVRCPTILGLREHIFTGSVSSLAWFMSNQENSFVTIGQRLLAHPLKVRFHYGHPDVFDRLFHLTRGGISKASRGINLSEDIFAGFNSTLREGNVTHHEYIQVGKGRDVGLNQISMFEAKIANGNGEQTLSRDIYRLGHRFDFFRMLSCYFTTIGFYFSTLITVLTVYVFLYGRLYLALSGLEEGLINQRAIRDNKPLQVALASQSVVQIGILMALPMMMEIGLERGFRNALSDFVLMQLQLAPLFFTFSLGTKTHYYGRTLLHGGAEYRGTGRGFVVFHAKFAENYRLYSRSHFVKGIELMILLLVYHIFGHSYRGVLAGVLITISIWFMVVTWLFAPFLFNPSGFEWQKILDDWTDWHKWINSRGGIGVPPEKSWESWWESEHAHLRHSGIRGIIAEILLALRFFIFQYGLVYHLSIINKAKTFLVYGISWLVIALILSIVKAISVGRRRLSANFQLVFRLIKGLIFLTFLATFATLIAVLHMTLQDVVVCILAFMPTGWGLLLIAQACKPVIQRAGFWGSVRVLARGYEIVMGLLLFIPVAFLAWFPFVSEFQTRMLFNQAFSRGLQISRILGGPRKDRSTRSSKE

**>Lus10007327.g**

MASTSGTKSDHHLPRSLSRASTIMVDLRDGDAPVIDSEVVPSSLASIAPILRVANEIQDDNPRVAYLCRFHAFEKAHKMDPTSSGRGVRQFKTYLLHRLEREESETEPRLARTDPREIQLYYQKFYEENIRDGQYTKKPYGLLLAPYSFLFTIILEEMGKILQIASVLYDVLRTVVPESRVEEEVSFLNVLTPYYKEDVLYSEEELNHENEDGITILFYLQTIYRDEWRNFEERIAGSPLKDKAELTRHWVSYRAQTLARTVRGMMYYRRAVELQYLLEFAGENAIINGLPSTELSSEDRALSDRAQAVADLKFTYVVSCQIYGAQKKSSDARDRSCYNNILNLMLKYPSLRVAYIDEREETVNGKQQKVYYSVLVKGGDKLDEEIYRIKLPGPPTEIGEGKPENQNHAIIFTRGEALQSIDMNQDNYFEEAFKMRNVLDEFVTPRRGSRKPTILGLREHIFTGSVSSLAWFMSNQETSFVTIGQRILANPLRVRFHYGHPDIFDRLFHITRGGISKASKIINLSEDVFAGFNSTSRGGYITHHEYIQVGKGRDVGMNQISLFEAKVANGNGEQTLSRDIYRLGRRFDFYRMLSFYFTTI

GFYFSSMITVLTAYVFLYGRLYMVLSGVEAAIMTNPIVRQTKALEQALATQSVFQLGLLLVMPMVMEIGLEKGFRSALGDFIIMQLQLASVFFTFQLGTKSHYYGKTILHGGSKYRATGRGFVVFHAKFAENYRLHSRSHFVKGLELMMLLVIYGVYGESYRSSNLFFFISFSMWFLVCSWLFAPFVFNPSGFDWQKTVDDWTDWKHWMGNRGGIGIPPEKSWESWWDGEQEHLRYTNLRGRIMEIFLASRFFLYQYGIVYHLDIAHPLKSLLVYGLSWVVMITALLVFKMVSMGRRKFGTDFQLMFRILKALLFLGFLSVLTVLFVVCNLTISDLFVSTLAFIPTGWALLLIGQAWRGMFKGIGLWDSIKELGRAYEYIMGILIFTPIAVLSWFPFVNEFQTRLLFNQAFSRGLQISMILQGRKDKDNADAAAAAKKDKPLPPQAANK

**>Lus10013744.g**

MASSSRMGSDQATPPQPQQRRIMRTQTAGNLGESIFDSEVVPSSLVEIAPILRVANEVESSNPRVAYLCRFYAFEKAHRLDPTSSGRGVRQFKTALLQRLERENDPTLKGRVKKSDAREMQSFYQHYYKKYIQALQNAADKADRAQLTKAYQTANVLFEVLKAVNMTQSIEVDREILEAQDKVAEKTQLYVAYNILPLDPDSANQAIMRYPEIQAAVVALRNTRGLPWPKDHKKKDEDILDWLQAMFGFQKGNVANQREHLILLLANVHIRQFPKMDQQPKLDDRALTDVMKKLFKNYKKWCKYLDRKSSLWMPTIQQEVQQRKLLYMGLYLLIWGEAANLRFMPECLCYIYHHMAFELYGMLAGNVSPMTGENVKPAYGGEEEAFLTKVVTPIYNVIAKEAERSRKGKSKHSQWRNYDDLNEYFWSVDCFRLGWPMRADADFFSLPAEQHHFEKDGDNNKPAYKDRWVGKVNFVEIRTFWHIFRSFDRMWSFFILCLQAMIIIAWNDSAGRPIFTGDVVKKVLSVFITAAILKLGQAVLFLFPFVRRFLEQSHYRIVMLMMWWSQPPLYVARGMHESTLALFNYTVFWVLLIITKLAFSYFIEIRPLVGPTKAIMSVHISTFQWHEFFPQARNNIGVVIALWAPIILVYFMDAQIWYAIFSTIFGGIYGAFRRLGEIRTLGMLRSRFQSLPGAFNARLMPEERSEPKKKGLRATLSRNFATIPSNKEKEAARFAQLWNKIITSFREEDLISNREMDLLLVPYWADRDLDLIQWPPFLLASMIPIALDMAKDSNGKDKELKKRIEAESYMSCAVRECYASFRNIIKFLVQGDREKEVIEYIFEEVDKHIEAGDLISEYKMSALPNLYEHFVHISSLVDSIHGGPGHEGMVPLEQQYQLFASSGAIKFPIQPVTEAWKEKIKRLDLLLTTKESAMDVPSNLEARRRISFFSNSLFMDMPTAPKVRNMLSFSVLTPYYTEEVLFSLRELEVPNEDGVSILFYLQKIYPDEWNNFLERVNCTGEEELKGIDDLEEELRLWASYRGQTLTRTVRGMMYYRKALELQAFLDMARDEDLMEGYKAIELNTELHLRGERSLLAQCQAIADMKFTYVVSCQQYGIHKRSADPRAQDTLRLMTAYPSLRVAYIDEVEEPNKDKSKKVNQKVYYSVLVKAASPKAIDSSEPVQHLDEVIYRIKLPGPAILGEGKPENQNHAIIFTRGEGLQTIDMNQDNYMEEALKMRNLLQEFLKKHDGVRYPTILGLREHIFTGSVSSLAWFMSNQETSFVTIGQRLLANPLKVRFHYGHPDVFDRLFHLTRGGVSKASKVINLSEDIFAGFNSTLREGNVTHHEYIQVGKGRDVGLNQISMFEAKIANGNGEQTLSRDIYRLGHRFDFFRMLSCYFTTVGFYFSTLITVLIVYVFLYGRLYLVLSGLEEGLKNQRAIRDNKPLQVALASQSFVQIGFLMALPMLMEIGLERGFRTALSEFVLMQLQLAPVFFTFSLGTKTHYYGRTLLHGGAKYRPTGRGFVVFHAKFADNYRLYSRSHFVKGIEMIILLVVYEIFGQTYRSAVAYVLITISMWFMVGTWLFAPFLFNPSGFEWQKIVDDWTDWNKWISNRGGIGVPPEKSWESWWEEEQEHLHHSGKRGIVAEILLSLRFFIYQYGLVYHLSITKKAKTKSFLVYGISWLVIFLVLFVMKTVSVGRRKFSANFQLVFRLIKGLIFITFISILVVLIALPHMTIQDIIVCILAFMPTGWGMLLIAQACKPVVHRAGFWGSVRTLARGYEILMGLLLFTPVAFLAWFPFVSEFQTRMLFNQAFSRGLQISRILGGQRKDRSSRNKE

**>Lus10014796.g**

MPRVYDNWDRLVRATLKQEQLRSAGQGPERTGSGIAGAVPPSLVHSTNIDAILQAADEIQADDPNVARILCEQAYTMAQNLDPNSGGRGVLQFKTGLMSVIKQKLAKRDGTPIDRNRDIEHLWEFYQRYKKRHRVDDIQREEQKWGESGTFTTANLGDLELRSLDMKKVFSTLRALIEVMESLSKDADPHGVGKLIMEELRRIKKAGELTPYNIVPLDAPSLANAIGVFPEVRGAISAIRYTEQFPRLPPDLEISGEYADMFDLLEYVFGFQNGNVRNQRENVVLMIANAQSRLGIPTQADPKIDENAINEVFLKVLDNYIKWCKYLRIRLAWNSNQAVNKERKLFLVSLYFLIWGEAANVRFLPECICYIFHHMAKELDAVLDHGEADRATSCKDESGSVSFLDKVILPIYETLAAEVERNNNGKAAHSAWRNYDDFNEYFWSPACFELKWPMNKDSSFLLKPKKWKRTGKSTFVEHRTFLHLYRSFHRLWIFLALMFQALTIIAFHHGKINLGTFKVILSVGPSFAIMNFIKSCLDVLLMFGAYTTARAMAISRLVIRFFWGGLTSVAITSALGLWTATEISNLSYSERYYVGRGLYERLSDYCSKLDLIQIRPLVSPTNIIRNFPSSRLQYSWHDLVSKSNNNALTIVSLWAPVVAIYLMDIHIWYTLLSAVIGGIMGARDRLGEIRTLDMVHKRFESFPAAFVKNLVSPQATRLPFNAQAPQESQDSNKAYAALFSPFWNEIIKCLREEDFISNREMDLLSVPSNKGSLRLVQWPLFLLSSKILLAVDLALDCKDTQRDLWNRVCKDEYMAYAVQECYYSVEKILHSLVDGISSLSSIDICFSYGRVETIFREINNSILEESLVITVSLKKLPLVLSRFTALTGLLIRNETPDLAKGAANAVYQLYEVVTHDLLSSDLRERFDTWNILARARNEGRLFSRIEWPRDPEIKEQVKRLHLLLTVKDSAANIPKNLEARRRLEFFSNSLFMDMPAPKPVSEMMPFCVFTPYYSETVLYSLSELRVENEDGISTLFYLQKIFPDEWENFLERIGRGESTGDADLQENSSDSLELRFWASYRGQTLARTVRGMMYYRRALMLQSYLESRSLGVDDYSQTGATTEGFGLSRESRAQADLKFTYVVSCQIYGQQKQRKAPEATDIALLLQRNEALRVAFIHVDETGSTDGKVTKVYHSKLVKADAHGKDQEVYSIKLPGDPKLGEGKPENQNHAIVFTRGEAIQAIDMNQDNYFEEALKMRNLLEEFRANHGIRHPTILGVREHVFTGSVSSLAWFMSNQETSFVTLGQRVLAKPLKVRMHYGHPDVFDRIFHITRGGVSKASRVINISEDIYAGFNSTLRQGNVTHHEYIQVGKGRDVGLNQIALFEGKVAGGNGEQVLSRDVYRLGQLFDFFRMLSFYISSVGFYVCTMMTVLTIYIFLYGRTYLAFSGVDRAVAREARILGNTALDAVLNTQFLVQIGVFTAVPMIMGFILELGLLQAVFSFITMQLQLCSVFFTFSLGTRTHYFGRTILHGGAKYRATGRGFVVRHIKFAENYRLYSRSHFVKALEVALLLIVYIAYGFTRGGAVSFILLTLSSWFLVISWLFAPYIFNPSGFEWQKTVEDFDNWTTWLMYKGGIGVKGDQSWESWWDEEQTHIQTLRGRILETILTLRFFLFQYGIVYKLQLTGKDTSLAIYGFSWAVLVGIVLVFKVFTYSPKRSINFQLLMRFTQGVTSMGLIAALCLVVAFTNLSITDLFASILAFIPTGWAILCLAITWKRIVRSLGLWESVREFARMYDAGMGMVIFAPIAFLSWFPFVSTFQSRLLFNQAFSRGLEISLILAGNKANVDM

**>Lus10020750.g**

MQPKLYVGRGMHEDLFTLLKYTLFWILLLISKLAFSYYVEILPLVEPTKLIMDMTIDNYQWHEFFPHAKHNIGAVVAIWAPIVLVYFMDAQIWYAIFSTIFGGISGAFSHLGEIRTLGMLRSRFEDVPSAFSDCLVPSSVTRGNRDHLDESAESQSIANFSHVWNEFINSMRVEDLISNDERDLLLVPSSSNDVSVIQWPPFLLASKIPIALDMAKDFKGTNGGDLFRRMDDYMRFAVVEFYETIRDIIYSLLQDDSDRMAIRQICYEVDLSIQQQKFLQEFRTSGLPMLSEKLEKFLHTLLSNYEDPDMFRAQIINILQDIIEIIVQDVMIRGREILERADDETVKKEQRFGKINIDLIRNKTWREKVVRLHLLLTTKESAINVPSNLDARRRITFFANSLFMNMPKAPKVRDMLSFSVLTPYYKEDVLYSEEELNHENEDGITILFYLQTIYRDEWRNFEERIACSPLKDKPELTRHWVSYRAQTLARTVRGMMYYRRAVELQYLLEFSGENAIINGLPSTELSSEDRALSDRAQALADLKFTYVVSCQIYGAQKKSSDARDRSCYNNILNLMLKYPSLRVAYIDEREETVNGKQQKVYYSVLVKGGDKLDEEIYRIKLPGPPTEIGEGKPENQNHAIIFTRGEALQSIDMNQDNYFEEAFKMRNVLDEFVTPRRGSRKPTILGLREHIFTGSVSSLAWFMSNQETSFVTIGQRILANPLRVRFHYGHPDIFDRLFHITRGGISKASKIINLSEDVFAGFNSTSRGGYITHHEYIQVGKGRDVGMNQISLFEAKVANGNGEQTLSRDIYRLGRRFDFYRMLSFYFTTVGFYFSSMITVLTAYVFLYGRLYMVMSGVEAAIMTNPIVRQSKALEQALATQSVFQLGLLLVMPMVMEIGLEKGFRSALGDFIIMQLQLASVFFTFQLGTKSHYYGKTILHGGSKYRATGRGFVVFHAKFAENYRLHSRSHFVKGLELMMLLVIYDLYGDLCNPSGFEWQKTVDDWTDWKRWMGNRGGIGIPPEKSWESWWDGEQEHLRYTNLRGRIMEIFLASRFFLYQYGIVYHLDIAHRIKSLLVYGLSWVVMITVLLVFKMVSMGRRKFGTDFQLMFRILKALLFLGFLSVLTVLFVVCNLTISDLFVSTLAFIPTGWALLLIGQAWRGMFKGIGLWDSIKELGRAYEYIMGLLIFTPIAVLSWFPFVNEFQTRLLFNQAFSRGLQISMILQGRKDKDNADAAAAAKKDKPLPPQAAAK

**>Lus10020893.g**

MPNQEASPHLVRRSSRSAATTTFSTIEVFDNSVVPSSLESIKPILRVANEIQNERPRVAYLCRFYAFEKAHRLDPNSSGRGVRQFKTALLQRVERAYEPAGVLLEVLYAVNKTEKVEEVAPEIIAAARDIQEKKEIYAPYNILPLDSAGASQSIMQLEEVKAAVAALWNTRGLSWPSSFEQHRQKAGDLDLLDWLRAMFGFQRDSVRNQREHLILLLANNHIRLNPKPEPLNKARALDERAVDAVMHKIFKNYKNWCKFLGRKHSLRLPQSQPEIQQRKILYMGLYLLIWGEAANVRFIPECLCYIFHNMAYELHGLLAGNVSIVTGENIKPSYGGDDEAFLRKVITPIYRVIAKEASKSQNGTASSTEWCNYDDLNEYFWSSDCFSLGWPMRDDGAFFISTRHAAKGKDTPQANSGSTGKSYFVETRTFWHIFRSFDRLWTFYVLAFQLMIIYAWSGVAIQNILRRDVLYYLSSIFITAAFLRLLQSILDVVLNFPGYHRWKFTDVLRNILKIIVSFAWAVILPLCYTGSFKQVIGFANGMTFLRTVKSIPPIYLLAVVIYMTPNILAAALFIFPMLRRWIENSDWLIIRLLLWWSQPRIYVGRGMHESQFSLIKYTIFWVLLLCSKFTFSFFIQIKPLVKPTKDIMNIRHVEYTWHEIFPYAKHNYGAVLSLWAPTILVYFMDTQIWYSIFSTLYGGFEGAVDRLGEIRTLGMLRSRFQSLPGAFNAFLVPSDKKRRKGFSLSKRFAEVSANRRSEAAKFAQLWNEVITSFREEDLISDRKGYTTEIDNMDLLVVPYTSDPSIKLIQWPPFLLASKIPIALNMAAQFRSKDSDLWKRICADEYMKCAIIECYESFKQVLNILVIGENEKRIIGIIIKEIESHVSKGTLLANFRMASLPALCEKVVVLVGILKDGDPSKRDNVVLLLQDMLELVTRDLMVNENRELVDVGHSGKDSGRQLFAGTDPRPAIVFPPPVTAQWDEQIRRLHLLLTVKESAMDVPANLEARRRISFFTNSLFMDMPRAPRVRKMLSFSVMTPYYSEETVYSKSDLEMENEDGVSIIYYLQKIYPVRGMMYYRRALKLQAFLDMANESEILAGYKAITNPTEEDKKSQRSLSAQLEAVADMKFTYVATCQIYGNQKRKGDRHATNILNLMVNNPSLRVAYIDEIEERDGAKTQKVCYSVLVKAVDNLDQEIYRIKLPGPAKLGEGKPENQNHAIVFTRGEAMQAIDMNQDNYLEEAFKMRNLLEEFHEDHGVRPPTILGVREHIFTGGVSSLAWFMSNQETSFVTLGQRVLARPLKIRFHYGHPDVFDRIFHITRGGVSKASRGINLSEDIFAGFNSTLRRGNVTHHEYIQVGKGRDVGLNQISLFEAKVACGNGEQTLSRDIYRLGHRFDFFRMLSFYYTTIGFYISSMVVVFTVYAFLYGRLYLALSGLEGSIIKFAKHRGDTALRAAMASQSVVQLGLLTALPMVMEMGLERGFRTALGEIIIMQLQLAAVFFTFSLGTRVHYFGRTVLHGGAKYRATGRGFVVRHEKFAENYRMYSRSHFVKALELLLLLICYQIYGKAVSGIAYMLVTSSMWFLVCSWLFAPFLFNPSGFEWQKVVDDWDDWTKWINSRGGIGVPANKSWESWWEEEQEHLQHTGISGRICEVVLALRFFIYQYGIVYQLKVTKATSAGREHSISVYGLSWLVIVAMMLILKTVSTGRKKFSADFQLMFRLLKFILFIGFVVTLIILFTTLHLTVGDVFQSLLAFLPTGWAILQISQACKPIVKGLKMWGSVKALGRGYEYMMGVLLFLPIAVLAWFPFVSEFQNRLLFNQAFSRGLQIQRILSGGKKHKWGLLLIAQAVRPKIE

**>Lus10030030.g**

MSDTVRRRATPSSDPGAQGSGTRHDETFNMIPIHNLLATDDPPLRYPEVRPAIKALLDLGDLRRPPNVQWQHSMDLLDWLALFFGFQRDNVKNQREHLVLHLANSQMRLIATRPEENADALNHEVLRLFRRKLLKNYTGWCSYLNVKSNVRNLNRPDVRLELLYVSLYLLIWGEAANLRFMPECICFIFHNMATELNRIVDDVGTGRPLMPSYTGENAFLSSVVKPIYEVVKAEAEKSKNGTAPHMSCRNYDDINEYFWSRSCLVKLNWPIDLKSDFFEEISGHGKRGFVEQRSFLNLFRSFDRLFVMLILFLQAAVIVAGQHKEYPWQALGYRDVQVEVLTVFLTWAGLRFLQSMLDIGMQYRLVSRDTIWVGVRMVLKSGVAVGWIIVFGVLYVRIWSQKNQDGRWSAAANARVVNFIHSVLVFVSPELLTIVLCVVPWLRNCLEDTDWRIFHLLSWWFQSRSYVGRRLSEGIADSIKYSLFWIVVLATKFAFSYFLQIKPMFRPSEVLLGLDDVEYEWQALYNCHNALAVGLLWLPVVLIYLMDLQIWYSIYSSLVGAAVGLFAHLGEIRNIRQLRLRFQFFAGAFQFNLMSEEHQRQYGSFFQRKKSIIKDSMHRSKLLHGFGQPYKKLESNQIEANKFSLLWNEIIMTFREEDIISDREVELLELPHNSWNVRVIRWPCFLICNELSLAFKQATQLPEDYNDKWLWYKISKNEYRRCAVIEAYDSVKYLLLTIVKANTEEHSIVIFWFQEIDDLLQNGKFTQTFTRDDLFRLEAGLTRLVRLLKIDTKVLNLQVVKILQSLYEIVGGRLKKHRKDPGGHLLFENAVQLPRRLADQAFYMNVRRLRTILCTVKDSMENVPRNPEARRRIAFFCNSVFMQMPRAPRVEKMMAFSVLTPYYNEEVVYSQELLVAQSDDGNSTLRHLQTVYLDEWSNFVERMLRQGLARDDELWKTKFTDIQLWASCRGQTLSRTVRGMMYYFRALKMLAFLDSVSEIGVTEEGAREQDRFSRTATMKFTYVVACQTYGTQKANNDPNAEEILHLLKNNDALRVAYVDEVKVDRLDHNVTDYYSVLVKYDAELNEEVEIYRVKLPGPIKIGEGKPENQNHALIFTRGDALQTIDMNQDNYFEEAIKMRNLLQEYGRFHGIRKPTILGVREHIFTGSISSLAWFMSAQEMSFVTLGQRVLANPLKVRMHYGHPDVFDRFWFLTRGGLSKASKVINISEDIFAGFNSTLRGGNVTHHEYIQVGKGRDVGLNQISMFEAKVASGNGEQVLSRDAYRLGHRLDFFRMLSLFYTTVGFYFNTMVAILTVYAFLWGRLYLALSGFEGSALANRRTSNIKSLGALLNQQFIIQLGIFTALPTIVENSIEQGFLKSIWDSLIMQLQLSSVFYSFSMGTRSHFFGSTILHGGAKYRATGRGFVLTHTRFSENYRLYAQSHFVKAIELGLILIVYASCSPVANDTLVYTLLTVSSWFLVISWMIAPFVFNPSGFDWLKTASDFEDFVNWFRYDKYMLAPAEQSWEKWWEEEQDYLRTTGLMGKCAEIALNLRFFLFQFGIVYQLGIGSKSTTLAVYFISWVFNIAVFAAYLLLAYTHRKYAVRKQIYYRLVQFIVTVVAVTIVVLLLVFTSFRFGDLFAISLGFISTGWGVVLIARVFRPWLQKKKKWGLVVSVARLYDMMFGMTVMMPVAVLSWIPGVRTMQTRILYTRAFSKGLHIWDMVARNRSSSR

**>Lus10031648.g**

MSAIRYRAPPPGRPRPNHQSPGEDEEPFNIIPVHNLLADHPSLRYPEVRAAAAALRAVGNLRRPPYVQWHPSMDLLDWLALFFGFQRDSVRNQREHLVLHLANAQMRLTPPPDNIDTLDHGVLRRFRRKLLKNYTSWCSYLNKKSNIWISDRSNPDVRRELLYVSLYLLIWGESANLRFMPECICFIFHNMAMELNKILEDYMDENTGQPIMPSFSGENAFLNSVVKPIYDTVKAEVENSKNGTAPHTSWRNYDDINEYFWSRRCFEKLKWPIDLGSNFFVIDSRQKHVGKTGFVEQRSFLNLFRSFDRLFIMLILFLQAAIIVAWAEKGYPWQALESREVQVRVLSVFFTWAGLRFIQSLLDIIMQRRLVSRETMWLGVRMVAKAVVATGWILVFSVFYARIWSQRDRDRGWSGAANRRVVTFLEVAGVFVLPEILATVLFIIPWVRNFLENTNWRIFYLLSWWFQSRSFVGRGLREGLVDNIKYSLFWILVLATKFSFSYFLQIKPMVRPSKLMLDLKDVTYEWHEFFDNSNRFAVGLLWLPVVLIYLMDLQIWYSIYSSFVGAAVGLFDHLGEIRNLPQLRLRFQFFASALQFNLMPEEQLLNARGTLRSKFKDSIHRLKLRYGLGRPYKKLESNQVEANKFAIVWNEIIMIFREEDIISDREVELLELPQNSWNVQVIRWPCFLLCNELLLALSQAKELVDAPDKWLWYKICKNEYRRCAVIEAYDSIKHLLLEIIKVNTEEHSIVTILFQEIDHSLQIEKFTKTFKMTALPNFHVKLIKLLDLLMKPTKDVNQVVNTLQALYEIAIRDFFREQRTVEQLKEDGLVPHDPAAMAGHLFENAVELPDSAQETLYRNVRRLHTILTSRDSMHTVPKNLEARRRIAFFSNSLFMNMPHAPQVEKMMAFSVLTPYYSEEVIYSREQLRTENEDGIATLYYLQTIYDDEWKNFIERMRREGMLNTDEIWTTKLRDLRLWASYRGQTLSRTVRGMMYYYRALKMLAFLDSASELDIREGARELGSMRRDSGLNGQGSERFSSSRRLSRNSSSVSLLFKGHEYGTAIMKFTYVVACQIYGTQKAKKDPHAEEILYLMKNNDALRVAYVDEKTTGRDVTEYFSVLVKYDAELEREVEIYRVKLPGPLKLGEGKPENQSHALIFTRGDALQTIDMNQDNYFEEALKMRNLLEEYKQYYGIRKPTILGVREHIFTGSVSSLAWFMSAQETSFVTLGQRVLANPLKVRMHYGHPDVFDRFWFMTRGGLSKASRVINISEDIFAGFNCTLRGGNITHHEYIQVGKGRDVGLNQISMFEAKVASGNGEQILSRDVYRLGHRLDFFRMLSFFYTTVGFFFNTMMVILTVYAFLWGRLYLALSGVEGSALADASNNRALGAILNQQFIIQLGLFTALPMIVENSLEQGFLQAIWDFLTMQLQLSSVFYTFSMGTRSHYFGRTILHGGAKYRATGRGFVVQHKSFAENYRLYARSHFVKAIELGLILIVYATHSPVAKATFVYIALTISSWFLVMSWIMAPFVFNPSGFDWLKTVYDFDDFMNWIWYRGSVFAKAEQSWEKWWEEEQDHLRTTRLLGKCVEIVLDLRFFFFQFGIVYQMGIAAKSTSIFVYLLSWIYIFVVVAIFVLIVYAREKYAAKEHIYYRLVQFLVIVLAILVMIALLEFTSLSFADIFTSMLAFIPTGWGMLLIAQVFRPWLQSTILWELVVSVARLYDIMFGVIVMTPVAFLSWMPGFQAMQTRILFNEAFSRGLRIMQIVTGKKSML

**>Lus10032463.g**

MSGAEELWERLVRAALRSERTRAAAFAGAVTGIAGNVPSSLENNRDIDGILRAADEIEDEDPNISRILCEHGYSLAQDLDPNSEGRGVLQFKTGLMSVIKQKLTKREGGTIDRGQDIARLQEFYKLYRERNRVDTLKEEEMKLRESGAFSGNLGELERRTIRRKRVFATLKVLGSVLQQLNKDMPDELSRMIKSDASMTEDLIAYNIIPLDLQTVTNAVVAFPEVRAAVSSLRYFRGLPILPEDFPIPSTRASDMFDFLHYVFGFQKDNVSNQRENVVHLLANEQSRLGIPDPTEPKLDEAAVQNVFMKALGNYINWCSYLCIQPVWSNSEALSTEKKLLYVSLYFLIWGEAANIRFLPECLCYIFHHMAREMDEILRQQNAQPANSCISEDDARARVSFLDQVILPLYGVIAAEASNNDNGRAAHSAWRNYDDFNEYFWSLHCFDLSWPWRLSSTFFQKPKPRTKVLLKTAGSQRRGKTSFVEHRTFLHLYHSFHRLWIFLVMMFQGLAIIAFNDGRFNTRTLREILSLGPTFVVMKFSESVLDVLMMYGAYSTTRHVAVSRILLRFVWFACASVFICFLYVKALQEPDTSSVLFKLYV

IVIGIYAGVQFFLGFLTRIPACHLMTNQCDQWSIVRFVKWMRQERYYVGRGMYERTSDFIKYMVFWLVILSAKFSFAYFLQIKPLVEPTKIIVKMTDNIQYSWHDLVSKNNHNALTIVSLWAPVVAIYLLDIHVFYTLTSAVWGFLLGARDRLGEIRSLESVHKLFEEFPAAFMRTLHSDRTVGNALEPVEKKKIDAAQFSPFWNEIIKNLREEDYIANFELELLQMPRNSGNLPLVQWPLFLLANKIFLARDIAAESRDSQLELWERISRDEYMKYAVEECYHALRYILTEIFEGEGRMWVERVYEDIQASIQNKSIHVDFQLTKLALVIQRVTALLGVLKEAETSDMEKGAIKAVQDLYDVIQHDVLSIDKREHYDTWNLLSKARTEGRLFTNLKWPRDPELRTQIKRLHSLLTIKDSAANVPNNIEARRRLEFFTNSLFMDMPLAKPVREMLSFSVFTPYYSEIVLYSMAELLKKNEDGISILFYLQKIYPDEWKNFLARIGRDENSVDTELFDSPTDILELRFWASYRGQTLARTVRGMMYYRKAIMLQSYLERGTAQDVESAIGSKDATDTQGFELSPEARAQADIKFTYVVTCQIYGKQKEEQKPEAADIALLMQRNEALRVAFIDEVETLKEGHVQREFFSKLVKADINGKDKEIYSIKLPGNPKLGEGKPENQNHAIVFTRGNAVQTIDMNQDNYFEEALKMRNLLEEFHRDHGIRPATILGVREHVFTGSVSSLASFMSNQETSFVTLGQRVLSNPLKVRMHYGHPDVFDRVFHITRGGISKASRVINISEDIFAGFNSTLRQGNITHHEYIQVGKGRDVGLNQIAVFEGKVAGGNGEQVLSRDVFRLGQLFDFFRMMSFYFTTVGYYFCTMLTVLTVYMFLYGKAYLALSGVGETIQERAQILQNTALSAALNTQFLFQIGIFTAVPMVLGFILEQGFLRAVVSFITMQLQLCSVFFTFSLGTRTHYFGRTILHGGARYQATGRGFVVRHIKFSENYRLYSRSHFVKGLEVAVLLIVYLAYGYNEGGALSYILLTVSSWFMALSWLYAPYLFNPSGFEWQKTVEDFRDWTNWLLYRGGIGVKGEESWEAWWEEELAHIRTFSGRIIETILSLRFFIFQYGIIYKLDVQRNDTSLTVYGISWAVLAVLIVLFKVFTFSQKISVNFQLLLRFIQGVAFLLALAGLAVAVVFTNLSVPDIFACILAFIPTGWGILSIAAAWKPLMKKVGLWKSIRSIARLYDAGMGMIIFIPIAFFSWFPFMSTFQTRLMFNQAFSRGLEISLILAGNNPNTRL

**>Lus10033689.g**

MSAIRYRATPPGRPRPNHQSPEEDEEPFNIIPVHNLLADHPSLRYPEVRAAAAALRAVGNLRRPPYVQWHPSMDLLDWLALFFGFQRDSVRNQREHLVLHLANAQMRLTPPPDNIDSLDPGVLRRFRRKLLKNYTSWCSYLNKKSNIWISDRSNPDVRRELLYVSLYLLIWGESANLRFMPECICFIFHNMAMELNKILEDYMDENTGQPIMPSFSGENAFLNSVVKPIYETVKAEVENSKNGTAPHTSWRNYDDINEYFWSRRCFEKLKWPIDLGSNFFVIDSRQKHVGKTGFVEQRSFLNLFRSFDRLFVMLILFLQAAIIVAWAEKGYPWQALESREVQVRVLTVFFTWAGLRFIQSLLDIIMQRRLVSRETMWLGVRMVAKAVVAAGWILVFSVFYARIWSQQDRDRGWSGAANRRVVTFLEVAGVFVLPEILATVLFIIPWVRNFLENTNWRIFYLLSWWFQSRSFVGRGLREGLVDNIKYSLFWILVLATKFTFSYFLQIKPMVRPSKLMLDLKDVTYEWHEFFNNSNRFAVGLLWLPVVLIYLMDLQIWYSIYSSFVGAAVGLFAHLGEIRNLPQLRLRFQFFASAIQFNLMPEEQLLNARGTLKSKFKDSIHRLKLRYGLGRPYKKLESNQVEANKFALVWNEIIMIFREEDIISDREVELLELPQNSWNVQVIRWPCFLLCNELLLALSQAKELVDAPDKWLWYKICKNEYRRCAVIEAYDSIKHLLLEIIKVNTEEHSIVTILFQEIDHSLQIEKFTKTFKMTALPNFHVKLIKLLDLLMKPTKDVNQVVNTLQALYEIAVRDFFREKRTVEQLKEDGLVPHDPAAMAGHLFENAVELPGPAQETFYRNVRRLHTILTSRDSMHTVPKNLEARRRIAFFSNSLFMNMPHAPQVEKMMAFSVLTPYYSEDVIYSREQLRTENEDGIATLYYLQTIYDDEWKNFIERMRREGMLKTDEIWTTKLRDLRLWASYRGQTLSRTVRGMMYYYRALKMLAFLDSASELDIREGARELGSMRRDSGLNGQGSERFSSSRRLSRNSSSVSLLFKGHEYGTAMMKFTYVVACQIYGTQKAKKDPHAEEILYLMKNNDALRVAYVDEKTTGRDVTEYFSVLVKYDAELEREVEIYRVKLPGALKLGEGKPENQNHALIFTRGDALQTIDMNQDNYFEEALKMRNLLEEYKQYYGIRKPTILGVREHIFTGSVSSLAWFMSAQETSFVTLGQRVLANPLKVRMHYGHPDVFDRFWFMTRGGLSKASRVINISEDIFAGFNCTLRGGNITHHEYIQVGKGRDVGLNQISMFEAKVASGNGEQILSRDVYRLGHRLDFFRMLSFFYTTVGFFFNTMMVILTVYAFLWGRLYLALSGVEGSALADASNNRALGAILNQQFIIQLGLFTALPMIVENSLEHGFLQAIWDFLTMQLQLSSVFYTFSMGTRSHYFGRTILHGGAKYRATGRGFVVQHKSFAENYRLYARSHFVKAIELGLILIVYATHSPVAKATFVYIALTISSWFLVMSWIMAPFVFNPSGFDWLKTVYDFDDFMNWIWYRGSVFAKAEQSWEKWWEEEQDHLRTTGLLGKLVEMVLDLRFFFFQFGIVYQMGIAAKSTSIFVYLLSWIYIFVVVAIFVLIVYAREKYAAKEHIYYRLVQFLVIVLAILVMIALLQFTSLSFSDIFTSMLAFIPTGWGMLLIAQVFRPWLHSTILWELVVSVARLYDIMFGVIVMTPVAFLSWMPGFQAMQTRILFNEAFSRGLRIMQIVTGKKSML

**>Lus10037469.g**

MSSRGGFDQQQLPPRRILRTQTAGNLGETMLDSEVVPSSLVEIAPILRVANQVELSNPRVAYLCRFYAFEKAHGLDPTSSGRGVRQFKTALLQRLEKENETTLRGRTMSDAREMQKFYRDYYKKYIQALQNAADKADRAQLTKAYQTAAVLFEVLKAVNQTEAVPEEILEAHTKVEEKREIYVPYNVLPLDPDSKNQAIMRYPEIQAAVSALRNIRGLPWSKNHKKKENEDILDWLQSMFGFQKDNVANQREHLILLLANVHMRRFPKPELQPKLDDHTLTEVMKKLFKNYKRWCKFLGRKSSLWLPTIQQEVQQRKLLYMGLYLLIWGEAANLRFLPECLCYIYHHMAFELYGLLARSVSPLTGEHIQPAYRGEEEAFLWKVVKPIYDTIAKEAGRSKGGRSKHSEWRNYDDLNEYFWSVDCFRLGWPMRADADFFRLPPEELHQNRDVEKKSATVNRWIGKVNFVETRSFWHIFRSFDRLWSFFILCLQAMIIIAWNGSGNLSSVFGSDVLKKVLSIFITSAILNFLQAVLDVIFSFKARQIMPYYVKLRYILKVITAAAWVIILPVTYAYSWNDPTGFGRTIRGWFGNSPSTPSLFIMAPRLYVGRGMHESSISLFKYTLFWVLLLVSKLAFSYYIEIKPLVGPTKAIMSVHIRTYQWHEFFPQAKNNIGVVIALWAPIVLVYFMDTQIWYAIYSTIFGGLYGAFRRLGEIRTLGMLRSRFQSLPGAFNACLVPVEKSESNKKKGLKATFSRRFQESPSSKEKQEARFAQMWNKIISSFREEDLINDREMNLMLVPYWADRELELIQWPPFLLASKIPIAVDMAKDSNGKDRELKKRLVSDNYMLCAVRECYASIKSIANYLILGEREILVINEIFSKVDEYIDNETIIKELNMSALPILNEQFVKLVEYLLENKREDKDQVVILLLDMLEVVTRDILEDEVPSLLESSHGGFYGKQDGMISLDQHQKHQIFGELRFPVPQSDAWNEKIRRLHLLLTVKESAMDVPSNLEARRRISFFSNSLFMDMPDAPKVRSMLSFCVLTPYYTEDVLYSLNTLEKPNEDGVSVLFYLQKIFPDEWTNFLQRVGCSSEEELRSTEELEEELRLWASYRGQTLTKTVRGMMYYRKALELQAFLDLATDEELMKGYKAAEANSEQHSKRQRSLWAQCQAITDMKFTYVVSCQNYGIHKRSGDTRANDILRLMTTHPSLRVAYIDEVEETSKDRAKRSVEKVYYSALVKAAPPTTPIDSSERVQNLDQVIYRIKLPGPALLGEGKPENQNHAIIFTRGEGLQTIDMNQDNYMEEAFKMRNLLEEFLVKHGGVRCPTILGLREHIFTGSVSSLAWFMSNQENSFVTIGQRLLAHPLKVRFHYGHPDVFDRLFHLTRGGISKASRGINLSEDIFAGFNSTLREGNVTHHEYIQVGKGRDVGLNQISMFEAKIANGNGEQTLSRDIYRLGHRFDFFRMLSCYFTTIGFYFSTLITVLTVYVFLYGRLYLALSGLEEGLINQRAIRDNKPLQVALASQSVVQIGILMALPMMMEIGLERGFRNALSDFVLMQLQLAPLFFTFSLGTKTHYYGRTLLHGGAEYRGTGRGFVVFHAKFAENYRLYSPIHFLTGIELMILLLVYHIFGHSYRGVLAGVLITISIWFMVVTWLFAPFLFNPSGFEWQKILDDWTDWHKWINSRGGIGVPPEKSWESWWESEHAHLRHSGIRGIIAEILLALRFFIFQYGLVYHLSIINKAKTFLVYGVSWLVIALILSIVKAISVGRRRLSANFQLVFRLIKGLIFLTFLATFATLIAVLHMTLQDVVVCILAFMPTGWGLLLIAQACKPVIQRAGFWGSVRVLARGYEIVMGLLLFIPVAFLAWFPFVSEFQTRMLFNQAFSRGLQISRILGGPRKDRSTRSNKE

**>Lus10039199.g**

MASSSRMGSDQATPPQPQQRRIMRTQTAGNLGESIFDSEVVPSSLVEIAPILRVANEVESSNPRVAYLCRFYAFEKAHRLDPTSSGRGVRQFKTALLQRLERENDPTLKGRVKKSDAREMQSFYQHYYKKYIQALQNAADKADRAQLTKAYQTANVLFEVLKAVNMTQSIEVDREILEAQDKVAEKTQLYVAYNILPLDPDSANQAIMRYPEIQAAVVALRNTRGLPWPKDHKKKDEDILDWLQAMFGFQKGNVANQREHLILLLANVHIRQFPKMDQQPKLDDRALTDVMKKLFKNYKKWCKYLDRKSSLWMPTIQQEVQQRKLLYMGLYLLIWGEAANLRFMPECLCYIYHHMAFELYGMLAGNVSPMTGENVKPAYGGEEEAFLTKVVTPIYNVIAKEAERSRKGKSKHSQWRNYDDLNEYFWSVDCFRLGWPMRADADFFSLPAEQRHFEKDGDNNKPAYKDRWVGKVNFVEIRTFWHIFRSFDRMWSFFILCLQAMIIIAWNDSAGRPIFTGDVVKKVLSVFITAAILKLGQVEMYSEGHVEAVTNAALLIDSAVLDVILSWKARQIMSFHVKLRYILKVVSAAAWVVVLPVTYAYTWENPPGFARTIKSWFGNSNSSPTLFILAVVIYLSPNMLSAVLFLFPFVRRFLEQSHYRIVMLMMWWSQPPLYVARGMHESTLALFNYTVFWVLLIITKLAFSYFIEIRPLVGPTKAIMSVHISTFQWHEFFPQARNNIGVVIALWAPIILVYFMDAQIWYAIFSTIFGGIYGAFRRLGEIRTLGMLRSRFQSLPGAFNARLMPEERSEPKKKGLRATLSRNFATIPSNKEKEAARFAQLWNKIITSFREEDLISNREMDLLLVPYWADRDLDLIQWPPFLLASMIPIALDMAKDSNGKDKELKKRIEAESYMSCAVRECYASFRNIIKFLVQGDREKEVIEYIFEEVDKHIEAGDLISEYKMSALPNLYEHFVKLIKYLLVNKPEDRDQVVILFQDMLEVVTRDIMMEDHISSLVDSIHGGPGHEGMVPLEQQYQLFASSGAIKFPIQPVTEAWKEKIKRLDLLLTTKESAMDVPSNLEARRRISFFSNSLFMDMPTAPKVRNMLSFSVLTPYYTEEVLFSLRELEVPNEDGVSILFYLQKIYPDEWNNFLERVNCTGEEELKGIDDLEEELRLWASYRGQTLTRTVRGMMYYRKALELQAFLDMARDEDLMEGYKAIELNTELHLRGERSLLAQCQAIADMKFTYVVSCQQYGIHKRSADPRAQDTLRLMTAYPSLRVAYIDEVEEPNKDKSKKVNQKVYYSVLVKAASPKAIDSSEPVQHLDEVIYRIKLPGPAILGEGKPENQNHAIIFTRGEGLQTIDMNQDNYMEEALKMRNLLQEFLKKHDGVRYPTILGLREHIFTGSVSSLAWFMSNQETSFVTIGQRLLANPLKVRFHYGHPDVFDRLFHLTRGGVSKASKVINLSEDIFAGFNSTLREGNVTHHEYIQVGKGRDVGLNQISMFEAKIANGNGEQTLSRDIYRLGHRFDFFRMLSCYFTTVGFYFSTLITVLIVYVFLYGRLYLVLSGLEEGLKNQRAIRDNKPLQVALASQSFVQIGFLMALPMLMEIGLERGFRTALSEFVLMQLQLAPVFFTFSLGTKTHYYGRTLLHGGAKYRPTGRGFVVFHAKFADNYRLYSRSHFVKGIEMIILLVVYEIFGQTYRSAVAYVLITISMWFMVGTWLFAPFLFNPSGFEWQKIVDDWTDWNKWISNRGGIGVPPEKSWESWWEEEQEHLHHSGKRGIVAEILLSLRFFIYQYGLVYHLSITKKAKTKSFLVYGISWLVIFLVLFVMKTVSVGRRKFSANFQLVFRLIKGLIFITFISILVVLIALPHMTIQDIIVCILAFMPTGWGMLLIAQACKPVVHRAGFWGSVRTLARGYEILMGLLLFTPVAFLAWFPFVSEFQTRMLFNQAFSRGLQISRILGGQRKDRSSRNKE

**>Lus10039828.g**

MSATRRRAPPPNQSPREEDEELFNIIPVHNLLADHPSLRNPEVRAAAAALRAVGNLRRPPYVQWNPSMDLLDWLALFFGFQRDNVRNQREHLVLHLANAQMRLIPPPENVDTLDHGVLRRFRRKLLKNYTGWCSYLNKKSNIWISDRSNPDLRRELLYVSLYLLIWGEAANLRFMPECICFIFHNMAMELNKVLEDYIDENTGQPVMPSFTGENAFLNSVVKPIYETVKAEVESSKNGTAPHTSWRNYDDLNEYFWSKRCFDKLKWPIDLGSNFFAISSKQQKKKHVGKTGFVEQRSFLNLFRSFDRLFVMLILFLHAAIVVAWEEKDYPWQALENKDVQVRALSIFFTWAGLRLLQSLLDIATQYRLVSRETMWLGVRMVMKSVVASGWILVFGVFYGRIWSHRNHDRRWSGEANVRVLTFLEVAGVFVLPELLALALFIIPWIRNFLENTNWRIFYFLTWWFQSRSFVGRGLREGLMDNIRYTLFWILVLATKFSFSYFLQIKPMIRPSRMMLDLKDVKYEWHEYFDNSNRLAVGLLWLPVVLIYLMDLQIWYSIYSSFVGAAVGLFAHLGEIRNIPQLKLRFQFFASAIQFNLMPVE

QLLNDRGTLKSKFKDSIHRLKLRYGFGTPYKKLESNQVEAHKFALVWNEIIMIFREEDIISDQEIELLELPRNSWNVSVIRWPCFLLCNELLLALSQAKELVDAPDKWLWYKISKTEFRRCAVIEAYDSLKHLLLEIVKGNTEEHSIVTVLFQEIDHSLQIEKFTKTFSMTALPSFHTKLIKLLDLLKKPTNDSNQVVNIINTLQALYEISVRDFFREKRTIELLKEDGLVPRDPASISGHLFENAVELPDHTDENFYRNVRRLHTILTSRDSMHTVPKNLEARRRIAFFSNSLFMNMPHAPQVEKMMAFSVLTPYYSEEVVYGREQLRAENEDGVSTLYYLQTIYDDEWRNFLERMKREGMEKDNEIWTSKLRDLRLWASYRGQTLSRTVRGMMYYYRALKMLAFLDSASEIDIREGARELHPMGREAGQDGLSRSSSSMSLLFKGHEHGTAMMKFTYVVACQIYGTQKAKKDPNAEEILYLMKNNDALRVAYVDEVNAGRDMKDYYSVLVKYDAALDREVEIYRVKLPGPIKLGEGKPENQNHALIFTRGDAVQTIDMNQDNYFEEALKMRNLLQEYRQYYGIRKPTILGVREHIFTGSVSSLAWFMSAQETSFVTLGQRVLANPLKVRMHYGHPDVFDRFWFFTRGGLSKASRVINISEDIFAGFNCTLRGGNVTHHEYIQVGKGRDVGLNQISMFEAKVASGNGEQVLSRDVYRLGHRLDFFRMLSFFYTTVGFFFNTMVVILTVYAFLWGRLYLALSGVEASALANSSNNKALGAILNQQFIIQLGIFTALPMIVENSLEQGFLQAIWDFLTMQLQLSSVFYSFSMGTRSHFFGRTILHGGAKYRATGRGFVVQHKSFAENYRLYARSHFIKAIELGLILTVYASHSPVAKDTFVYIALTISSWFLVVSWIMAPFVFNPSGFDWLKTVYDFEDFMNWIWYKGGVFTKPEQSWEKWWYEEQDHLRTTGLLGKCMEIVLDLRFFFFQFGIVYQLGIAVKSTSITVYLISWVYIFVVVAIYVLVAYAHSKYAAKEHIYYRMVQFLIIILAILLMIALLEFTSFRIGDFFTSLMAFIPTGWGLLLIAQVFRPWIQSTVIWELVVSVSRLYDIMFGVIVMAPVAFLSWMPGFQAMQTRTLFNQAFSRGLRIMQIVTSKKSMI

**>Lus10040891.g**

MDIHIWYTLLSAVIGGIMGARDRLGEIRTLDMVHKRFESFPAAFVKNLVSPHVTRLPFNAQASQESQDSNKAYAALFSPFWNEIIKCLREEDFISNREMDLLSVPSNKGSLRLVQWPLFLLSSKILLAVDLALDCKDTQRDLWNRVCKDEYMAYAVQECYYSVEKILHSLVDGEGRLWVETIFREINNSILEESLVITVSLKKLPLVLSRFTALTGLLIRNETPGLAKGAANAVYQLYEVVTHDLLSSDLRERFDTWNILARARNEGRLFSRIEWPRDPEIKEQVKRLHLLLTVKDSAANIPKNLEARRRLEFFSNSLFMDMPAPKPVSEMMPFCVFTPYYSETVLYSLSELRVENEDGISTLFYLQKIFPDEWENFLERIGRGESTGDADLQENSSDSLELRFWASYRGQTLARTVRGMMYYRRALMLQSYLESRSLGVDDYSQTGATTEGFGLSRESRAQADLKFTYVVSCQIYGQQKQRKAPEATDIALLLQRNEALRVAFIHVDETGSTDGKVTKVYHSKLVKADAHGKDQEVYSIKLPGDPKLGEGKPENQNHAIVFTRGEAIQAIDMNQDNYFEEALKMRNLLEEFRANHGIRHPSILGVREHVFTGSVSSLAWFMSNQETSFVTLGQRVLAKPLKVRMHYGHPDVFDRIFHITRGGVSKASRDNYFEEALKMRNLLEEFRANHGIRHPTILGVREHVFTGSVSSLAWFMSNQETSFVTLGQRVLAKPLKVRMHYGHPDVFDRIFHITRGGVSKASRVINISEDIYAGFNSTLRQGNVTHHEYIQVGKGRDVGLNQIALFEGKVAGGNGEQVLSRDVYRLGQLFDFFRMLSFYISSVGFYVCTMMTVLTIYIFLYGRTYLAFSGVDRAVAREARILGNTALDAVLNTQFLVQIGVFTAVPMIMGFILELGLLQAVFSFITMQLQLCSVFFTFSLGTRTHYFGRTILHGGAKYRATGRGFVVRHIKFAENYRLYSRSHFVKALEVALLLIVYIAYGFTRGGAVSFILLTLSSWFLVISWLFAPYIFNPSGFEWQKTVEDFDDWTTWLMYKGGIGVKGDQSWESWWDEEQAHIQTLRGRILETILSLRFFLFQYGIVYKLQLTGKDTSLAIYGFSWAVLVGIVLVFKVFTYSPKRSINFQLLMRFTQGVTSMGLIAALCLVVAFTNLSITDLFASILAFIPTGWAILCLAVTWKRIVRSLGLWESVREFARMYDAGMGMVIFAPIAFLSWFPFVSTFQSRLLFNQAFSRGLEISLILAGNKANVDM

**>Lus10042478.g**

MSEIVVADPIIDPGASSSSYVRRKGGETAASSYEVTRSLTYGDAGASTSGQGSNGPEPFDSERLPPTLTREIQRFLRVANLIETEEPRIAYLCRFHAFEIAHNMDHNSTGRGVRQFKTSLLQRLEQDEETTLRRRKEKSDIRELRRVYHAYKDYILKSGAGFDLDDRHREKLINASRIASVLFEVLKTVTNAAGTKALAEREGISSKSEFYVPYNILPLDHGGIQQAIMQFPEIKAAVMAIRNTRGLPPPEDFHRRGAFLDLFEFLQCCFGFQEGNVANQREHLILLLANTYIRLCYKQASNSKLVDGAVDELMKKFFKNYANWCKFLGRKNNIRLPYVKQEAQQYKILYIGLYLLIWGEAANLRLMPECLCYIFHQMANELHDILTSAVSLETGEKMKPAYGGDFESFLNHVVTPIYMVISKEAEQGRGTADHSTWRNYDDLNEFFWSPDCFQLGWPMRTDHDFFFSPSAKKKEVKGAAKKKKKKREVEVDEELRGQEENEAGATTEKQEPKWLGKTNFVEVRSFWQIFRSFDRMWTFFILSLQAMIVMACHDLENPLEILDAEVFEDILSIFITSAILRFAQAILEIAFTWKARHTMDILEKRKQALKLAFGVIWTIVLPVLYAKSRSNYTCYSKRHGSWSGQFCISPYMVAVAIYMTTNAVEMVLFFVPAVRKFIEISNCQIFRTFSWWTQPRLYVGRGMQETQVKPLIEPTRLILKIGVQKYDWHELFPKVKSNAGAIVAIWAPIIVVYFMDTQIWYSVFCAIFGGVYGILHHLGEIRTLGMLRSRFHTLPYAFYVSLIPPSAKSDQKARGFFYKRSHNGFESGTSGVAKFALVWNQIINAFRQEDLISNRELDLMSIPMSSELLSGMIRWPIFLLTNKLSTALSIAQEFQGKDESLFRKIKKDKYMYCAVKECYESLKYVLEILIVGEQEKRVVLSILNEVEDSIARSSFLKDFKISELPALRAKCVDLLELLVEGDEINHANVVQVLQDMFELVTNDMMTTSSRIWELLSYSPKLEEDTPYFSVKIEPQLFDSAAGKDAIHFPFPDSGPLKEQIKRFLHLLTVGDKAMDIPANLEAKRRISFFATSLFTDMPVAPKVRNMLSFSVLTPHFMEDVTYSRKDLRSSKKEEVSILFYMQKIYPDEWENFLERMGWENSDASNEENINELRNWASFRGQTLSRTVRGMMYYREALRVQAFLEMAEDEDILEGYDAAERNNRTLSAQLDALADMKFSYVLSFQKFGSLKSIGDPRALDVLDLMTRYPSFRVAYVEEKEEIVSGKHPKVYSSVLVKAVNGLDQEIYRIKLPGEPNIGEGKPENQNHAIIFTRGEALQTIDMNQDNYFEEAFKMRNLLQEFLQRQGRRPPTILGLREHIFTGSVSSLAWFMSYQETSFVTIGQRLLSNPLRVRFHYGHPDVFDRVFHITRGGISKASKTINLSEDVFAGFNSTLRRGCITYNEYLQVGKGRDVGLNQISKFEAKVANGNSEQTICRDIYRLARGFDFFRMLSCYFTTIGFLFSNMISVIGVYVFLYVQLYLVLSGLQKSLLFEARMHDIQSLETALASQSFIQLGLLTGLPMVMEIGLEKGFLTALKDFVLMQLQLAAVFFTFSLGTKIHYYGRTILYGGAMYRPTGRKVVVFHASFTENFRLYARSHFVKGFELVILLIVYDLFRRSYQSSMAYVMITYSIWFMSITWLFAPFLFNPSGFTWEKIVDDWKDWNKWIRQQGGIGIQQDKSWQSWWNDEQTHLRQAGVGARLGEIILSIRFFMYQYGLVYHLDISQHSRNFLVYVLSWVVIVAIFLLVKAVDMGRQLFSAKYHLGFRLFKAFLFIAVVTIIVTLSLACQLSLRDLIVCCLAFLPTGWGLLLIAQAVRPKIEETVIWDFTEVLGKAYDYGMGVILLAPIAVLAWLPIISAFQTRFLFNEAFNRHLQIQPILAGKKKQR

**>Lus10042959.g**

MSGAEELWERLVRAALRSERTRSAAFAGAVTGIAGNVPSSLENNRDIDDILRAADEIEDEDPNISRILCEHGYSLAQNLDPDSERRGVLQFKTGLMSVIKQKLTKREGGTIDRGQDIARLQEFYKLYRERNRVDTLKEEEMKLRESSAFSGNLGELERRTIRRKRVFATLKVLGSVLQKLNKDMPDELSRMIESDASMSEDLIAYNIIPLDLQTVTNAVVAFPEVRAAVSSLKYFRGLPILPESFPIPSTRASDMFDFLHYVFGFQKDNVSNQRENVVHLLANEQSRLGIPDATEPKLDEAAVQNVFMKALENYINWCSYLCIQPVSSNLEALSTEKKLLYVSLYFLIWGEAANIRFLPECLCYIFHHMAREMDEMLRQQNAEFANSCCSEGGACVSFLDQVILPLYGVISAEASNNDNGRAAHSAWRNYDDFNEYFWSLHCFDLSWPWRLSSTFFKKPNPRTKGLAIIAFNDGRFNTRTLREILSLGPTFVVMKFSESVLDVLMMYGAYSTTRHVAVSRILLRFVWFACASVFICFLYVKALQEPDTSSVLFKLYVIVIGIYAGVQFFLGFLTRIPACHLMTNQCDQWSLVRFVKWMRQERYYVGRGMYERTSDFIKYMVFWLVILSAKFSFAYFLQIKPLVEPTKIIVKMTDNIQYSWHDLVSKNNHNALTIVSLWAPVVAIRSLESVHKLFEEFPIAFMRTLHSNRTVGNAPQPVEERKINAAQFSPFWNEIIKNLREEDYIANFELELLQMPRNSGNLPLVQWPLFLLANKIFLARDIAAESRDSQLELWERISRDEYMKYAVEECYHALRYILTEIFEGEGRMWVERVYEDIQASIQNRSIHVDFQLTKLALVIQRVTALMGVLKEAETSDMEKGAIKAVQDLYYFFQHDGLSIDKREHYDTWNLLSKARTEGRLFTNLKWPRDPELRTQIKRLHSLLTIKDSAANVPNNIEARRRLEFFTNSLFMDMPLAKPVREMLSFSVFTPYYSEIVLYSMAELLKKNEDGISILFYLQKIYPDEWKNFLARIGRDENSVDTELFDSPTDILELRFWASYRGQTLARTVRGMMYYRKAIMLQSYLERGTAQDVEAAIGSKDATDTQGFELSPEARAQADIKFTYVVTCQIYGKQKEEQKPEAADIALLMQRNEALRVAFIDEVETLKEGRHVQREFFSKLVKADINGKDKDNYFEEALKMRNLLEEFHRDHGIRSATILGVREHVFTGSVSSLASFMSNQETSFVTLGQRVLSNPLKVRMHYGHPDVFDRVFHITRGGISKASRVINISEDIFAGFNSTLRQGNITHHEYIQVGKGRDVGLNQIAVFEGKVAGGNGEQVLSRDVFRLGQLFDFFRMMSFYFTTVGYYFCTMLTVLTVYMFLYGKAYLALSGVGETIQERAQILQNTALSAALNTQFLFQIGIFTAVPMVLGFILEQGFLRAVVSFITMQLQLCSVFFTFSLGTKTHYFGRTILHGGARYQATGRGFVVRHIKFSENYRLYSRSHFVKGYPSSSVSTCFSARNRFLMELGITRPIIFVRRLEVAVLLIVYLAYGYNEGGALSYILLTVSSWFMALSWLYAPYLFNPSGFEWQKTVEDFRDWTNWLLYRGGIGVKGEESWEAWWEEELAHIRTFSGRIMETILSLRFFIFQYGIIYKLDVQRNDTSLTVYGISWAVLAVLIILFKVFTFSQKISVNFQLLLRFIQGVAFLLALAGLAVAVVFTNLSVPDIFACILAFIPTGWGILSIAAAWKPLMKKIGLWKSIRSIARLYDAGMGMIIFIPIAFFSWFPFMSTFQTRLMFNQAFSRGLEISLILAGNNPNTSCAVAGLVSLSPFSARLVLDFDRCRLALILRASLVVITPPSISEVVVLDDPDLIHVNFFWILVVANKLRHRLLLDERRQSSPMRGRQSSPMRGRQSSP
