# Supplementary material for: Identification of Callose Synthases in Stinging Nettle and Analysis of Their Expression in Different Tissues
Source: Int J Mol Sci. 2020 May 28;21(11):3853. doi: 10.3390/ijms21113853 (PMC7313033; doi:10.3390/ijms21113853)
Supplement: Supplementary file 1 [file ijms-21-03853-s001.zip › Supplementary File 2.docx]

**Promoter sequences used to identify conserved motifs with MEME**

**CLADE 1**

>Lus10040891.g

CTTTTGATCCTTCTCTGTTGCTTCTGCTCTACTGTAGCAACAATATATATAGTATTATCATTTAATATGATAATATCTGATTCCTTTTGCTTCCAAGCTTCCCAGCTTACCAACATGATAGACATTGTGACAAGCTGTGTGACTTGTGTGCTAGTTATTTGTATCCTTATTTGTCTCATCTCTCTCTGCAGATCTATCTC

>Lus10014796.g

TTGTTTGGTTTGAAACTGGCGCTTTCCAATATTCTACTGTGTTGATTTGATTTTCCCCTTCGACCTTGGGGGTATCGTGGATCGAATTACCTCGGGCTATCATTTGGTGGGAGCCTGAAAGCGTTCTCGACTCGGCAGTGGAGGGAGTGACGAATCTAGGGTTTTGGGGTTTTGCTTTTGTTTGATACGTCGAATCAGAG

>UdCalS10

ATTTTTTCCCGCGATCTCCGCGTTTTCATCGACTGCATTTTCGTTTCCCATTTCTCTGTCTGTTGTTTCCGTGTCCGAACTCGGAATATTCGCCGATTCGACTCGCTTCCAAACCTTCCGAGTGGATTCATCACCGCTCTCGAAGCTCGTCATTTGCAGAAATTTGGAAAATATTGTCGTTGTTCGCATTTTCGTTGCGCAATCCGAACCATTTGCCGATCCGATTCAGCCGAAATCTCCGAGTCCGCTGTTTTTTTTTTTTTTTTTTTTTTTTTTTNAGCTTATTTTCTGAATATCTGACGCTGAGAAGAAGGAAAGAAACTACCTCTAGGGCTCGAAGTTGTTGCCGATTGGGCTTCCTAGCC

>CsaCalS10

TTTAATCATCCTTTACCTTTCTAACCAGGGACACCTTCTTTTCCCGCTATCTCCGCGTTTCGTCTACTCGTGTATCCTAAAATTAATTTCTTCTAACGTAAAAAAACCTTTCATTTCGAAAACAACCTTCAGAATCGTACACGATTCAACTCGGCCACGACCGACTCGGTTTGGTTTATTCTTCATCATCTGATTTTGAAATCTCAGGGTTAAATCTAGGGCTTGGGGGAAGATTTTGATCTGTCTTTCCTAGCAGCC

>CsaCalS9

TTTATTTATTAAATTAAATGAAAAAGCTTTAAAACAAAAAAGAACAGAGCAAAAAAAGAAGCTGAGTTTTGAAAGAGAGATTCTTCTTCTTCTTCTGAGCTCAAAGACCGATTCGACTCGGTCTCTAGGGTTTTCTTCGCTTTTCTGGTTTTTACTTACAGAAA

>CsaCalS10-LIKE

TTAAGTAAAGATAGACAGTCCTGCAGACATGCGTGTATTTTTAGTTTGTTCCCTTATTTACAAAAGTATGTTTAAATTTTAGTGTGTGAGCAGGCACACAGT

>Lus10042959.g

GGCGCAGTTTTTTTGCTGGGATTCAATGCAAGGATATGTTTAGGAATTCGATCCAGTAGTAGAACATGAAACGAATAATTTTCGTGGACGAAATTTGAATTTTTGCCCAACAAACATCTGCTTGTTTGTTCATTTTGATTCTCATTCTTACTATTATTGTCTGCATCTTACCTCGGAAACTTGATGTTATGCTGCAGAGA

>Lus10032463.g

GTCGCAGCTTTTTTGCTGGTAGTCAATGCAAGGATCTGTTTAGGAATTCGATCCAGTAGTAGAACATGAAACGAATAATTTTCGTGGACGAAATTTGAACTTTTGCCTAACAAACATCTTTTTGTTTGTTCAGTTTGATTCTCATTCTTACTATTATTGTGTGCATCTTACCTCGGAATCTTGATGTTATGCTGCAGAGA

>AtCalS9

TTCGTTGATTACAACTAGTCCTAGGTTAAAGAACAACGTTTACAACTTATTTTGCTTTTTTCAGTTGTGCCTAGGTCACTTGTCCGAATCTGTATAATTCACGTGTATCTTCCTGGATTTGATTACATTTCATTTACTTAGTGTTGGGTTTTACGTTAGTTTAAGCTGCTGAGTACTTTGATGTTTTTTGTTTGTAGAAA

>AtCalS10

TGAGAAAACCCACTTTACGAAGGAATCAAATCGACTCGTTTTCTTAAATCCGCCATAGATCCCATCCGACACACCACCGTCACATCCGGGTTCGAATCCTCCAGCTCGAGCTATTCGCGTTTCTTCATCTTCTCTCTTTGTGCCATGGTGTTTCACCCTCTTTAGGGACATCACAAGCTTTGGTTTGAGAGAATTTGAAATCCTCCTCTAGGATTTGAAAATT

**CLADE 2**

>Lus10031648.g

ATTTGTTTCTTCCAGTTCAAAATAAAATCATTATTTTATTATTATTACTTACTTTAATCCTTCAGCTGTAAAGAATCCTTCACGTCGGTCTTCACCCACAACTTTAAAACTCAAACGCTTTTCAATCAAATCTATCTCTCTCAAACGGCCGGAAAAGAAAGAAATAAAACTGTCTCCGGCGACCGTCGCCGTCAGATGCT

>Lus10033689.g

ATCATTATTTTATTATTATTATTATTATTATTATTATTATTTATTACTTACTTTGATCTTTCAGCTGTAAAGAATCCTTCACGTCTTCGGTCTTCACCCACAACTTAAAACTCAAAAGCTTTTCACTCAAATCTATCTCTCCGTCTCTCTCTCAAACTGCCGGAAAAAACTGTCTCCGGCCACCGTCGCCGTCAAATGCT

>Lus10030030.g

TTAAGATTGCGTCATCGTTACAGACAAGCACACTGACGCTGAGCTCACCTCACTGCAAGTCTGCAAGACTGCAACACACCTGAACAAGAAAGTTCTTCCAAACTTCCCTCCATTTTTTCATCATCATTTCCCTGCTTCACTATTTCATTTCAAGTCCACCGGATTCTTCCCACGCCAAAAACCCGCCGCCTCGGAATCCA

>Lus10039828.g

AATATACACGTCATATTTATCCTAAGAAGAAAATTTCCAAGTCAGTAATGACGTGTAATTATAGCAATAAATACCGAAAAATCCGTTAAAATTCGGATTCCCTCCTTTCTCGGATTACCATTTTTAACCCTGCTCTTTCAAACTTTCTCCAATCGCTGCCGTCAGCTAAGCTAAACTGCCAATCGCTGCCGTCAGATGCA

>UdCalS11

AACCCCCTCTCTCTCTCTCTCTCTCTCTCTCAAAAAATGAAACCAGAAAGACAAAAAGAAAATTAAAAAAGAAAAATAAAGAG

>UdCalS12

TACATTACGGAAACTCTCCCAATTGAGCAAAGAAAAATATTGGTATATTGACATCAAATCTATTTAAAAACTTGTTCATAAATGTACGCGCAGATCTGAAGAAAGAGTCCGAAACGCGCCTCTTTTATCATTTCTCTCTCTCTCAAAACTCTCCACACCCAAACAAACACTCTCATTCTCTCTCTCCCNACAAACACTCTCATTCTCTCTCTCTCCCCCATTTCCACCGATCTATTGAAATCTCGCATCGG

>CsaCalS12-LIKE

GTTTGTAAGAAACGCGCCTTGTTTCGTTTCATTCTTCTTCTCCTTCCCTCGTCATCTTCCCCTATTTCTGCATCTGAGCACAATCTCAGTCTCTCTCTCTCTCTTCTCTCTCCCTCTGAAACTGATTGGATTGTTTGGGGTTTCAAATCCCTAATCTTGTTTTTCTTCAACAAAAAAAGATAATATACCTTCGAGTATTCGGAGGTTTTGAGCTGCAACTTTTTC

>CsaCalS11-LIKE

TCATATAAATACACTTCTTCACTCTATTCAATGGTAACTGAAAATGGACATTAAACTTCAAATTTAACTATATTTAATAAACCCAAACAAAATCCACAACACTCTCTGTCTCAACT

>AtCalS11

TGAGAAAACCCACTTTACGAAGGAATCAAATCGACTCGTTTTCTTAAATCCGCCATAGATCCCATCCGACACACCACCGTCACATCCGGGTTCGAATCCTCCAGCTCGAGCTATTCGCGTTTCTTCATCTTCTCTCTTTGTGCCATGGTGTTTCACCCTCTTTAGGGACATCACAAGCTTTGGTTTGAGAGAATTTGAAATCCTCCTCTAGGATTTGAAAATT

>AtCalS12

AGAAACTGAAACGCGGAAAGGAGGCAAAATCTTCTCGTCGTCGTTGTCGCCGTCTTCAGAGCTACAAACGAAAAAACTCGCTTCCGTTTCGATTTCTCCATTGTTATTGTTTCTTCAGTGAAGCTTTTTTCTTCGAGAAATTTCTAAGATCTACCACATGCTACT

**CLADE 3**

>Lus10001424.g

CTTCACCCAAAGGATCGGATCTCTCTGTCACATGGCGGCTTCCTCCTCTTCTCTCTTTTATACCCTTTCTTCTGACCTACTCCTTTCCTCCTTCTCGCCTCGCCATGCCTTGTACTGCTACTTCATCCAGTTGCTACCTGTGGCGTTAGTTTGTTCTCGTCGATCACTGCCGCTCTGGTATCAGGCGGCGGGAATTCGAA

>Lus10001056.g

GGTGGCAGCCATTCTATTGAACAGTTTCCACATGGCGGCTTCCTCCTCTTCTCTCTTTTATACCCTTTCTTCTGACCTACTCCTTTCCTCCTTCTCGCCTCGCCATGCCTTGTACTGCTACTTCATCCAGTTGCTATCTGTGGCGTTAGTTTGTTCTCGTCGATCACTGCCGCTCTGGTATCAGGCGGCGGGAATTCGAA

>Lus10007327.g

CTCACATGGATTCCTCGTCTTCTCCTCTCTTTTATATACTTTTTGACCACTTCATCTTCCTCCTCCTCTTTATCGCCCCGCAATGCGTTCTACTGCTTTTGCTGCTACTGCTGCTTTCAGTGTTATCTTCCGGACGCGCTAGATATCAGTTGTTGTTCGTCGTCAACGACGACTGCTAGCTGTGCTGATTTGATTTCGAA

>Lus10020750.g

CTCAGGCAAGCATATTAAATTCTGCATGAGAATGTGCACGACACTCTTTGTTCGGCTCTCCTTTGTCGTTTTTAGTTTTCCCTTTGCTAGGTTTCTCTTCGATTTCTGTTTTGGTTCCAATCTCTTATTTCTCTTTGAATTAAATTTAATTCTCTGATTCATCGGAAAAAAAAATAATTCTCCTGTGACACTATATGATA

>UdCalS7

GCTCATATAAAAATCATTCAGCGAAAGATGGTCTTGGAAGAAACCCTACTTTTTCTTGACCTAGTTTTCGATTCCGTATCTGCTACGACGACGAAGAAGAGAGTTTTGTGGTCTCCTCGTGCGAGCAGCG

>CsaCalS7

TAAAGCAAAAGCACTCAAATTCTTCGGTTCGACTCAATACAAAAATTCTCAGTGCACATTCTTTTCTTCCAACCTCAGCAAAAAAATATTCAAAAAAAATCTCACTTTGCTTTTCTTCTTTGCCCCAATTCTTGCCCAAAGTATCTTCCTTTCTCTTTTCCTTACTTTTTCTCCGAGAAATATAAAAGCTCTGATCTCCAATCTCTGAAAAGATGTTTTCTGCTACAATTTTGTATCCAAGGAAGAACGAATTTCAGTCCAAGAAGAAAAAGAAGAAGAAGAAGTGGTTGATTTGTGGTGGTTTTGAGAGGAGTGTTCGCTGAGA

>AtCalS6

AGAAAACCCTTTAGCCATCTTTATCAATCTCTTTTCTTTTGCGTTCTCTATAAAGTTGGTATAAAAGGAGATCAATTAACAAATATGTTAATCGATTACTTTGATTTCAGTGAGATTTTTGATTTCACGAATAAATTATTGGGTTTTTGTGGAGGAGGAGAGGGTGGTAGTAGTAATTATAGTTTCGATTCACGGTGGAT

>AtCalS7

AGAAATCAATTTTTTAGAATATCTCCTTTAGCATTCCTTTCTTCCTCGTAGAGCAATCGCATAACCCTAAAGCCCACTCCACGAATCTAAGCTCCTGTTCCCGCTTCACTTTCCTCTGCACACATGTATATATCCACAAAAAGGGGGAAGAAGAAGATGGGTTTTATCGATTCTTCGGAATTTGAAATTGATCAGCTATT

**CLADE4**

>Lus10042478.g

AAAGCAACTCCCTGCTTTAGCCTTTCTCCTCCTTTCCCATTAAGTAACAAAAGCAACCCTCATTCTCCAATGGCAGCAACAAGATCCAGTCACCCTCACTTTTATATCACCGACTTTCACTATATGGATCCATCTCCTTCGTCATTGTCGTCAACATTTTCCACTCTTACTCAATCTTCCTCTCTCCCTTCCTTCCCGCC

>CsaCalS8

ATTCCACGTAACTCGAAAGCTGACCACGTTAAGCAAACAACAAAGCTATACTTACCAAGAGAAGATCCACTCTATATACTCTGGAATCCCATCTATATCAATTATGTTTCCTTATAAGTACCCTCTCTCATTTCCACTAACAACCCTTTCGAGCTCACTTCACAA

>UdCalS8

TATTCAAGAAAGAATATCCCAACTCACTGTAATATATATACTTTCTATCC

>AtCalS8

CCCATTCATCATTCTCTCTTCAAAAAATTCCGCCATGGAAACTAACCTCACTAAGCAATTTCCTCTTTATATAACTCCTTCCTTCTCCACTCCTCTCTTCCCTCCTTCCGATGCTTCCCTCCAAAACCCCACCGACGACCTAATCCCTAGAACACGAATCAGCTTAATTCGTCTGATTCTGATTGATTTTCTCCCAAAAC

**CLADE 5**

>AtCalS1

TCCTCTCCGGCCTTTTCTCTCTCCTCTCTTCGACCGTCGTCGCCGGAGACCCTTTTCAAGCGGCCAAACAAAGATGCAAGCTTTTTTCTTTAATACTACTGTAGCACTATCGCATCCGATGCTGTTTTAGATTTTGGGTACTCTGCTTCTCTTTCTCTTCATCTTCCTCTGTTCGTCGTCAGCTGAGTTTGATTGTCGGAAAAT

>AtCalS2

ACAAAGACATTGGCGCCTGTCTTGTTCCCGTTGCTTTTCCCCATCTTTATCTCCAATTCTCTCTCTCTCTGTCTCTCTTGTTCTACTACTTTGGGGAAGATGATAGCTTCTTCTTCTTCTCCCTCCATCACTTGTTCTTGATTCTACTTGTGGTCCTCCGCTTCTCTTTTCAGTTTATTTTTAGTAGGATCTAGAAGATC

>AtCalS4

CCTGTTTTGAAAACTTTTGGCACTTGCTTTTTATCATTTTTGATGTTATGTTTTAACTGTTCACAATTGAGGCAAGGCCAGAACCCAGAAGTTTCAGGCCAGAGATTTCCATTATGTTTGACGAATATTTTCATAATGTTTGGTTTCTCAGTTTGGCTGTATGAAAATTGTAAGAGATGCCCCCCAAAAGAAGTGTATCC

>Lus10003920.g

TTCTCTTAATTTTGGTAATCGCCTTAAGTGTTTGCTTCATTTTGATGAAATTTGTTAGCTAAAGACTTAAAGGTGAAATCTTTGGCTGTGTATAGGCTGGATTTTGTACTAGTTTCATTAAAGGCCATCTCTTTTTGTATTTGGTGACATTTCCAAGCTCAGATAGGTCTTGTTGACTTGATCATCCGGAGGAAGTTAAG

>Lus10037469.g

TAATCACCTTAAGGGTTTGCTTCATTTTGATGAAATTTATTAGTTAAAGACTTAAAGGTGACATCTTTGGCTGTGTATAGGCTGGATTTTGTACTAGTTTCATTGAAGGCCATCTCTTGTTGTATTTGGTGACATTTTCCCAAACTCAGATAGATGCAAGTAGGTCTTGTTGACTTGATCATCCGGAGGCAGCAGTCAAG

>UdCalS1

TTTTGTTTTCAATAATAATAAATTCATTGATCTGTTAAATTATTTCTATAATATATATCTTTCTCTTCTTATCATCACCTTGTGGAACGCACTTTCTCTTCATCGTCTTATTTTTTCTTACAGTACACCTTCTGAGATTATAACTCATCAGAAGCCCAGAACCGATACAAAAAAGTCACTGTTACGCTTTTTCGCGGTCGTTATGGAAGTCAAACCATTAGTAGAAGCGCCGATTTGAAAAAGGAAAAGAAAATAATCAAATAGGGAGAGAGTTTCGGAATTTGATTGGTCTCGGAA

>UdCalS3

TCGCCTTCTTTCTTTTGCAAGTAAAGGATAGTAGCTTAAAGTTGCAGCAGAGTGTTTCTCTTTCTTCTCTGCTACTGAGATAGAGAGAGAACAATTTGTCTCTGGTGCATACGATTTTCTCTCTCCTAGGGTTTTGGTTCCGTTCTTGATTCTTATTCACATCTAAACTGGTCTTGTTCTTCCAGCCAAAACGGCTATTGATCCAGACACTCGAGCTGTATATACATCAAATGGTGATTGATTGAGTGCATTGTTTGTTAGTGTTAAGGTCAGTGTGATTTTGCTCTCGGCTAGGGTTTCTGTGCTCGGCAAAGTGAGCTTTGCTCTGTTATTTCTCATCCAGAACAGCGCATGGTCGCGGTAGCGAGGAGTAAGATCCGAGATTTTGTGCCTACAAAGGGTAAAGAGAAGCGTTCGTTTGCTTGTGTGTTTATCGGCGGTCTGAGGTTTTGATCGGAGTGTGGCGGTGAAATAAGGCA

>AtCalS3

AAGGAAGATAAATACAAAAAAACATTCATTTCAGACTGCTCTCTCTCTCTCCCTTTCA CTTTGCGTACTTAGCAGCTAAGTAGCTTAAAAAGTTGTGGCTCCTTTGCTCCGCCATTGTTAGGGTTTCTGGATCTCCGTTTCTCTGGAGGCTGAGGAAACCTTAAAACGATTTAAAACCTGGAGGTTATAGGGTAGACAAA

>Lus10013744.g

TTTTTCTTTTATCTTTTTTCCTCACTTTCTACTTTCATTCTTCTGCTTAGTGCTCTGCCCTTTTGTGTTTGGGTTGTGTTGATTTTGGGATGTGGGTGTGTAAGTTTGAGGGGAAATGGAAGTGGGTAAAGCTGAAACCTTTCCTTTTTCTGCTCTTTTTTTGTCTTGTAGTAAAGGGGGAGTTGCTATTTTGAGAAAAG

>Lus10039199.g

TCTTCATCTTTTTTCCTCACTTTCTACTTTCATTCTTATGCTTCAGTGCTCTGCCCTTTTGCGTTTGGTTGTGTTGATTTTGCGATTTGGGTGTGGTGTGTACGTTTGAGGGGGGAAATGGTAGTGGGTAAAGCTGAAACCTTTCCTTTTCTGCTATTTTTTGTCTTGTAGTAAAGGGGGAGTTGCTATTTTGAGAAAAG

>CsaCalS1-LIKE

TAAACTTTTCTGCTTCTTCTCTCTCTGTTTTGTTTGTTTTGTTACTATAGTCTTTTCATTTTTATGCTTGCAGCCTAAGTTTAAGCTGGTCTTCTTTTTTGAGATTATTTTAATCGATTACCAAAATATCTTCTGGGCTCAGAAAGAGAGTATGGTTCACTTATAAGTTAGAGAAGGGAGCAATTTAAGCTGAATATTACAGAGATTTAGCTGTAAAA

**CLADE 6**

>AtCalS5

GATCTCTCTCTCTCTCCCTTCTTCTTCTTCTTCTTCTTCCTCTCGTCGCCATTTTTTTTTCCTCTGTATCTGTTCATGCCCGACGCCTAACTGAGTCACCTCGTCTCTGCTTCAGGGAAGGGGATCTGAAAGCTCTTCGCATCTAACGGAGAG

>CsaCalS5

TAATGTGTACCGATTTCCATTTTCCTTTCTAGAATTTCTCGACCAGGAAACCAACCTCACGGGTTACAGACGTGTATCTTTAAAAATTGATTTCAGTTCTTGATTTTCTAGTATCATATGGTTTCGAAACTTGGCGCAGGGACAGGGAAATTTTGAAGCGCGCGGAGCAGTATATATCATAAGGCCTCGAGGTTACATCA

>Lus10020893.g

AGCTAATTCAATTCGTTCATGTTTTCCGGCTGCCGGATGTTGTAAAAAACAGGTAGGTAGACGGAGATACAAAGGTGAAATACAATGCAATCATTCTGATCAGCAGTCGAAAAGGCCGCCATCGCCGGATAACGTAACAGAATCGGTAGCTCGGCGCGACGCGGAGGAGAGAAAAATTGATCGGCGATATATATTAACCA
